# Transporter Gene-Mediated Typing for Detection and Genome Mining of Lipopeptide-Producing Pseudomonas

**DOI:** 10.1128/AEM.01869-21

**Published:** 2022-01-25

**Authors:** Léa Girard, Niels Geudens, Brent Pauwels, Monica Höfte, José C. Martins, René De Mot

**Affiliations:** a Centre of Microbial and Plant Genetics, Faculty of Bioscience Engineering, KU Leuven, Heverlee-Leuven, Belgium; b NMR and Structure Analysis Unit, Department of Organic and Macromolecular Chemistry, Faculty of Science, Ghent University, Ghent, Belgium; c Laboratory of Phytopathology, Department of Plants and Crops, Faculty of Bioscience Engineering, Ghent University, Ghent, Belgium; North Carolina State University

**Keywords:** ABC transporter, biosynthetic gene clusters, secondary metabolites

## Abstract

Pseudomonas lipopeptides (LPs) are involved in diverse ecological functions and have biotechnological application potential associated with their antimicrobial and/or antiproliferative activities. They are synthesized by multimodular nonribosomal peptide synthetases which, together with transport and regulatory proteins, are encoded by large biosynthetic gene clusters (BGCs). These secondary metabolites are classified in distinct families based on the sequence and length of the oligopeptide and size of the macrocycle, if present. The phylogeny of PleB, the MacB-like transporter that is part of a dedicated ATP-dependent tripartite efflux system driving export of Pseudomonas LPs, revealed a strong correlation with LP chemical diversity. As each LP BGC carries its cognate *pleB*, PleB is suitable as a diagnostic sequence for genome mining, allowing assignment of the putative metabolite to a particular LP family. In addition, *pleB* proved to be a suitable target gene for an alternative PCR method for detecting LP-producing Pseudomonas sp. and did not rely on amplification of catalytic domains of the biosynthetic enzymes. Combined with amplicon sequencing, this approach enabled typing of Pseudomonas strains as potential producers of a LP belonging to one of the known LP families, underscoring its value for strain prioritization. This finding was validated by chemical characterization of known LPs from three different families secreted by novel producers isolated from the rice or maize rhizosphere, namely, the type strains of Pseudomonas fulva (putisolvin), Pseudomonas zeae (tensin), and Pseudomonas xantholysinigenes (xantholysin). In addition, a new member of the Bananamide family, prosekin, was discovered in the type strain of Pseudomonas prosekii, which is an Antarctic isolate.

**IMPORTANCE** Pseudomonas spp. are ubiquitous bacteria able to thrive in a wide range of ecological niches, and lipopeptides often support their lifestyle but also their interaction with other micro- and macro-organisms. Therefore, the production of lipopeptides is widespread among Pseudomonas strains. Consequently, Pseudomonas lipopeptide research not only affects chemists and microbiologists but also touches a much broader audience, including biochemists, ecologists, and plant biologists. In this study, we present a reliable transporter gene-guided approach for the detection and/or typing of Pseudomonas lipopeptide producers. Indeed, it allows us to readily assess the lipopeptide diversity among sets of Pseudomonas isolates and differentiate strains likely to produce known lipopeptides from producers of potentially novel lipopeptides. This work provides a valuable tool that can also be integrated in a genome mining strategy and adapted for the typing of other specialized metabolites.

## INTRODUCTION

Pseudomonas spp. have the impressive metabolic capacity to biosynthesize a wide diversity of secondary metabolites, going from low-molecular-weight antibiotics and toxins to large amino acid-derived compounds (1, 2). Among those compounds, lipopeptides (LPs) have great potential for biotechnological applications due to their broad antimicrobial properties (antibacterial, antifungal, and antiviral) and/or antiproliferative activities (3). LPs are also involved in important ecological functions, such as swarming motility, biofilm formation, phytopathogenicity, cooperation, or antagonism (3–5). In recent years, research on Pseudomonas LPs has grown rapidly and many studies reported new producer strains of known LPs as well as the characterization of new LPs among single or large sets of environmental Pseudomonas isolate(s) (6–10). The initial detection of LP producer strains often relies on phenotypical tests (e.g., surfactant, antagonistic, or hemolytic activities) or on DNA amplification targeting conserved catalytic domains in their biosynthetic enzymes, complemented with analytical chemistry methodologies (e.g., liquid chromatography-mass spectrometry [LC-MS]). In the last decade, the expansion of sequencing technologies and advances in bioinformatics have led to genome mining-based (or genome-guided) discovery of several new LPs (11–13). Biosynthetic Gene Cluster (BGC) identification enables LP structure prediction that needs to be validated with structural chemistry methodologies to fully characterize new LPs (e.g., MS-MS, nuclear magnetic resonance [NMR], stereochemical assignment methods, and crystallography) (14).

**FIG 1:**
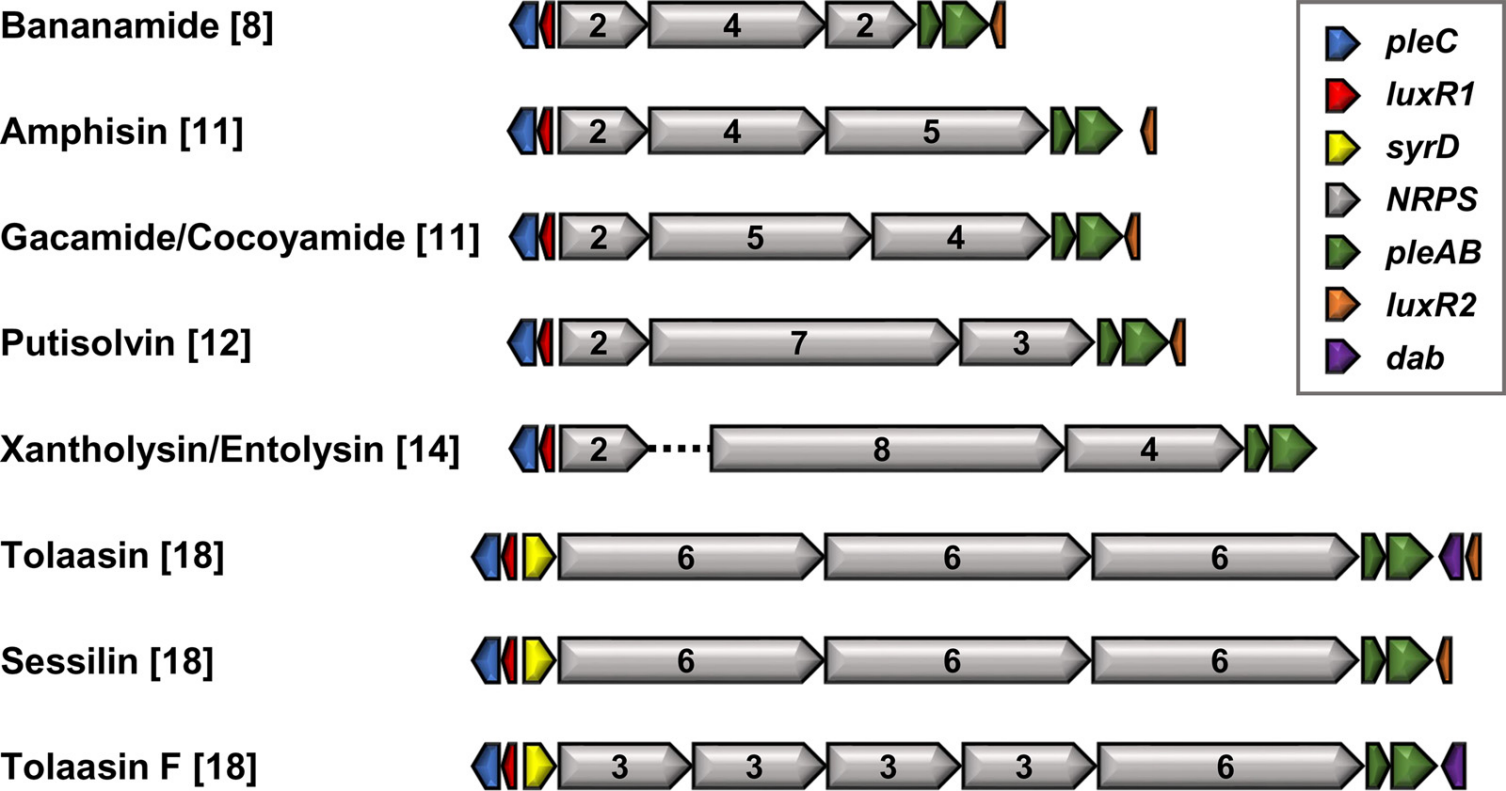
BGC organization for the CLPs from the Bananamide, Amphisin, Gacamide, Putisolvin, Xantholysin, Entolysin, and Tolaasin families. Sessilin is a member of the Tolaasin family. The arrows represent the different genes highlighted in the legend box. Accession numbers are included in Table S1. The total number of modules (in square brackets) and the number of modules encoded by each NRPS gene are indicated. The split BGC organization, represented by a dotted connector line, is also found in the Viscosin [9] and Poaeamide [10] families but not in the Orfamide family [10] (BGCs not shown). A syrD homologue (peptide-3 exporter family, ABC1 superfamily) and an aminotransferase gene for synthesis of 2,4-diaminobutyric acid (dab), present in the Tolaasin BGCs, are also located upstream and downstream, respectively, of Peptin NRPS genes (5). The organization of the Mycin, Peptin, and Factin LPs has been described previously (5).

Pseudomonas LPs consist of an unbranched fatty acid tail attached to a cyclized lipopeptide (CLP) or linear lipopeptide (LLP) and are synthesized by large modular enzyme complexes called nonribosomal peptide synthetases (NRPSs) (4). Briefly, the backbone structure of such NRPSs consists of a string of modules with condensation (C), adenylation (A), and thiolation (T) domains for amino acid addition, selective activation, and substrate transfer between modules, respectively. Most Pseudomonas LP NRPSs lack separate epimerization domains and carry, at the end of the assembly line, a tandem thioesterase (TE) domain, for the release and, in case of CLPs, concomitant cyclization of the lipopeptide. These features distinguish them from the Mycin CLP family (5) as well as from pyoverdine siderophores (2) and lipopeptidic siderophores (15). The modularity of these megaenzymes allows the assembly of a broad diversity of LPs, with variations in the sequence and length of the oligopeptide but also in the size of their macrocycle. Pseudomonas LPs have an oligopeptide length ranging from 8 to 25 amino acids with, for CLPs, a macrocycle size of 4 to 9 amino acids (3, 4). From a genetic point of view, NRPSs are encoded by two to five genes in an operon, or three genes in two clusters (i.e., split organization), with the first gene located elsewhere in the genome (4). These BGCs include an upstream transcriptional regulator gene (*luxR1*), strictly required for LP production, and frequently a second facultative one (*luxR2*) located downstream. In addition, three transporter genes, namely, an *oprM*-like gene (upstream) and a tandem of *macA-macB* homologues (downstream) are conserved, also in case of a split organization (Fig. 1).

The MacB-MacA-TolC machinery, composed of a dimer of the inner membrane ABC transporter MacB, a hexamer of the periplasmic adaptor protein MacA, and a trimer of the outer membrane protein TolC, functions as a tripartite transporter system for drug efflux and export of virulence factors in diverse Gram-negative species (16–18). Among the ubiquitous ABC transporters, MacB is the prototype of type VII featuring a distinctive transmembrane topology and periplasmic domain (19). According to the mechanotransmission model of MacB, it captures periplasmic substrates and expels them through the central channel formed by MacA and TolC by coupling cytoplasmic ATP hydrolysis to large transmembrane and periplasmic conformational changes (19). In Pseudomonas aeruginosa and Pseudomonas putida, the MacB-like PvdT protein constitutes with PvdR (periplasmic component) and OpmQ (outer membrane protein) a similar tripartite export system, encoded by the *pvdRT-ompQ* operon and mediating the secretion and recycling of pyoverdine from the periplasm (20–23). The involvement of MacAB homologues in pseudomonad LP export was first reported for arthrofactin secretion by Pseudomonas sp. MIS38 (ArfDE) (24). Later on, other MacAB homologues (i.e., PseEF and CrpDE) were demonstrated to be essential for the secretion of other LPs within the Peptin and Mycin families (i.e., syringomycin and syringopeptin [25] and corpeptin [26]). However, the outer membrane protein, necessary for the assembly of this tripartite efflux pump, was not characterized in these studies. The *oprM*-like gene, widely conserved within the LP BGCs of Pseudomonas species but not linked to the *macAB*-like operon (Fig. 1), likely encodes the cognate outer membrane component. Like TolC and OpmQ, OprM belongs to the outer membrane efflux protein (OEP) superfamily, featuring a tandem of two OEP domains (Pfam PF02321) (27).

As a MacB dimer initiates the export process upon selective uptake of a periplasmic substrate, we explored the possible link between MacB primary structure and the chemical nature of the exported LP in Pseudomonas. As phylogenetic clustering of MacB amino acid sequences correlated well with the classification by family of LPs, it offers an accessory tool for the initial characterization of LP BGCs revealed by (meta)genomic sequencing. Next, we investigated the possibility of using *macB* for PCR-based detection of the presence of a LP BGC in Pseudomonas environmental isolates, as an alternative for methods targeting NRPS catalytic domains using degenerate primers, which have proven useful for detecting NRPS genes in *Proteobacteria*, including Pseudomonas, *Firmicutes*, *Actinomycetes*, and *Cyanobacteria* (28–32). Two sets of primers were designed based on the LP *macB*-like gene (which we propose to designate as *pleB*) of known producers (i.e., chemically and/or genetically characterized strains) and putative producers identified by genome mining. The primer sets were then used on a large set of Pseudomonas strains (e.g., environmental isolates and type strains) from diverse ecological and geographical origins. We demonstrate that a phylogenetic analysis of the partial *pleB* amplicon sequence enables the assignment of a producer’s strain BGC to a specific LP family and, in combination with structural chemistry, the identification of known and new CLPs.

## RESULTS AND DISCUSSION

### The PleABC system dedicated to LP export in Pseudomonas sp.

A large number of fluorescent Pseudomonas sp. using pyoverdine-mediated iron uptake also synthesize LPs. Consequently, the genomes of LP-producing Pseudomonas sp. harbor genes encoding at least two MacB-type tripartite efflux pumps (i.e., for pyoverdine and LP export). By exploring the synteny of these genomes, one can clearly differentiate these two export systems; one system is encoded by the single *pvdRT-opmQ* operon and the second by *macAB* and *oprM* homologues located at the distal and proximal LP BGC border, respectively (Fig. 1). Overall, the strong conservation of this tripartite efflux system suggests that LP-dedicated transporter genes *macAB*-*oprM* have coevolved with the respective NRPS genes in Pseudomonas sp. On the other hand, as the number of genetically characterized Pseudomonas LP NRPS BGCs is constantly increasing, different names have emerged for these LP *macAB* operons, such as *arfDE* for arthrofactin (24), *xtlDE* for xantholysin (33), *banDE* for bananamide (6), and *wlpDE* for white line-inducing principle (WLIP) (34), among others, obscuring that they encode very similar components of the same type of Pseudomonas export system dedicated to the secretion of LPs. Therefore, in order to clearly distinguish the components of the LP-specific export system, we introduce here the generic name Pseudomonas lipopeptide export proteins A, B, and C (PleA, PleB, and PleC, respectively) to designate the periplasmic adapter (MacA), inner membrane ABC transporter (MacB), and the outer membrane protein (OprM), respectively.

### PleB phylogeny correlates with transporter substrate specificity.

**FIG 2:**
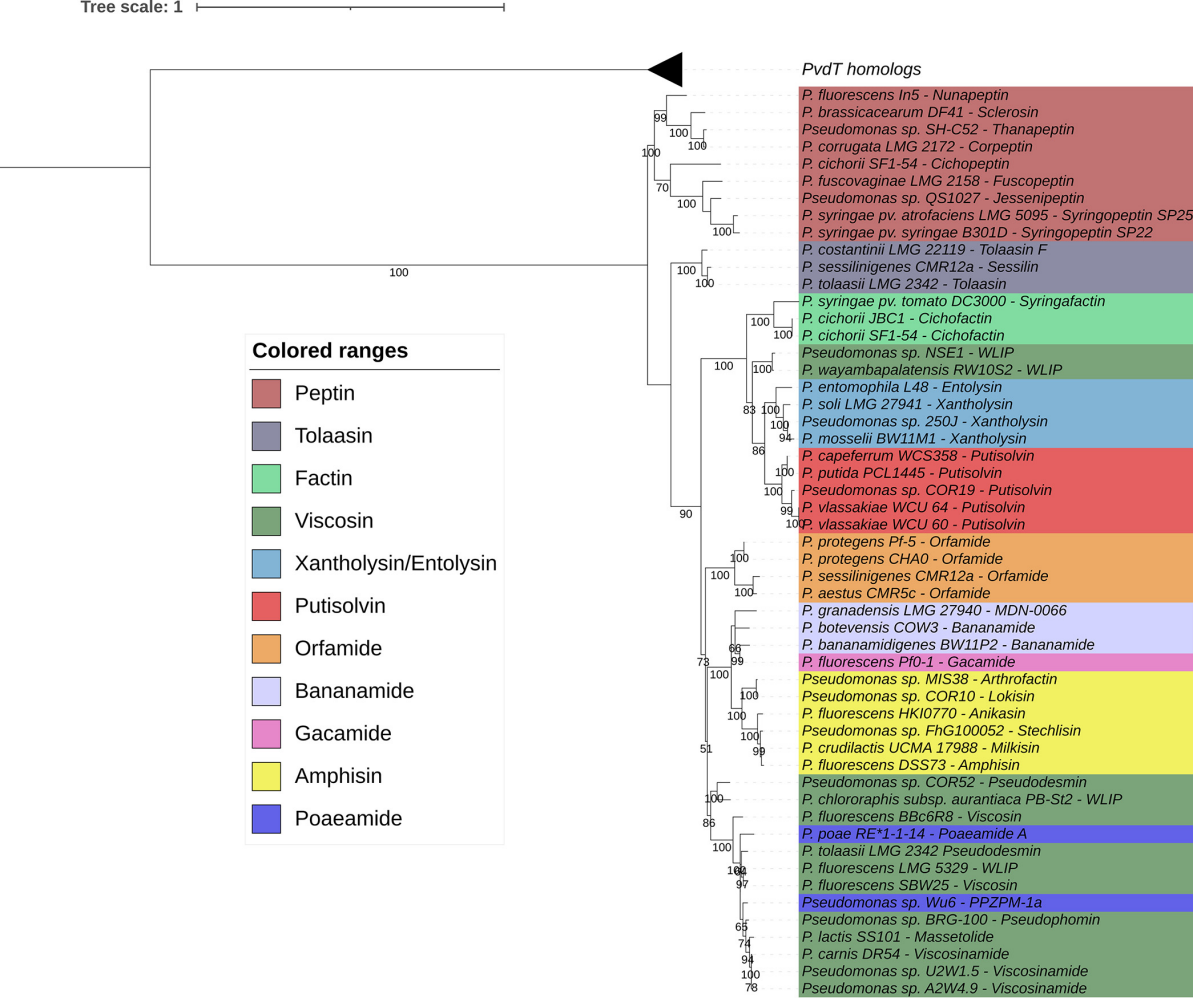
Phylogenetic tree based on full MacB-like proteins (PleB) of known LP producers (PvdT and PleB; ∼ 660 amino acids [aa]). Maximum likelihood tree constructed using the JTT+F+I+G model (MEGA-X). Bootstrap values were calculated based on 1,000 replications, and only bootstrap values higher than 50% are indicated. The PvdT homolog branch is collapsed, and an expanded version of this tree is shown in the supplemental material (Fig. S1).

Assuming that PleB fulfills a similar role as other MacB proteins in substrate binding, it was examined whether PleB diversity may somehow reflect LP structural diversity. Using the PleB amino acid sequences retrieved from 53 characterized LP producers and the corresponding PvdT proteins, when available in public databases (Table 1; see Table S1 in the supplemental material), as an outgroup, a phylogeny of these MacB-related transporters was constructed (Fig. 2; see Fig. S1 in the supplemental material). First, the PvdT homologs are collectively clearly separated from the PleB proteins, representing two paralogous clades and concordant with the fact that the respective tripartite efflux pumps are dedicated to the transport of two different substrate classes. Indeed, the pyoverdine- and LP-associated MacB homologs belong to the same transporter family but constitute two distinct subfamilies with 44% to 48% identity between the paralogs (Fig. 2 and Fig. S1). Among the PleB homologues, the most divergent ones still share about 70% identity and homology between those of the same family typically exceeds 90% identity. Somewhat lower values (>65% overall identity and >85% intrafamily identity, respectively) are found among both PleA and PleC homologues. For PvdR and OpmQ, the respective pyoverdine-associated paralogues of PleA and PleC, interclade sequence identities are below 40%.

**TABLE 1:** Characterized LP-producing Pseudomonas sp.^*a*^

LP family	LP	Peptide length (amino acids)	Macrocycle size (amino acids)	Species	Strain	Reference(s)
Factin	Cichofactin	8	Linear	*P. cichorii*	SF1-54	47
				*P. cichorii*	JBC1	12
	Syringafactin			P. syringae pv. tomato	DC3000	48
	Virginiafactin			Pseudomonas sp.	QS1027	12
Bananamide	MDN-0066	8	6	*P. granadensis*	**LMG 27940^T^**	49
	Bananamide I-III (A-C)			*P. bananamidigenes*	**BW11P2^T^**	8
	Bananamide D-G			*P. botevensis*	**COW3^T^**	6
Viscosin	Viscosin	9	7	P. fluorescens	BBc6R8	50, 51
				P. fluorescens	SBW25	52
	Viscosinamide			*P. carnis*	DR54	53
				Pseudomonas sp.	A2W4.9	54
				Pseudomonas sp.	U2W1.5	54
	WLIP			P. chlororaphis subsp. *aurantiaca*	PB-St2	55
				*P. wayambapalatensis*	RW10S2	34
				Pseudomonas sp.	NSE1	46
				P. fluorescens	LMG 5329	56
	Massetolide			*P. lactis*	SS101	57, 58
	Pseudodesmin			Pseudomonas sp.	COR52	54
				*P. tolaasii*	NCPPB 2192^T^ = LMG 2342^T^	59
	Pseudophomin			Pseudomonas sp.	BRG-100	60, 61
Orfamide	Orfamide	10	8	P. protegens	Pf-5	11
				P. protegens	CHA0^T^	62, 63
				*P. aestus*	CMR5c	63
				*P. sessilinigenes*	CMR12a^T^	64
Poaeamide	Poaeamide A	10	8	*P. poae*	RE*1-1-14	65
	Poaeamide B			*P. synxantha*	CR32	8
	PPZPM			Pseudomonas sp.	Wu6	35
Amphisin	Amphisin	11	9	P. fluorescens	**DSS73**	66, 67
	Anikasin			P. fluorescens	HKI0770	13
	Arthrofactin			Pseudomonas sp.	**MIS38**	24, 68
	Lokisin			Pseudomonas sp.	DSS41	69
				Pseudomonas sp.	**COR10**	45
	Milkisin			*P. crudilactis*	**UCMA 17988^T^**	46
	Tensin-stechlisin			Pseudomonas sp.	FhG100052	10
Gacamide	Gacamide	11	5	P. fluorescens	**Pf0-1**	9
	Cocoyamide			Pseudomonas sp.	**COW5**	7
Putisolvin	Putisolvin I-II	12	4	P. putida	PCL1445	70, 71
				*P. capeferrum*	**WCS358**	8
	Putisolvin III-V			Pseudomonas sp.	**COR19**	39
				*P. vlassakiae*	**WCU_60**	39
				*P. vlassakiae*	**WCU_64**	39
Xantholysin	Xantholysin	14	8	*P. mosselii*	**BW11M1**	33, 72
				*P. soli*	**LMG 27941^T^**	73
				Pseudomonas sp.	250J	74
Entolysin	Entolysin	14	5	*P. entomophila*	**L48**	75
Tolaasin	Tolaasin I	18	5	*P. tolaasii*	**NCPPB 2192^T^ = LMG 2342^T^**	59
	Tolaasin F			*P. costantinii*	**DSM 16734^T^ = LMG 22119^T^**	76
	Sessilin			*P. sessilinigenes*	**CMR12a^T^**	64
Peptin	Syringopeptin SP22	22	8	P. syringae pv. syringae	B301D	77, 78
	Syringopeptin SP25	25	8	P. syringae pv. atrofaciens	NCPPB 2612^T^ = LMG 5095^T^	79, 80
	Cichopeptin	22	5	*P. cichorii*	SF1-54	81
	Fuscopeptin	19	5	*P. fuscovaginae*	LMG 2158^T^	5
	Jessenipeptin	19	5	Pseudomonas sp.	QS1027	82
	Nunapeptin	22	5	P. fluorescens	In5	83
	Corpeptin	22	5	*P. corrugata*	NCPPB 2445^T^ = LMG 2172^T^	84
	Thanapeptin	22	5	Pseudomonas sp.	SH-C52	85
	Sclerosin	22	Linear	P. brassicacearum	DF41	86

a Strains used for primer validation are highlighted in bold. The accession numbers for NRPS genes and PleB and PvdT homologs are provided in Table S1. Tensin congeners produced by strain FhG100052 were designated stechlisins (10).

Most Pseudomonas strains carry only one LP BGC and associated *pleB* gene, but some strains can produce more than one LP. As shown in Fig. 2 for Pseudomonas cichorii SF1-54, Pseudomonas tolaasii LMG 2342, and Pseudomonas sessilinigenes CMR12a, two distinct PleB proteins are encoded by the *pleAB* operons associated with two different BGCs (for cichopeptin-cichofactin, tolaasin-pseudodesmin, and sessilin-orfamide, respectively), and suitable primers should be able to selectively amplify the respective transporter genes. However, only a single transporter gene appears to be linked to one of two BGCs in Pseudomonas sp. QS1027. A *pleAB* gene pair is absent from its virginiafactin BGC and only linked to its jessenipeptin NRPSs (5). This information suggests that one transporter may export two different LPs, but this suggestion awaits experimental demonstration.

The phylogeny based on the full PleB protein allows one to clearly distinguish family-specific clusters of the LP transporters from the Peptin, Tolaasin, Factin, Xantholysin-Entolysin, Putisolvin, Orfamide, and Amphisin families, with intrafamily sequence identities ranging from 99.9% to 79.5%. The similarity between the entolysin and xantholysin PleBs, both involved in secretion of a lipotetradecapeptide but differing by macrocycle size, is in line with the previously highlighted relatedness of their NRPS systems (33). Both poaeamide-PPZPM transporters are phylogenetically intertwined with the closely related PleB proteins of the Viscosin family, which correlates with the pronounced peptide sequence similarity between members of both LP families. However, compared with the seven-membered macrocycle in the nonapeptidic Viscosin LPs, the Poaeamide decapeptides feature a macrocycle of eight residues (35). The tight clustering between the Gacamide and Bananamide PleB sequences cannot be attributed to such structural similarity of peptide length and sequence. However, these two LP families were shown to be significantly associated with the same taxonomic clade (i.e., Pseudomonas koreensis subgroup) (6, 7). The WLIP exporters of strains RW10S2 and NSE1 cluster outside the main Viscosin transporter clade. Again, this finding is consistent with previously established taxonomic affiliations of both strains in the P. putida group, along with Entolysin, Xantholysin, and Putisolvin producers, while the other members of the Viscosin family belong to the P. fluorescens group (7, 36).

Our observation that the sequence diversity of the PleB proteins reflects the substrate specificity of the dedicated tripartite efflux system for LP export is in line with a comprehensive computational study that highlighted the value of BGC-associated transporter gene analysis for metabolite prediction (27). In that study, the MacB export system was proposed as a good indicator of antimicrobial BGCs based on a comparison of the biological activities identified for the corresponding metabolites. Transporter gene colocalization with biosynthetic genes may be driven primarily by coinheritance and coregulation (37). These observations suggest that transporter gene-derived information can be of value in the initial characterization of biosynthetic systems for certain chemical classes of specialized metabolites.

### Targeting *pleB* for LP BGC prospection.

Focusing on LPs typically associated with a nonphytopathogenic lifestyle, in this study, we targeted the recently expanded Bananamide and Amphisin families, and the less populated Gacamide, Putisolvin, Entolysin, Xantholysin and Tolaasin families. Until now, within these families, a total of 23 known producers have been genetically and/or chemically characterized, as follows: Bananamide (*n* = 3), Amphisin (*n* = 6), Gacamide (*n* = 2), Putisolvin (*n* = 5), Xantholysin (*n* =3), Entolysin (*n* = 1), and Tolaasin (*n* = 3) (Table 1). In order to better seize the natural diversity within these LP families, we included genome mining as a first step. Based on the general features of the Pseudomonas LP NRPS systems, we retrieved 75 Pseudomonas strains harboring complete syntenic BGCs with NRPS homologues matching the family-specific number of modules (see Table S2 in the supplemental material). To illustrate the known and potentially expanded diversity present among these sets of Pseudomonas isolates, the phylogenetic relationship between characterized and predicted LP producers, based on concatenated NRPS enzymes, is shown in Fig. S2 to S7 in the supplemental material. In order to assess this diversity experimentally, two sets of primers were designed using the *pleB* gene of the 23 known and 75 potential LP producers, including type strains of different species (Table S1 and S2). The first primer pair BanAmpGac F/R, targeting the Amphisin, Bananamide, and Gacamide families, and based on 61 *pleB* sequences (10 known and 51 putative), leads to the amplification of an ∼1,300-bp fragment. The second primer pair PutXanTol F/R, aiming at the Putisolvin, Tolaasin, Xantholysin, and Entolysin families, and based on 36 *pleB* sequences (12 known and 24 putative), generates an amplicon of about 1,000 bp.

Both primer sets were then validated by amplification of part of the *pleB* gene from a selection of known and putative LP producers (highlighted in Table 1 and S2). The results confirmed the expected sizes of the amplicons (see Fig. S8 in the supplemental material). To test the specificity of these primers, amplifications were conducted on a selection of 10 established producers of other LP families (i.e., Factin, Viscosin, Orfamide, and Poaeamide), and we were able to amplify the *pleB* of 9 strains out of 10 with one or both primer sets (see Table S3 in the supplemental material). Therefore, these two primer sets should allow the detection of some LP producers outside the initially targeted families.

The two sets have been designed in order to amplify overlapping portions of the *pleB* gene, as shown in Fig. S9 and S10 in the supplemental material, yielding a common region of about 880 bp. In order to evaluate how these 880-bp internal *pleB* fragments relate to the diversity of the NRPS systems assembling the respective PleB substrates, we calculated, per LP family, the correlation between the *pleB* nucleotide identity and concatenated NRPS amino acid identities. For this process, a total of 94 Pseudomonas strains (20 known and 74 putative LP producers) were included (i.e., Bananamide [*n* = 21], Amphisin [*n* = 32], Gacamide [*n* = 8], Putisolvin [*n* = 16], Xantholysin/Entolysin [*n* = 8], and Tolaasin [*n* = 9]), and we obtained Pearson correlation coefficients ranging from 0.7910 and 0.9607 (*P* < 10e-7) (Fig. S2 to S7). The 880-bp fragment of the *pleB* gene covers the MacB periplasmic core domain (PCD; Pfam PF12704), which comprises the predicted portal initiating selective export (18, 19, 38). Consequently, it is not surprising to have a high correlation between the concatenated NRPS sequences, reflecting the substrate diversity, and this portion of the *pleB* gene encoding the moiety for LP product uptake. Altogether, these findings support the use of the *pleB* gene as a proxy for LP structural diversity.

Our two sets of primers were further used to detect LP producers among 126 Pseudomonas strains from our in-house collections (i.e., 117 environmental and 9 type strains) covering diverse ecological and geographical origins. We were able to amplify and sequence partial *pleB* amplicons for 92 Pseudomonas strains. Among those strains, 32 genome-sequenced strains represented 22 different species based on genomic comparison to type strains, indicating substantial taxonomic diversity among the amplicon-positive isolates. No amplification could be obtained for the 34 remaining strains. However, the lack of *pleB* amplification should be interpreted with care as it not only may be due to the absence of LP BGC but also can be caused by the specificity of both primer sets (e.g., no *pleB* amplification in the xantholysin and putisolvin producers WCA_13, WCA_17, NNC7, and COW62). In contrast, no cases of unspecific amplifications have been observed. The responses with each set of primers together with the accession numbers of the partial *pleB* amplicons are displayed in Table S4 in the supplemental material. This table also specifies the environmental strains for which there was information available from previous studies about the LP product; these studies evaluated CLP diversity among large sets of environmental Pseudomonas isolates (49 out of 92 strains, Table S4) (6, 7, 39).

### Predictive value of *pleB*-based phylogenetic analysis.

**FIG 3:**
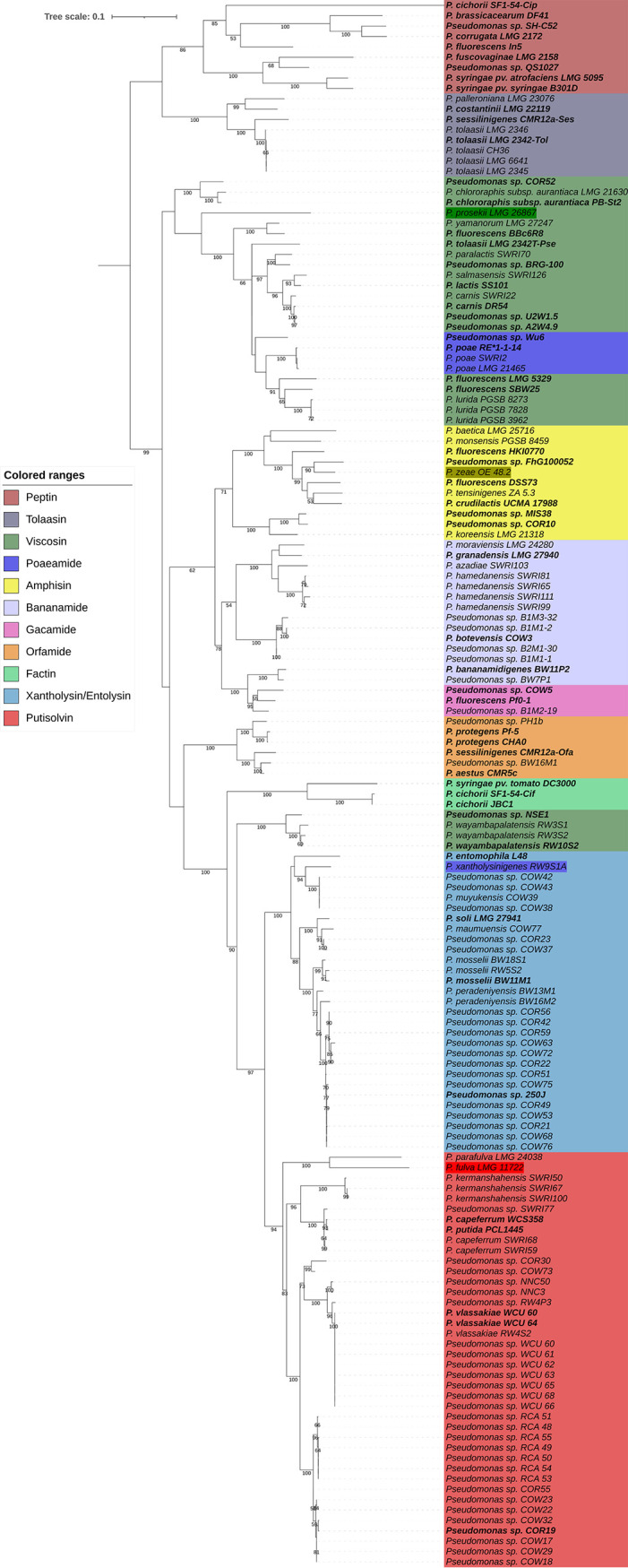
Maximum likelihood phylogenetic tree based on partial *pleB* genes of the 54 known and 92 newly detected LP-producing Pseudomonas strains (Table 1, S2, and S4). The tree was constructed using the GTR+G+I model (MEGA-X). Bootstrap values were calculated based on 1,000 replications, and only bootstrap values higher than 50% are indicated. Known LP producers (chemically and/or genetically characterized) are highlighted in bold. Pseudomonas strains selected for chemical characterization are highlighted in darker color shades.

The phylogenetic tree based on the partial *pleB* sequences of both known LP producers (*n* = 53) (Table 1 and S1) and those tentatively identified in this study (*n* = 92) (Table S4) is presented in Fig. 3. The clustering of known LP producers based on the partial *pleB* gene corresponds to the previously described phylogeny based on full PleB proteins (Fig. 1), allowing the discrimination between the different LP families with exceptions within the Viscosin and Poaeamide families. Among the 92 Pseudomonas strains with a sequenced *pleB* fragment, 77 were identified as potential LP producers from the initially targeted families Bananamide (*n* = 11), Amphisin (*n* = 3), Gacamide (*n* = 1), Putisolvin (*n* = 33), Xantholysin (*n* = 25), and Tolaasin (*n* = 4). In addition, 13 strains were linked to other LP families (i.e., Viscosin [*n* = 10], Orfamide [*n* = 1], and Poaeamide [*n* = 2]). These affiliations are consistent with results of previous studies that evaluated CLP diversity among large sets of environmental Pseudomonas isolates (49 out of 92 strains) (Table S3) (6, 7, 39). We were also able to confirm these affiliations by identifying syntenic BGCs in several strains with a (draft) genome available in public databases (34 out of 92). A detailed overview with the accession numbers for their NRPSs are indicated in Table S4. Overall, we were able to confirm the reliability of this methodology (90% of our *pleB*-based affiliation were validated) for the affiliation of producers to known LP families. A prominent deviation, however, is the BGC of *P. prosekii* LMG 26287^T^, clustering within the Viscosin family based on its *pleB* but assigned to the Bananamide family when the BGC organization and module composition of its NRPSs are considered.

### Chemical characterization of LPs.

We already highlighted the high level of congruence between the *pleB* and concatenated NRPS phylogenies. Thus, one can expect that, within a LP family, strains harboring a highly similar *pleB* would produce the same LP and, conversely, those with divergent *pleB* would also carry a divergent NRPS system, which could potentially lead to (i) the identification of additional species producing a known LP, expanding genetic and biochemical diversity, or (ii) the characterization of new LPs, broadening chemical diversity. To evaluate the potential of this approach, four strains harboring *pleB* with different levels of divergence from known LP producers in four different families (indicated in dark color shades, Fig. 3) were selected for chemical characterization. Two of these taxonomically distinct strains belong to the P. fluorescens group, namely, *P. prosekii* LMG 26867^T^ (Bananamide; Pseudomonas mandelii subgroup) and *P. zeae* OE 48.2^T^ (Amphisin; *P. koreensis* subgroup), whereas Pseudomonas xantholysigenes RW9S1A^T^ (Xantholysin; Pseudomonas mosselii subgroup) and *P. fulva* LMG 11722^T^ (Putisolvin; P. putida subgroup) are affiliated with the P. putida group (40).

To verify their LP production, crude extracts were purified and single LPs were collected and subjected to liquid-state NMR spectroscopy. Results obtained from 2D total correlation spectroscopy (TOCSY) and 2D ^1^H-^13^C heteronuclear single quantum coherence (HSQC) spectroscopy allowed the identification of the (3-hydroxy) fatty acid and the identity of individual amino acids while the amino acid sequence of each LP was confirmed by the analysis of the 2D rotating-frame nuclear Overhauser effect spectroscopy (ROESY) and ^1^H-{^13^C} gradient-selected heteronuclear multiple-bond coherence (gHMBC) spectra (see Fig. S12 to S16 in the supplemental material; Table 2). In some cases, the position of the ester bond, responsible for the cyclization of the peptide, could be confirmed by ^1^H-{^13^C} gHMBC analysis. In the case of insufficient signal-to-noise or spectral overlap, the unusually high chemical shifts of the Ser or Thr CH^β^ involved in ester bond formation were used as an indication that these residues are involved in the depsi bond and thus allowed us to confirm the size of the macrocycles (Table 2). Finally, the length of the fatty acid could be determined based on LC-MS data and by taking into account the results from NMR.

**TABLE 2:** Amino acid sequences of known and newly characterized LPs from the Bananamide, Amphisin, Putisolvin, and Xantholysin families^*a*^

LP family	LP	Strain	Fatty acid	Amino acid by position														Reference(s)
				1	2	3	4	5	6	7	8	9	10	11	12	13	14	
Bananamide	MDN-0066	*P. granadensis* LMG 27940^T^	3-OH C_10:0_	Leu	Glu	Thr	Leu	Leu	Ser	Leu	Ile							49
	Bananamide I (A)	*P. bananamidigenes* BW11P2^T^	3-OH C_12:0_	Leu	Asp	Thr	Leu	Leu	Gln	Leu	Ile							8
	Bananamide II (B)		3-OH C_10:0_	Leu	Asp	Thr	Leu	Leu	Gln	Leu	Ile							
	Bananamide III (C)		3-OH C_12:1_	Leu	Asp	Thr	Leu	Leu	Gln	Leu	Ile							
	Bananamide D	*P. botevensis* COW3^T^	3-OH C_12:1_	Leu	Asp	Thr	Leu	Leu	Ser	Leu	Ile							6
	Bananamide E		3-OH C_12:0_	Leu	Asp	Thr	Leu	Leu	Ser	Leu	Ile							
	Bananamide F		3-OH C_10:0_	Leu	Asp	Thr	Leu	Leu	Ser	Leu	Ile							
	Bananamide G		3-OH C_12:1_	Leu	Asp	Thr	Leu	Leu	Ser	Leu	Val							
	Prosekin	*P. prosekii* LMG 26867^T^	3-OH C_10:0_	Leu	Glu	Thr	Leu	Leu	Ser	Ile	Ile							This study
Amphisin	Amphisin	P. fluorescens DSS73	3-OH C_10:0_	Leu	Asp	Thr	Leu	Leu	Ser	Leu	Gln	Leu	Ile	Asp				67
	Anikasin	P. fluorescens HKI0770	3-OH C_10:0_	Leu	Asp	Thr	Leu	Leu	Ser	Leu	Ser	Leu	Ile	Asp				13
	Lokisin	Pseudomonas sp. DSS41																69
	Arthrofactin	Pseudomonas sp. MIS38	3-OH C_10:0_	Leu	Asp	Thr	Leu	Leu	Ser	Leu	Ser	Ile	Ile	Asp				68
	Milkisin	*P. crudilactis* UCMA 17988^T^	3-OH C_10:0_	Leu	Asp	Thr	Leu	Leu	Ser	Leu	Gln	Leu	Ile	Glu				46
	Stechlisin B2	Pseudomonas sp. FhG100052	3-OH C_8:0_	Leu	Asp	Thr	Leu	Leu	Ser	Leu	Gln	Leu	Ile	Glu				10
	Stechlisin C3		3-OH C_10:0_	Leu	Asp	Thr	Leu	Leu	Ser	Leu	Gln	Leu	Val	Glu				
	Stechlisin D3		3-OH C_10:0_	Leu	Asp	Thr	Leu	Leu	Ser	Leu	Gln	Leu	Leu	Glu				
	Stechlisin E2		3-OH C_10:0_	Leu	Glu	Thr	Leu	Leu	Ser	Leu	Gln	Leu	Ile	Glu				
	Stechlisin F		3-OH C_12:0_	Leu	Asp	Thr	Leu	Leu	Ser	Leu	Gln	Leu	Ile	Glu				
	Tensin	Pseudomonas sp. FhG100052	3-OH C_10:0_	Leu	Asp	Thr	Leu	Leu	Ser	Leu	Gln	Leu	Ile	Glu				10
		*P. zeae* OE 48.2^T^																This study
Putisolvin	Putisolvin I	P. putida PCL1445	C_6:0_	Leu	Glu	Leu	Ile	Gln	Ser	Val	Ile	Ser	Leu	Val	Ser			70
	Putisolvin II		C_6:0_	Leu	Glu	Leu	Ile	Gln	Ser	Val	Ile	Ser	Leu	Ile	Ser			
	Putisolvin III	Pseudomonas sp. COR19	C_6:0_	Leu	Glu	Leu	Leu	Gln	Ser	Val	Leu	Ser	Leu	Val	Ser			39
	Putisolvin IV	*P. vlassakiae* WCU_64	C_6:0_	Leu	Glu	Leu	Leu	Gln	Ser	Val	Leu	Ser	Leu	Ile	Ser			
	Putisolvin V	*P. fulva* LMG 11722^T^	C_6:0_	Leu	Glu	Leu	Leu	Gln	Ser	Val	Leu	Ser	Leu	Leu	Ser			This study
Xantholysin	Xantolysin A	*P. mosselii* BW11M1	3-OH C_10:0_	Leu	Glu	Gln	Val	Leu	Gln	Ser	Val	Leu	Gln	Leu	Leu	Gln	Ile	33
		*P. soli* LMG 27941^T^																73
		Pseudomonas sp. 250J																74
		*P. xantholysinigenes* RW9S1A^T^																This study

a Amino acids included in the macrocycle are higlighted in light gray. The additional tensin congeners produced by strain FhG100052 were designated stechlisins (10).

**FIG 4:**
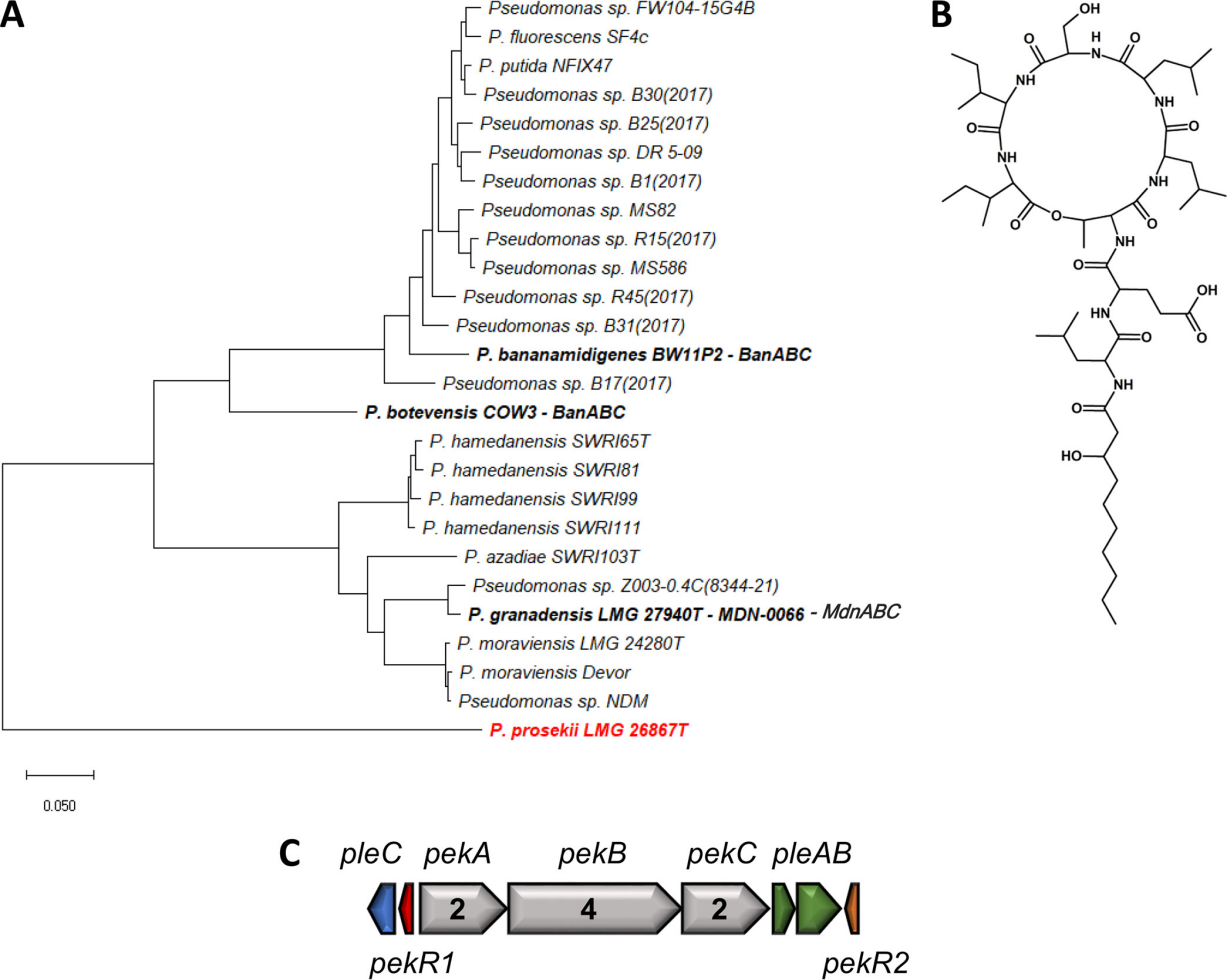
Genetic and chemical characterization of *P. prosekii* LMG 26867^T^. (A) Phylogenetic positioning of *P. prosekii* LMG 26867^T^ based on concatenated NRPS proteins, including known and newly identified members of Bananamide family. Maximum likelihood tree constructed using the JTT+F+I+G model (MEGA-X). (B) Chemical structure of prosekin. (C) Prosekin BGC, PleC (SDT49830), PekR1 (SDT49844), PekA (SDT49871), PekB (SDT49885), PekC (SDT49904), PleA (SDT49920), PleB (SDT49938), and PekR2 (SDT49952).

*P. prosekii* LMG 26867^T^, isolated from soil in James Ross Island in Antarctica, produced a novel LP designated prosekin (Fig. 4B and S12; see Table S5 in the supplemental material). This compound features an amino acid sequence composed of 8 amino acids, of which 6 are involved in the macrocycle, and thus structurally belongs to the Bananamide family. Prosekin has a saturated fatty acid moiety, 3-hydroxydecanoate (3-OH C10:0). Compared with the family’s namesake LP bananamide, prosekin features an Ile at position 7 (instead of Leu), a Glu at position 2 like MDN-0066 (instead of Asp for bananamides A to G) and a Ser at position 6 (instead of Gln for bananamides A to C). Consequently, it represents a novel LP within the Bananamide family (Table 2).

**FIG 5:**
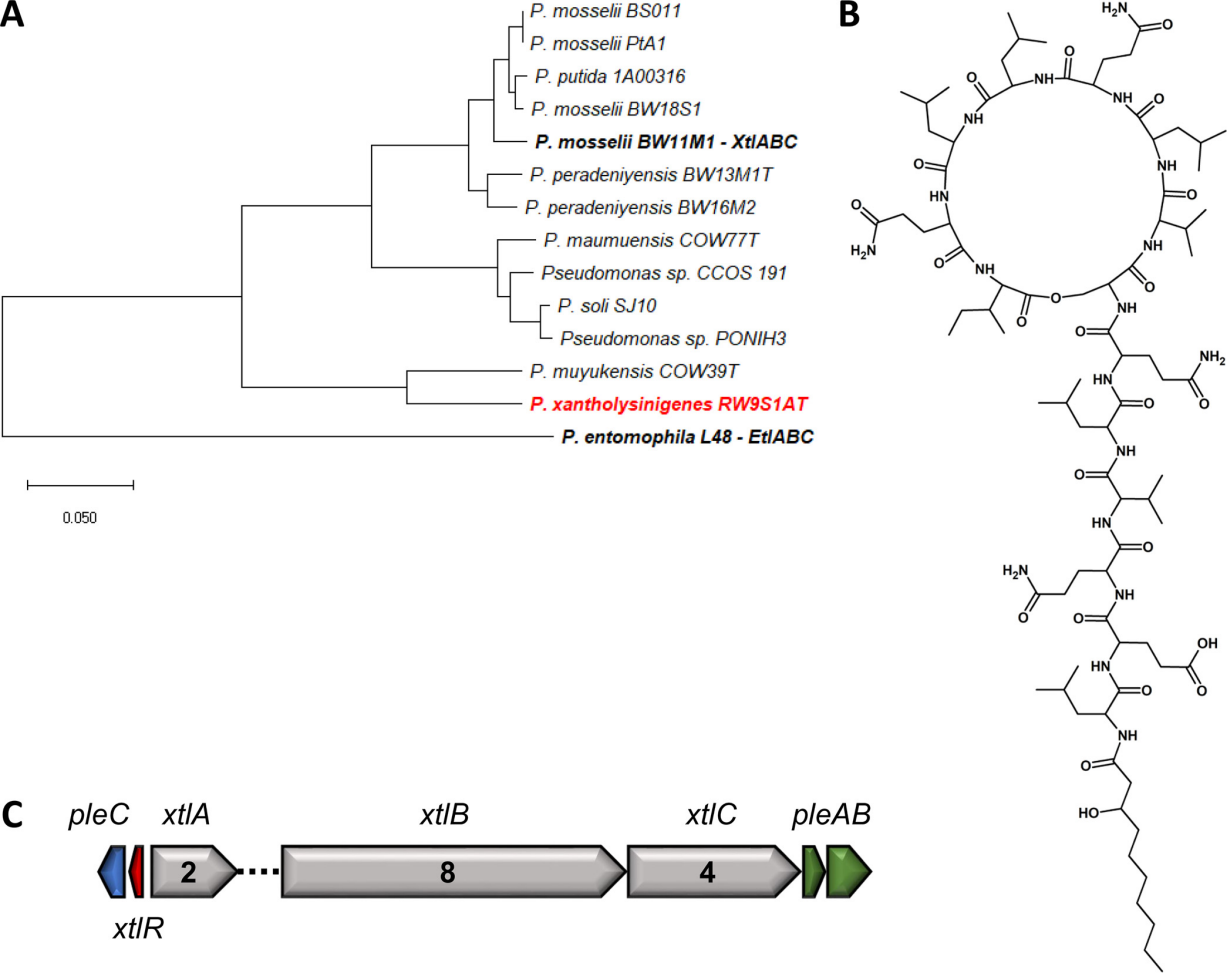
Genetic and chemical characterization of *P. xantholysinigenes* RW9S1A^T^. (A) Phylogenetic positioning of *P. xantholysinigenes* RW9S1A^T^ based on concatenated NRPS proteins, including known and newly identified members of the Xantholysin and Entolysin families. Maximum likelihood tree constructed using the JTT+F+I+G model (MEGA-X). (B) Chemical structure of xantholysin A. (C) Xantholysin BGC, PleC (QXI40743), XtlR (QXI40744), XtlA (QXI40745), XtlB (QXI36232), XtlC (QXI36231), PleA (QXI36230), and PleB (QXI36229).

**FIG 6:**
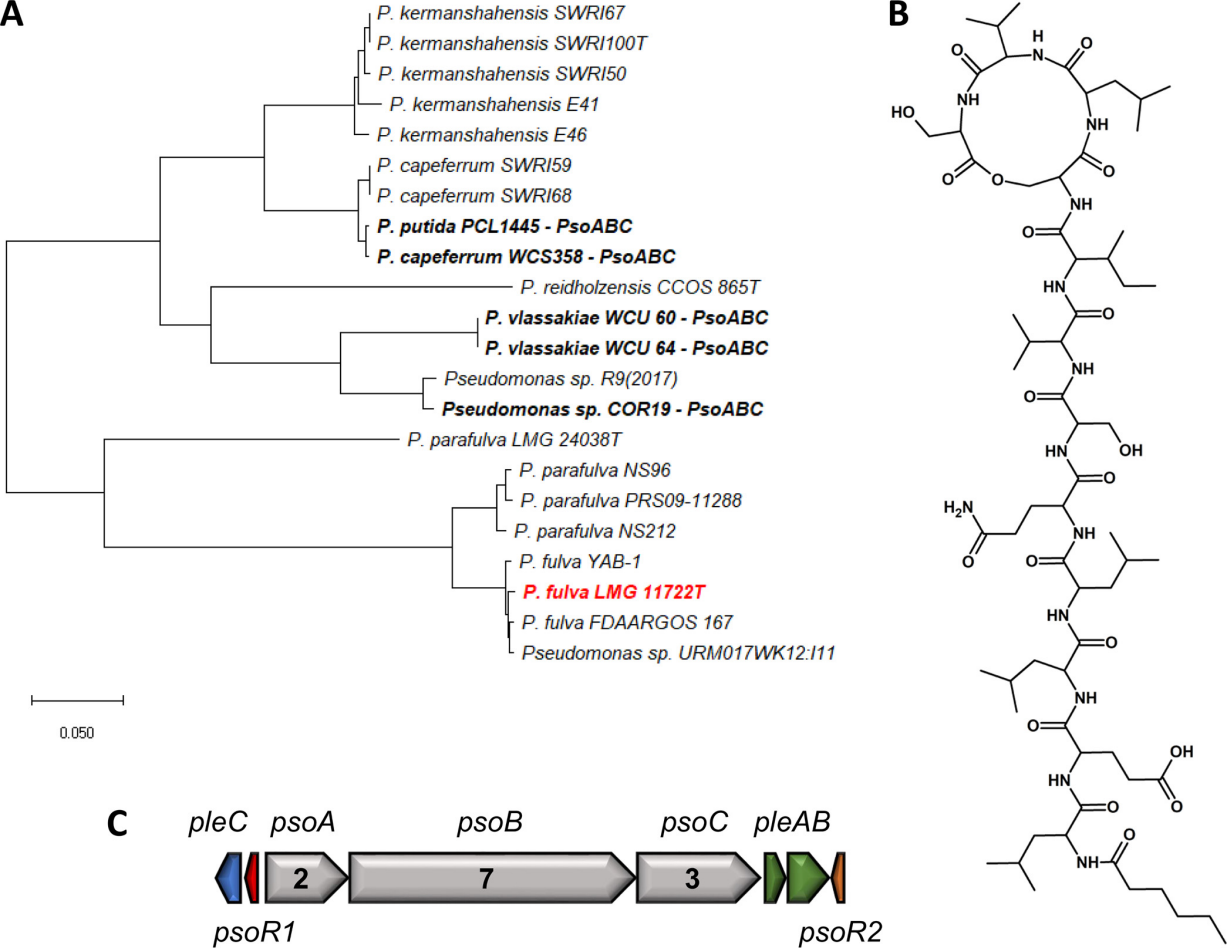
Genetic and chemical characterization of *P. fulva* LMG 11722^T^. (A) Phylogenetic positioning of *P. fulva* LMG 11722^T^ based on concatenated NRPS proteins, including known and newly identified members of the Putisolvin family. Maximum likelihood tree constructed using the JTT+F+I+G model (MEGA-X). (B) Chemical structure of putisolvin III. (C) Putisolvin BGC, PleC (WP_028687799), PsoR1 (WP_042139415), PsoA (WP_033737094), PsoB (WP_042139413), PsoC (WP_042139412), PleA (WP_027915637), PleB (WP_042139409), and PsoR2 (WP_027915635).

Using an identical methodology, *P. xantholysinigenes* RW9S1A^T^ and *P. fulva* LMG 11722^T^ were found to produce previously characterized LPs, namely, xantholysin A (Fig. 5B and S13; see Table S6 in the supplemental material) and putisolvins III, IV, and V (Fig. 6B and S14; see Table S7 to S9 in the supplemental material), respectively.

**FIG 7:**
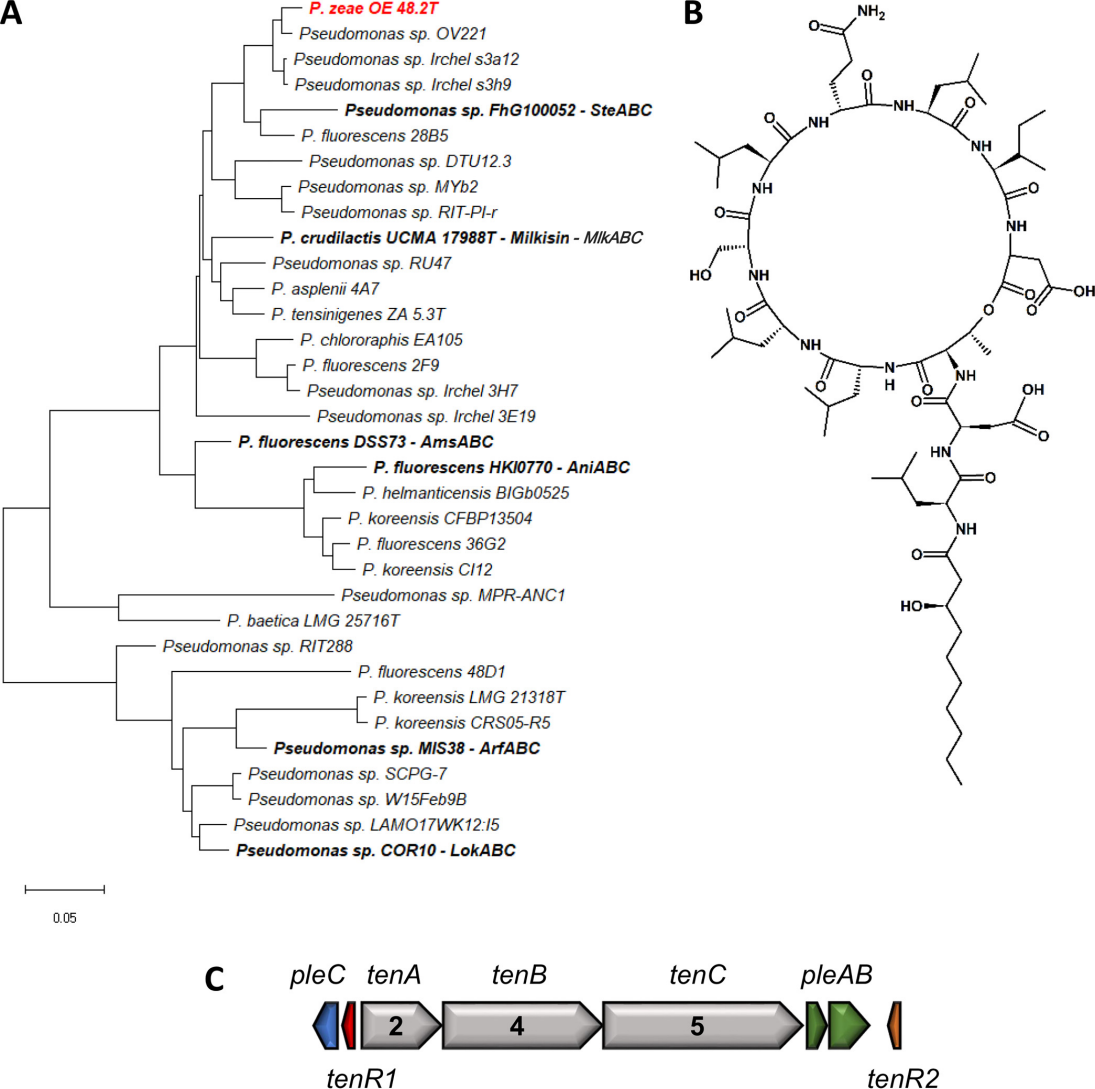
Genetic and chemical characterization of *P. zeae* OE 48.2^T^. (A) Phylogenetic positioning of *P. zeae* OE 48.2^T^ based on concatenated NRPS proteins including known and newly identified members of the Amphisin family. Maximum likelihood tree constructed using the JTT+F+I+G model (MEGA-X). (B) Chemical structure of tensin. (C) Tensin BGC, PleC (QXI14250), 840 TenR1 (QXI14251), TenA (QXI14252), TenB (QXI14253), TenC (QXI14254), PleA (QXI14255), PleB (QXI14256), and TenR2 (QXI14258).

Finally, the lipopeptide extracted from *P. zeae* OE 48.2^T^ features an amino acid sequence composed of 11 amino acids, of which 9 are involved in the macrocycle. Its amino acid sequence is identical to that of tensin and milkisin, which are both members of the Amphisin family. By using a NMR spectral matching approach, as previously used for viscosinamide and pseudodesmin (41), we established that the extracted compound is identical to tensin, thereby also confirming its stereochemistry (Fig. 7B, S15, and S16A; see Table S10 in the supplemental material). In the case of an NMR spectral comparison to milkisin, spectral dissimilarity indicates that both compounds are not identical (Fig. S16B; see Table S11 in the supplemental material).

In the four strains used for chemical validation, the characteristic BGC organization of the respective assigned LP families is conserved (Fig. 1; Fig. 4C to 7C). A split organization is observed only for the xantholysin BGC, with an intergenic distance of 174 kbp between the *xtlA* and *xtlBC* genes of *P. xantholysinigenes* RW9S1A^T^ (Fig. 5C).

### Relationships between *pleB* phylogeny and LP diversity.

The *pleB* genes identified in putative LP producers can be similar or divergent from those of known LP producers (Fig. 3; see Table S12 to S15 in the supplemental material). The *pleB*-based clustering of strains OE 48.2^T^ with FhG100052 among Amphisin family members (Fig. 3) was found to concur with the production of the same lipopeptide tensin, which is consistent with a strong similarity between their NRPS systems (Fig. 7A; Table S15). On the other hand, the presence of a divergent *pleB* gene and distinctive NRPS system within an LP family is not always synonymous with chemical diversity. We demonstrated such discrepancy for *P. xantholysinigenes* RW9S1A^T^ (xantholysin) (Fig. 5A; Table S13) and *P. fulva* LMG 11722^T^ (putisolvin) (Fig. 6A; Table S14) that are producing the same LP as the previously characterized distant family members. This finding contrasts with analysis of the *P. prosekii* LMG 26287^T^ metabolite, which showed that it assembles a new LP, prosekin. Clearly, based on the *pleB* gene (Fig. 3) and concatenated NRPS (Fig. 4A; Table S12) phylogenies, prosekin represents a distinct subgroup within the Bananamide family.

Some of these apparent discrepancies can be linked to the taxonomic position of the producers. Those from the Xantholysin and Putisolvin families are confined to a single, but taxonomically diversified clade, namely, the P. putida group. Bananamide and Amphisin families, hosting much more heterogeneity in terms of chemical structure, are confined typically to isolates belonging to the *P. koreensis* subgroup (7, 39). As observed within the Viscosin family, two WLIP producers (RW10S2 and NSE1) (Fig. 1) are forming a divergent cluster in the *pleB* phylogeny which is in accordance with their taxonomic affiliations to the P. putida group, whereas the other members of the Viscosin family are affiliated with the P. fluorescens group (7, 36). Based on the partial *pleB* gene sequence, *P. prosekii* LMG 26287^T^ clusters with the Viscosin family. However, based on chemical characterization, this strain produces a new LP, prosekin, belonging to the Bananamide family. While all known Bananamide producers belong to the *P. koreensis* subgroup (6), *P. prosekii* LMG 26287^T^ belongs to the *P. mandelii* subgroup (36). It awaits to be discovered whether there are other *P. mandelii* isolates that also produce prosekin-like LPs.

PCR amplification of NRPS gene fragments encoding catalytic domain parts has been used for the detection of NRPS genes in major genera of secondary metabolite producers (28–32). In this study, triggered by the striking correlation we observed between BGC-associated transporter phylogeny and structural classification of BGC products in known LP-producing Pseudomonas strains, we validated an alternative approach based on the *pleB* gene coding for the substrate-capturing ABC transporter component of the dedicated tripartite efflux system required for LP export. This methodology not only allows us to detect LP BGCs but also enables us to assign them to specific LP families. It was successfully applied to a large set of environmental Pseudomonas isolates and shown to generate valuable information about the nature of their produced LP. The methods allows us to prioritize strains of interest for structural and functional characterization combined with genomic sequencing to fully characterize their LP BGC and encoded NRPS system. The potential use of this transporter-guided approach was shown for four type strains, which were not previously recognized as LP producers, by validating the secretion of metabolites (three known ones and a novel one) classified in four different LP families.

Our study has revealed that a comparative analysis of primary sequences of PleB transporters yields valuable information about the chemical nature of the exported pseudomonad LPs. We provide an accessory tool to provisionally assign the putative product of a BGC, revealed by (meta)genomic sequencing, to a particular LP family by comparative analysis against known systems. As it relies on only a small but integral part of a large genomic cluster, such a prediction can even made for BGCs that are incomplete or fragmented, as is frequently observed in draft genomic sequences, in particular for the larger LPs.

It will be of interest to verify whether this transporter-structure correlation extends to other specialized metabolites, for instance between pyoverdines, the major siderophores of Pseudomonas, and their MacB-type exporters. The wider applicability of transporter-informed metabolite characterization is suggested by a comprehensive computational study of BGC-associated transporters highlighting their predictive value for structural classification and general function of a specialized metabolite (27).

## MATERIALS AND METHODS

### BGC identification and PCR primer design.

In this study, we targeted potential producers of LPs from the Bananamide, Amphisin, Gacamide, Putisolvin, Xantholysin, Entolysin, and Tolaasin families. The identification and delineation of LP BGCs in sequenced genomes are facilitated by the fact that BGC organizations and basic NRPS architectural features are conserved within the genus Pseudomonas (Fig. 1). Available BGCs of known producers from these families were retrieved (Table 1), and their NRPS amino acid sequences were used as queries to search for additional strains harboring homologous enzymes in their genomes in GenBank via BLASTP (Table S1). Subsequently, an antiSMASH analysis of genomes from newly identified strains allowed us to confirm the typical features (i.e., the presence of a tandem of TE domains, the absence of separate epimerization domains) and syntenic organization of LP BGCs with NRPS-flanking regulatory and transport genes, family-specific number of modules, and distribution along the NRPS genes (Fig. 1) (42). Finally, the phylogenetic relationship between known and newly identified LP producers was assessed, by family, based on concatenated NRPS sequences (Fig. S2 to S7). The LP *macB*-like sequences (*pleB*) of known producers (Table S1) and retrieved potential producers (Table S2) were used to design two degenerated sets of primers. The design of a single set was not possible as it would have led to an important proportion of degenerated nucleotides. A first set targeting the Bananamide, Amphisin, and Gacamide families was named BanAmpGac F (TGC ACA ACG TCG ARA TBC C) and BanAmpGac R (GCC ATI CGR ATR CCR ATY TC). A second one for the Putisolvin, Xantholysin, Entolysin, and Tolaasin families was named PutXanTol F (YGG CAA RGA RGT SAT GAA BA) and PutXanTol R (TGC ATR ATG CTR TCR AGG TT). These primer sets (BanAmpGac F/R and PutXanTol F/R) lead to the amplification of a fragment of approximatively 1,300 bp and 1,000 bp, respectively (Fig. S8, S9, and S10).

### Amplicon sequencing and phylogenetic analyses.

Bacterial lysates were used as the PCR template as described previously by Girard et al. (36). PCR amplifications of the *pleB* gene were performed using the two primer sets described above (BanAmpGac F/R and PutXanTol F/R) and Kapa2G Fast HotStart ReadyMix (Sigma-Aldrich, St. Louis, MO, USA). Cycling conditions were as follows: initial denaturation at 95°C for 3 min followed by 30 cycles of annealing at 62°C for 15 s, extension at 72°C for 15 s, and denaturation at 95°C for 10 s; and reactions were completed at 72°C for 2 min. PCR products were purified using the GenElute PCR clean-up kit (Sigma-Aldrich). Purified PCR products were sequenced using Sanger sequencing (Macrogen Europe, Amsterdam, The Netherlands). Sequencing was performed using the forward primer PutXanTol F first. In case of failed sequencing, the reverse primer PutXanTol R was used for the first set and both primers BanAmpGac F and BanAmpGac R for the second. An initial phylogenetic tree, based on aligned full-length MacB protein sequences of known LP producers (genetically and/or chemically characterized), was constructed to demonstrate the specificity of the MacB-like protein (PleB) regarding the different LP families and to differentiate it from the paralogue PvdT (Fig. 2 and Fig. S1). The obtained partial *pleB* sequences (Table S3) (*n* = 92) were aligned with the ones of known producers (Table S1) (*n* = 54) and trimmed to make a second tree using a common fragment of approximatively 880 bp (Fig. 3). This fragment corresponds to the nucleotide positions 590 to 1467 of Pseudomonas sp. MIS38 *arfE* (NCBI accession number AB286215.1). MEGA-X software was used to construct both trees, and iTOL was then used to annotate and create high-quality figures (43, 44).

### Chemical characterization of LPs.

Four strains were selected for chemical characterization based on the partial *pleB* phylogeny (Fig. 3), and culture conditions were optimized for the LP production of each strain (detailed in Table S16 in the supplemental material). Two liters of culture was prepared for each strain, and crude extracts were obtained using a previously established protocol (7, 45).

The LP-enriched precipitate was dissolved in methanol. Purification conditions were optimized by using an Agilent Technologies 1100 Series high-pressure liquid chromatography (HPLC) device equipped with a Luna C_18_ analytical reversed-phase HPLC column (250 by 4.6 mm; 5 μm). The signal was detected using a diode array detector at a wavelength of 214 nm. Purification of the LPs was then performed by injection of the methanol solution into a Prostar HPLC device (Agilent Technologies) equipped with a Luna C_18:2_ preparative RP-HPLC column (250 by 21.2 mm; 5-μm particle size) for separation of the individual LP analogues. For each individual LP, an optimized elution gradient of H_2_O/CH_3_CN was applied at a flow rate of 17.5 mL min^−1^, while the column temperature was kept at 35°C.

All NMR measurements were performed on either a Bruker Avance III spectrometer operating at a respective ^1^H and ^13^C frequency of 500.13 MHz and 125.76 MHz and equipped with a BBI-Z probe or an Bruker Avance II spectrometer operating at a ^1^H and ^13^C frequency of 700.13 MHz and 176.05 MHz, respectively, and equipped with a 5-mm Prodigy TCI probe. The sample temperature was set to 298.0 K or 323 K, as indicated. Standard pulse sequences as present in the Bruker library were used throughout. High-precision 5-mm NMR tubes (Norell, Landisville, NJ) were used. Dimethylformamide-d7 (DMF) (99.50%) or acetone-d6 was used as a solvent throughout and was purchased from Eurisotop (Saint-Aubin, France). ^1^H and ^13^C chemical shift scales were calibrated by using the residual solvent signal using trimethylsilyl (TMS) as a secondary reference. For NMR spectral matching, the recorded position of the CH^α^ resonances in the ^1^H-^13^C HSQC spectra are compared with the published chemical shift values of tensin (10) and milkisin (46), recorded under identical conditions.

The 2D spectra measured for structure elucidation include a 2D ^1^H-^1^H DQF-COSY, 2D ^1^H-^1^H TOCSY with a 90-ms MLEV-17 spinlock, 2D ^1^H-^1^H NOESYs with various mixing times, 2D ^1^H-^1^H off-resonance ROESYs with a mixing time of 200 ms, and gradient-selected ^1^H-{^13^C} gHSQC and gHMBC. Typically, 2,048 data points were sampled in the direct dimension for 512 data points in the indirect dimension, with the spectral width set to 11 ppm and 110 ppm along the ^1^H and ^13^C dimension, respectively. The ^1^H-^13^C HMBC was measured with a 210-ppm ^13^C spectral width. For 2D processing, the spectra were zero filled to a 2,048 by 2,048 real data matrix. Before Fourier transformation, all spectra were multiplied with a squared cosine bell function in both dimensions or sine bell in the direct dimension for the gHMBC; the latter was done prior to magnitude calculation.

### Data availability.

Accession numbers for new nucleotide sequences are included in Table S1. Whole-genome sequences (WGS) for each species as follows are in GenBank: Pseudomonas carnis DR54 (JAFLXE000000000), Pseudomonas sp. Wu6 (JAFLXH000000000), and P. fluorescens DSS73 (JAFLXG000000000). Information for the partial *pleB* amplicon sequences are in Table S4.
