# Supplementary material for: Transporter Gene-Mediated Typing for Detection and Genome Mining of Lipopeptide-Producing Pseudomonas
Source: Appl Environ Microbiol. 2022 Jan 25;88(2):e01869-21. doi: 10.1128/AEM.01869-21 (PMC8788793; doi:10.1128/AEM.01869-21)
Supplement: Supplemental file 1 — Tables S1 to S16, Fig. S1 to S16. Download AEM.01869-21-s0001.pdf, PDF file, 2.1 MB [file aem.01869-21-s0001.pdf]

Table S1. Genbank accession numbers for NRPS genes, PleB and PvdT homologs of genetically and/or chemically characterized LP producers. Strains used for primer validation are highlighted in bold. NA: not available.

LP family	LP	Species	Strain	Genbank Accession numbers		
				NRPS genes	PleB	PvdT homologs
**Factin**	Cichofactin	*P. cichorii*	SF1-54	KJ513093	AHZ34236	NA
	Cichofactin	*P. cichorii*	JBC1	CP007039	AHF67761	AHF68266
	Syringafactin	*P. syringae* pv. *tomato*	DC3000	NC_004578	AAO56331	AAO55676
	Virginiafactin	*Pseudomonas* sp.	QS1027	PHSU01000004	-	WP_100942482
**Bananamide**	MDN-0066	*P. granadensis*	**LMG 27940^{T}**	LT629778	SDT21849	SDS76671
	Bananamide I-III (A-C)	*P. bananamidigenes*	**BW11P2^{T}**	KX437753	AOA33125	WP_065257421
	Bananamide D-G	*P. botevensis*	**COW3^{T}**	MN480426	QGQ63522	NA
**Viscosin**	Viscosin	*P. fluorescens*	BBc6R8	AKXH02000046	ESW57247	ESW57047
	Viscosin	*P. fluorescens*	SBW25	AM181176	CAY48791	CAY50378
	Viscosinamide	*P. carnis*	DR54	JAFLXE010000121	MBY8955620	MBY8953970
	Viscosinamide	*Pseudomonas* sp.	A2W4.9	MT771985	QLY89349	NA
	Viscosinamide	*Pseudomonas* sp.	U2W1.5	MT749673	QLY89271	NA
	WLIP	*P. chlororaphis* subsp. *aurantiaca*	PB-St2	CP027716	AZD30265	AZD28700
	WLIP	*P. wayambapalatensis*	RW10S2	JN982332; JN982333	AFJ23828	MBC3466575
	WLIP	*Pseudomonas* sp.	NSE1	MK650230	QDC17528	NA
	WLIP	*P. fluorescens*	LMG 5329	JQ974026; ASGY01000102	AFZ61518	KGE66557
	Massetolide	*P. lactis*	SS101	EU199081; AHPN01000001	ABW87988	EIK59118
	Pseudodesmin	*Pseudomonas* sp.	COR52	MT577358	QLY89266	NA
	Pseudodesmin	*P. tolaasii*	NCPPB 2192^{T} = LMG 2342^{T}	PHHD01000001	PKA78224	PKA76802
	Pseudophomin	*Pseudomonas* sp.	BRG-100	JPRX01000000	KFF47238	KFF42812
**Orfamide**	Orfamide	*P. protegens*	CHA0	CP003190	AGL83961	AGL85905
	Orfamide	*P. protegens*	Pf-5	CP000076	Q4KES7	Q4K9A4
	Orfamide	*P. aestus*	CMR5c	KT613918; CP027705	AZC17624	AZC19679
	Orfamide	*P. sessilinigenes*	CMR12a^{T}	JQ309921; CP077074	AZC23861	AZC27651
**Poaeamide**	Poaeamide A	*P. poae*	RE*1-1-14	CP004045	AGE25482	AGE26451
	PPZPM-1a	*Pseudomonas* sp.	Wu6	JAFLXH010000004	MBY8928182	MBY8929322

Table S1. Continued.

**Amphisin**	Amphisin	*P. fluorescens*	**DSS73**	JAFLXG010000010	MBY8934714	MBY8938039
	Anikasin	*P. fluorescens*	HKI0770	LVEJ01000013	WP_064118557	WP_064116851
	Arthrofactin	*Pseudomonas* sp.	**MIS38**	AB107223; JAFLXF010000011	BAF40423	MBY8957176
	Lokisin	*Pseudomonas* sp.	**COR10**	MK534107	QDF82252	NA
	Milkisin	*P. crudilactis*	**UCMA 17988^T**	WXVV01000014	WP_175554031	WP_016983522
	Tensin/Stechlisin	*Pseudomonas* sp.	FhG100052	MT080808	QNL34620	NA
**Gacamide**	Gacamide	*P. fluorescens*	**Pf0-1**	CP000094	ABA73958	ABA73599
	Cocoyamide	*Pseudomonas* sp.	**COW5**	-	-	-
**Putisolvin**	Putisolvin	*P. putida*	PCL1445	DQ151887	ABW17379	NA
	Putisolvin	*P. capeferrum*	**WCS358**	JMIT01000001	KEY88314	KEY89533
	Putisolvin	*Pseudomonas* sp.	**COR19**	MT511055	QMN69946	NA
	Putisolvin	*P. vlassakiae*	**WCU_60**	MT511056	QMN69955	NA
	Putisolvin	*P. vlassakiae*	**WCU_64**	MT511054	QMN69936	NA
**Xantholysin**	Xantholysin	*P. mosselii*	**BW11M1**	KC297506; LSLE01000008	AGM14936	KXG80973
	Xantholysin	*P. soli*	**LMG 27941^T**	FOEQ01000011	SER70164	SEQ30648
	Xantholysin	*Pseudomonas* sp.	250J	JHEE01000093	KNX77880	KNX78771
**Entolysin**	Entolysin	*P. entomophila*	**L48**	CT573326	CAK15812	CAK16390
**Tolaasin**	Tolaasin	*P. tolaasii*	**NCPPB 2192^T = LMG 2342^T**	PHHD01000001	PKA77400	PKA76802
	Tolaasin F	*P. costantinii*	**DSM 16734^T = LMG 22119^T**	HE967327; FNTS00000000	CCJ67642	NA
	Sessilin	*P. sessilinigenes*	**CMR12a^T**	JQ309920; CP077074	AZC26608	AZC27651
**Peptin**	Syringopeptin SP22	*P. syringae* pv. *syringae*	B301D	CP005969	AKF46139	AKF45451
	Syringopeptin SP25	*P. syringae* pv. *atrofaciens*	NCPPB 2612^T = LMG 5095^T	CP028490	AVX22970	AVX23587
	Cichopeptin	*P. cichorii*	SF1-54	KJ513094	AHZ34249	NA
	Fuscopeptin	*P. fuscovaginae*	LMG 2158^T	LT629972	SEI20481	SEI15793
	Jessenipeptin	*Pseudomonas* sp.	QS1027	PHSU01000014	WP_100940991	WP_100942482
	Nunapeptin	*P. fluorescens*	In5	KC880158	AHL29295	KPN87467
	Corpeptin	*P. corrugata*	NCPPB 2445^T = LMG 2172^T	LT629798	SDU91482	NA
	Thanapeptin	*Pseudomonas* sp.	SH-C52	CBLV010000330	CDF96570	NA
	Sclerosin	*P. brassicacearum*	DF41	CP007410	AHL34330	AHL35128

Table S2. Putative LP-producing *Pseudomonas* strains tentatively assigned to the Bananamide, Amphisin, Gacamide, Putisolvin, Xantholysin and Tolaasin families and used for primer design. Strains used for primer validation are highlighted in bold.

LP Family	Species	Strain	NRPS genes	PleB	References
**Bananamide**	*P. azadiae*	SWRI103^T	JABBCM010000002	WP_169374389	7
	P. fluorescens	SF4c	JTGH01000016	WP_039770766	1
	P. moraviensis	Devor	MAYQ01000007	WP_065617028	2
		**BS3668 = LMG 24280^T**	LT629788	SDU45537	3,4
	P. putida	NFIX47	FMXW01000001	WP_074687878	-
	Pseudomonas sp.	B1(2017)	NEJQ01000015	WP_085613089	5
		B17(2017)	NEJT01000009	WP_085686556	5
		B25(2017)	NEJK01000009	WP_085648946	5
		B30(2017)	NEJE01000006	WP_085667792	5
		B31(2017)	NEJD01000017	WP_085609001	5
		DR 5-09	CP011566	ANI54108	4
		FW104-15G4B	PPSA01000017	POA35870	-
		MS82	CP028826	AWA42427	6
		MS586	CP014205.2	AMQ85073	4
		NDM	QETJ01000011	WP_065617028	-
		R15(2017)	NEII01000019	WP_064382265	5
		R45(2017)	NEHH01000015	WP_085748378	5
		Z003-0.4C(8344-21)	LT629756	SDS91038	5
**Amphisin**	*P. asplenii*	4A7	LUUC01000001	WP_102899160	-
	P. baetica	**DSM 26532^T = LMG 25716^T**	PHHE01000001	WP_100848244	8, 9
	P. chlororaphis	EA105	JSFK01000023	WP_038365929	10, 11
	P. fluorescens	2F9	MOBY01000032	WP_123376752	-
		28B5	MOBX01000013	WP_123450381	12
		36G2	MOBZ01000013	WP_123594340	13
		48D1	MOBT01000001	RON67091	13
	P. helmanticensis	BIGb0525	SOCQ01000025	WP_134178127	-
	P. koreensis	**BS3658 = LMG 21318^T**	LT629687	SDE55014	14, 3
		CFBP 13504	QFZV01000001	WP_123594340	15
		CI12	MPLD01000005	WP_077572428	16
		CRS05-R5	CP015852	ANH97970	17
	Pseudomonas sp.	DTU12.3	CP027218	QAX85497	18
		Irchel s3a12	FYDN01000001	WP_095124694	-
		Irchel s3h9	FYDH01000003	WP_095047883	-
		Irchel 3E19	FYDT01000029	WP_095190156	-
		Irchel 3H7	FYEA01000002	WP_007915988	-
		LAMO17WK12:I5	OBDS01000007	WP_097087890	-
		MPR-ANC1	PPRY01000004	WP_103303538	-
		MYb2	PCQC01000009	PRC32977	19
		OV221	QUMR01000003	WP_116254885	-
		RIT288	JFYN01000013	WP_047599789	20
		RIT-PI-r	LIGE01000025	WP_064390457	21

Table S2. Continued.

		RU47	CP022411	AZZ76844	22
		SCPG-7	MVOO01000005	WP_041067192	23
		W15Feb9B	JTKF01000013	WP_041067192	24
**Gacamide**	*P. fluorescens*	CH267	NXNJ01000008	WP_096820567	25
	P. kribbensis	CHA-19	SPDQ01000017	TFH79438	26
		46-2^T = KCTC 32541^T	CP029608	AXI61119	27
	Pseudomonas sp.	FW215-E1	POFQ01000005	WP_102622126	-
		R22(2017)	NEIC01000001	WP_085708568	5
		R40(2017)	NEHL01000009	WP_085733259	5
		91RF	QWFC01000013	RIJ12893	13
**Putisolvin**	*P. fulva*	FDAARGOS_167	CP014025	AVF55546	-
		YAB-1	LAWW01000007	WP_049695975	28
	P. parafulva	**NBRC 16636^T = LMG 24038^T**	BBIU01000002	WP_161629589	29
		NS96	LDSN01000042	WP_058639246	30
		NS212	LDSM01000022	WP_058604554	30
		PRS09-11288	CP019952	AQW68749	-
	P. putida	E41	CP024085	ATP44930	31
		E46	CP024086	ATP50402	31
	P. reidholzensis	CCOS 865^T = LMG 29328^T	UNOZ01000027	WP_119143123	32,33
	Pseudomonas sp.	R9(2017)	NEHD01000020	WP_085679527	5
		URM017WK12:I11	LN854573	CRN08391	-
**Xantholysin**	*P. mosselii*	BS011	CP023299	ATB67649	34
		Gil3	LZCV00000000	WP_069016638	35
		PtA1	CP024159	WP_069016638	36
	P. putida	1A00316	CP014343	AMK31368	37
	P. soli	SJ10	CP009365	AIN58327	38
	Pseudomonas sp.	CCOS 191	LN847264	CRI56941	39
		PONIH3	CP026386	AUY32235	40
**Tolaasin**	*P. palleroniana*	**BS3265 = LMG 23076^T**	FNUA01000002	SEF10758	3
		MAB3	CP025494	AVE03135	41
		Ps006	LRMR01000034	WP_060756406	42
	Pseudomonas sp.	BIOMIG1BAC	CP049045	QIH08074	43
		MWU12-2534b	PQCH02000001	RBJ85735	44
		TK014	QKLO01000020	WP_016964928	-

Table S3. Results of the amplifications with the BanAmpGac and PutXanTol primer sets on a selection of known producers belonging to non-targeted LP families.

LP family	LP	Species	Strain	BanAmpGac F/R	PutXanTol F/R	References
Factin	Syringafactin	*P. syringae* pv. *tomato*	DC3000	+	-	45
Viscosin	Viscosin	*P. fluorescens*	SBW25	+	+	46
	Viscosinamide	*P. carnis*	DR54	+	-	47
	WLIP	*P. wayambapalatensis*	RW10S2^T	+	-	48
		P. fluorescens	LMG 5329	+	-	49
Orfamide	Orfamide	*P. protegens*	CHA0	-	+	50, 51
			Pf-5	-	+	52
		P. aestus	CMR5c	-	-	51
		P. sessilinigenes	CMR12a^T	+	+	53
Poaeamide	PPZPM-1a	*Pseudomonas* sp.	Wu6	+	-	54

Table S4. *Pseudomonas* strains used in this study. The results of the amplifications with the BanAmpGac (BAG) and PutXanTol (PXT) primer sets and the accession numbers of their partial *pleB* sequences are indicated. NA indicates strains for which no amplification was observed.

LP Family	Species	Strain	Origin	BAG	PXT	Accession nr partial *pleB*	Accession Number NRPSs (1; 2; 3)	Accession Number Genomic region/Genome	Chemical characterization	References
**Bananamide**	*P. azadiae*	SWRI103^T	Wheat rhizosphere, Shiraz, Iran	+	+	MW509424	MBV4451460; MBV4451459; MBV4451458	JAHSTY010000001	-	-
	P. hamedanensis	SWRI65^T	Wheat rhizosphere, Hamedan, Iran	+	+	MW509421	QXI18666; QXI18665; QXI18664	CP077091	-	-
	P. prosekii	LMG 26867^T	Soil, Antarctica	+	-	MW509420	WP_092279781; WP_092279783; WP_092279785	LT629762	Prosekin	This study
	P. hamedanensis	SWRI81	Wheat rhizosphere, Hamedan, Iran	+	+	MW509422	WP_186621444; WP_186621443; WP_189684257	JABWQS010000021	-	-
	P. hamedanensis	SWRI99	Wheat rhizosphere, Kermanshah, Iran	+	+	MW509423	WP_186649071; WP_186649070; WP_189684881	JABWQR010000014	-	-
	P. hamedanensis	SWRI111	Wheat rhizosphere, Kermanshah, Iran	+	+	MW509425	WP_186632740; WP_186632737; WP_189664035	JABWQQ010000008	-	-
	Pseudomonas sp.	B1M3-32	White cocoyam rhizosphere, Boteva, Cameroon	+	-	MW509426	-	-	Bananamide D-G	55
		B1M1-1	White cocoyam rhizosphere, Boteva, Cameroon	+	-	MW509427	-	-	Bananamide D-G	55
		B2M1-30	White cocoyam rhizosphere, Boteva, Cameroon	+	-	MW509428	-	-	Bananamide D-G	55
		B1M1-2	White cocoyam rhizosphere, Boteva, Cameroon	+	-	MW509429	-	-	Bananamide D-G	55
		BW7P1	Rhizoplane banana, Galagadera, Sri Lanka	+	-	MW509451	-	-	-	-
**Viscosin**	*P. carnis*	SWRI22	Wheat rhizosphere, Kermanshah, Iran	+	-	MW509438	MBV4512834; MBV4511467; MBV4511468	JABWQK020000002; JABWQK020000001	-	-
	P. chlororaphis subsp. *aurantiaca*	LMG 21630^T	-	+	+	MW509430	WP_081360707; WP_081360708; WP_124344771	CP027746	-	-
	P. lurida	PGSB3962	Maize rhizosphere, Belgium	+	-	MW509431	WP_186761964; WP_186762532; WP_189661046	JABWQC010000005; JABWQC010000018	-	-

		PGSB7828	Maize rhizosphere, Mons, Belgium	+	-	MW509432	WP_186605378; WP_186605699; WP_189655651	JABWQD010000009; JABWQD010000014	-	-
		PGSB8273	Maize rhizosphere, Mons, Belgium	+	-	MW509433	WP_186574130; WP_186574058; WP_189663451	JABWQE010000012; JABWQE010000011	-	-
	P. paralactis	SWRI70	Wheat rhizosphere, Shiraz, Iran	+	-	MW509434	WP_186560891; WP_186558945; WP_189663542	JABWQI010000015; JABWQI010000002	-	-
	P. wayambapalatensis	RW3S1^T	Exorhizosphere rice, Kurunegala, Sri Lanka	+	-	MW509436	QXI41498; QXI41430; QXI41429	CP077096	-	-
	P. wayambapalatensis	RW3S2	Exorhizosphere rice, Kurunegala, Sri Lanka	+	-	MW509437	MBC3419664; MBC3419733; MBC3419734	JABWRM010000002	-	-
	P. yamanorum	LMG 27247^T	Soil, Ushuaia, Argentina	+	+	MW509435	WP_093208195; WP_093208198; WP_093208200	LT629793	-	-
	P. salmasensis	SWRI126^T	Wheat rhizosphere, Salmas, Iran	+	-	MW509439	WP_186607714; WP_186608257; WP_189661240	JABWQJ010000013; JABWQJ010000022	-	-
**Orfamide**	*Pseudomonas* sp.	BW16M1	Endorhizosphere banana, Peradeniya, Sri Lanka	+	-	MW509441	-	-	-	-
**Poaeamide**	*P. poae*	LMG 21465^T	Grasses phyllosphere, Germany	+	-	MW509442	WP_060549360; WP_060548940; WP_060548941	LT629706	-	-
		SWRI2	Wheat rhizosphere, West Azerbaijan, Iran	+	-	MW509443	WP_186751541; WP_186751804; WP_186751803	JABWQH010000022	-	-
**Amphisin**	*P. monsensis*	PGSB 8459^T	Maize rhizosphere, Belgium	+	-	MW509448	-	-	-	-
	P. tensinigenes	ZA 5.3^T	Wheat rhizosphere, Belgium	+	-	MW509449	QXI03914; QXI03913; QXI03912	CP077089	-	-
	P. zeae	OE 48.2^T	Maize rhizosphere, Belgium	+	-	MW509447	QXI14252; QXI14253; QXI14254	CP077090	Tensin	This study
**Gacamide**	*Pseudomonas* sp.	B1M2-19	White cocoyam rhizosphere, Boteva, Cameroon	+	-	MW509450	-	-	Cocoyamide	55
**Putisolvin**	*P. capeferrum*	SWRI59	Wheat rhizosphere, Salmas, Iran	+	+	MW509475	MBC3502492; MBC3502493; MBC3502494	JABWRT010000010	-	-
		SWRI68	Wheat rhizosphere, Shiraz, Iran	+	+	MW509477	MBC3508413; MBC3508412; MBC3508411	JABWRU010000011	-	-
		SWRI77	Wheat rhizosphere, Zanjan, Iran	-	+	MW509478	-	-	-	-

P. fulva	LMG 11722[T]	Rice paddy, Japan	-	+	MW509452	WP_033737094; WP_042139413; WP_042139412	BBIQ01000007	Putisolvin III-V	This study
P. kermanshahensis	SWRI50	Wheat rhizosphere, Zanjan, Iran	+	+	MW509474	MBC3484797; MBC3484798; MBC3484799	JABWRW010000002	-	-
	SWRI67	Wheat rhizosphere, Hamedan, Iran	+	+	MW509476	MBC3495245; MBC3495244; MBC3495243	JABWRX010000003	-	-
	SWRI100[T]	Wheat rhizosphere, Kermanshah, Iran	-	+	MW509479	MBV4527003; MBV4527002; MBV4527001	JABWRY020000001	-	-
P. vlassakiae	RW4S2[T]	Exorhizosphere rice, Kurunegala, Sri Lanka	+	+	MW509473	-	-	-	-
Pseudomonas sp.	COR30	Red cocoyam rhizosphere, Ekona, Cameroon	+	+	MW509454	-	-	Putisolvin	56
	COR55	Red cocoyam rhizosphere, Ekona, Cameroon	-	+	MW509455	-	-	Putisolvin III-V	56
	COW17	White cocoyam rhizosphere, Maumu, Cameroon	+	+	MW509456	-	-	Putisolvin	56
	COW18	White cocoyam rhizosphere, Maumu, Cameroon	-	+	MW509457	-	-	Putisolvin	56
	COW22	White cocoyam rhizosphere, Maumu, Cameroon	-	+	MW509458	-	-	Putisolvin	56
	COW23	White cocoyam rhizosphere, Maumu, Cameroon	-	+	MW509459	-	-	Putisolvin	56
	COW29	White cocoyam rhizosphere, Maumu, Cameroon	-	+	MW509460	-	-	Putisolvin	56
	COW32	White cocoyam rhizosphere, Maumu, Cameroon	-	+	MW509461	-	-	Putisolvin	56
	COW73	White cocoyam rhizosphere, Boteva, Cameroon	+	+	MW509462	-	-	Putisolvin	56
	NNC3	Red cocoyam, Ugbokolo, Nigeria	-	+	MW509463	-	-	Putisolvin	56
	NNC5b	Red cocoyam, Ugbokolo, Nigeria	-	+	MW509464	-	-	Putisolvin	56
	RCA_48	Red cocoyam, Ado-Ekiti, Nigeria	-	+	MW509465	-	-	Putisolvin	56
	RCA_49	Red cocoyam, Ado-Ekiti, Nigeria	-	+	MW509466	-	-	Putisolvin	56
	RCA_50	Red cocoyam, Ado-Ekiti, Nigeria	-	+	MW509467	-	-	Putisolvin	56
	RCA_51	Red cocoyam, Ado-Ekiti, Nigeria	-	+	MW509468	-	-	Putisolvin	56
	RCA_53	Red cocoyam, Ado-Ekiti, Nigeria	-	+	MW509469	-	-	Putisolvin	56
	RCA_54	Red cocoyam, Ado-Ekiti, Nigeria	-	+	MW509470	-	-	Putisolvin	56
	RCA_55	Red cocoyam, Ado-Ekiti, Nigeria	-	+	MW509471	-	-	Putisolvin	56
	RW4P3	Rhizoplane rice, Kurunegala, Sri Lanka	+	+	MW509472	-	-	-	-

		WCU_61	White cocoyam rhizosphere, Umudike, Nigeria	-	+	MW509480	-	-	Putisolvin	56
		WCU_62	White cocoyam rhizosphere, Umudike, Nigeria	-	+	MW509481	-	-	Putisolvin	56
		WCU_63	White cocoyam rhizosphere, Umudike, Nigeria	-	+	MW509482	-	-	Putisolvin	56
		WCU_65	White cocoyam rhizosphere, Umudike, Nigeria	-	+	MW509483	-	-	Putisolvin	56
		WCU_66	White cocoyam rhizosphere, Umudike, Nigeria	-	+	MW509484	-	-	Putisolvin	56
		WCU_68	White cocoyam rhizosphere, Umudike, Nigeria	-	+	MW509485	-	-	Putisolvin	56
**Xantholysin**	*P. maumuensis*	COW77^T	White cocoyam rhizosphere, Maumu, Cameroon	+	+	MW509509	QXH58577; QXH58858; QXH58859	CP077077	Xantholysin	57
	P. mosselii	BW18S1	Banana plant rhizosphere, Colombo, Sri Lanka	+	+	MW509486	MBC3450292; MBC3451499; MBC3451498	JABWRK010000003; JABWRK010000007	-	-
		RW5S2	Exorhizosphere rice, Kurunegala, Sri Lanka	+	+	MW509487	-	-	-	-
	P. muyukensis	COW39^T	White cocoyam rhizosphere, Ekona, Cameroon	+	+	MW509500	QXH33350; QXH33344; QXH33345	CP077073	Xantholysin	57
	P. peradeniyensis	BW13M1^T	Banana plant endorhizosphere, Peradeniya, Sri Lanka	+	+	MW509488	MBV4504140; MBV4504224; MBV4504225	JABWRJ020000001	-	-
	P. peradeniyensis	BW16M2	Banana plant endorhizosphere, Peradeniya, Sri Lanka	-	+	MW509489	MBC3436578; MBC3436495; MBC3436494	JABWRI010000013	-	-
	P. xantholysinigenes	RW9S1A^T	Exorhizosphere rice, Kurunegala, Sri Lanka	+	+	MW509510	QXI40745; QXI36232; QXI36231	CP077095	Xantholysin A	This study
	Pseudomonas sp.	COR21	Red cocoyam rhizosphere, Maumu, Cameroon	-	+	MW509490	-	-	Xantholysin	57
		COR22	Red cocoyam rhizosphere, Ekona, Cameroon	+	+	MW509491	-	-	Xantholysin	57
		COR23	Red cocoyam rhizosphere, Ekona, Cameroon	+	+	MW509492	-	-	Xantholysin	57
		COR42	Red cocoyam rhizosphere, Boteva, Cameroon	+	+	MW509493	-	-	Xantholysin	57
		COR49	Red cocoyam rhizosphere, Boteva, Cameroon	-	+	MW509494	-	-	Xantholysin	57
		COR51	Red cocoyam rhizosphere, Ekona, Cameroon	+	+	MW509495	-	-	Xantholysin	57
		COR56	Red cocoyam rhizosphere, Boteva, Cameroon	+	+	MW509496	-	-	Xantholysin	57

		COR59	Red cocoyam rhizosphere, Boteva, Cameroon	+	+	MW509497	-	-	Xantholysin	57
		COW37	Red cocoyam rhizosphere, Boteva, Cameroon	-	+	MW509498	-	-	Xantholysin	57
		COW38	White cocoyam rhizosphere, Ekona, Cameroon	+	+	MW509499	-	-	Xantholysin	57
		COW42	White cocoyam rhizosphere, Ekona, Cameroon	+	+	MW509501	-	-	Xantholysin	57
		COW43	White cocoyam rhizosphere, Ekona, Cameroon	+	+	MW509502	-	-	Xantholysin	57
		COW53	White cocoyam rhizosphere, Boteva, Cameroon	+	+	MW509503	-	-	Xantholysin	57
		COW63	White cocoyam rhizosphere, Boteva, Cameroon	-	+	MW509504	-	-	Xantholysin	57
		COW68	White cocoyam rhizosphere, Maumu, Cameroon	-	+	MW509505	-	-	Xantholysin	57
		COW72	White cocoyam rhizosphere, Boteva, Cameroon	+	+	MW509506	-	-	Xantholysin	57
		COW75	White cocoyam rhizosphere, Boteva, Cameroon	-	+	MW509507	-	-	Xantholysin	57
		COW76	White cocoyam rhizosphere, Boteva, Cameroon	-	+	MW509508	-	-	Xantholysin	57
**Tolaasin**	*P. tolaasii*	CH36	*Agaricus bisporus*, Belgium	-	+	MW509512	-	-	-	-
		LMG 2345	*Agaricus bisporus*, Italy	-	+	MW509513	-	-	-	-
		LMG 2346	*Agaricus bisporus*, New Zealand	-	+	MW509514	-	-	-	-
		LMG 6641	*Agaricus bisporus*, Belgium	-	+	MW509515	-	-	-	-
**NA**	*P. edaphica*	OE 28.3	Wheat rhizosphere, West-Vlaanderen, Belgium	-	-	-	QXI57341.1; QXI61632; QXI61633	CP077082		
	P. fakonensis	COW40^T	White cocoyam rhizosphere, Ekona, Cameroon	-	-	-	QXH53928; QXH53929; QXH53930	CP077076	WLIP	57
	P. oryzicola	RD9SR1^T	Exorhizosphere rice, Anuradhapura, Sri Lanka	-	-	-	-	No LP BGC: JABWRZ000000000		
	P. taiwanensis	RW2S1	Exorhizosphere rice, Kurunegala, Sri Lanka	-	-	-	-	No LP BGC: JABWRR000000000		
	P. tolaasii	LMG 6654	*Pleurotus ostreatus*, Belgium	-	-	-	-	-	-	-
	P. xanthosomae	COR54^T	Red cocoyam rhizosphere, Ekona, Cameroon	-	-	-	QXH44673; QXH44672; QXH44671	CP077075	WLIP	57
	P. vlassakiae	NNC7	Red cocoyam, Ugbokolo, Nigeria	-	-	-	-	-	Putisolvin III-V	56
	Pseudomonas sp.	BW7M1	Endorhizosphere banana, Galagadera, Sri Lanka	-	-	-	-	-		
		BW7M2	Endorhizosphere banana, Galagadera, Sri Lanka	-	-	-	-	-		

BW7P2	Rhizoplane banana, Galagadera, Sri Lanka	-	-	-	-	-		
BW7S2	Exorhizosphere banana, Galagadera, Sri Lanka	-	-	-	-	-		
BW8S1	Exorhizosphere banana, Galagadera, Sri Lanka	-	-	-	-	-		
BW11P1	Rhizosphere banana, Galagadera, Sri Lanka	-	-	-	-	-		
BW12M1	Endorhizosphere banana, Peradeniya, Sri Lanka	-	-	-	-	-		
BW12M2	Endorhizosphere banana, Peradeniya, Sri Lanka	-	-	-	-	-		
BW12P1	Rhizoplane banana, Peradeniya, Sri Lanka	-	-	-	-	-		
BW12S1	Banana rhizosphere, Peradeniya, Sri Lanka	-	-	-	-	-		
BW12S2	Exorhizosphere banana, Peradeniya, Sri Lanka	-	-	-	-	-		
BW13S2	Exorhizosphere banana, Peradeniya, Sri Lanka	-	-	-	-	-		
BW14S1	Exorhizosphere banana, Peradeniya, Sri Lanka	-	-	-	-	-		
BW14S2	Exorhizosphere banana, Peradeniya, Sri Lanka	-	-	-	-	-		
BW15P1	Banana rhizosphere, Peradeniya, Sri Lanka	-	-	-	-	-		
BW15S1	Exorhizosphere banana, Peradeniya, Sri Lanka	-	-	-	-	-		
BW16S1	Exorhizosphere banana, Peradeniya, Sri Lanka	-	-	-	-	-		
BW17S1	Exorhizosphere banana, Colombo, Sri Lanka	-	-	-	-	-		
COW62	White cocoyam rhizosphere, Maumu, Cameroon	-	-	-	-	-	Putisolvin	57
RW1P1	Rhizoplane rice, Kurunegala, Sri Lanka	-	-	-	-	-		
RW2P1	Rhizoplane rice, Kurunegala, Sri Lanka	-	-	-	-	-		
RW2P3	Rhizoplane rice, Kurunegala, Sri Lanka	-	-	-	-	-		
RW8P3	Rhizoplane rice, Kurunegala, Sri Lanka	-	-	-	-	No LP BGC: JABWRC000000000		
SWRI101	Wheat rhizosphere, Kermanshah, Iran	-	-	-	-	-		
SWRI80	Wheat rhizosphere, Hamedan, Iran	-	-	-	-	-		
WCA_17	White cocoyam, Ado-Ekiti, Nigeria	-	-	-	-	-	Xantholysin	56
WCA_13	White cocoyam, Ado-Ekiti, Nigeria	-	-	-	-	-	Xantholysin	56

Table S5. ^{1}H and ^{13}C chemical shift values of prosekin from the medium extract of *P. prosekii* LMG 26867^T with retention time 11.9 minutes (700 MHz, DMF-d7, 298 K).

		^{1}H δ [ppm]	^{13}C δ [ppm]			^{1}H δ [ppm]	^{13}C δ [ppm]
**HDA**				**Leu4**			
	CO	-	174,04		NH	7,38	-
	$CH_2α$	2,45	43,7		CHα	4,18	54,12
	CHβ	4,04	68,96		CO	-	174,9
	$CH_2γ$	1,5	37,49		$CH_2β1$	1,79	40,43
	$CH_2δ1$	1,46	25,38		$CH_2β2$	1,67	40,43
	$CH_2δ2$	1,36	25,37		CHγ	1,7	24,48
	CH_2 ε	1,29	29,36		$CH_3δ1$	1,02	21,86
	CH_2 ζ	1,27	31,8		$CH_3δ2$	0,92	21,11
	CH_2 η	1,29	22,51	**Leu5**			
	CH_3 θ	0,88	13,69		NH	9,08	-
	OH	n.d.	-		CHα	4,11	53,53
					CO	-	173
**Leu1**					$CH_2β$	1,76	39,31
	NH	8,58	-		CHγ	1,89	24,67
	CHα	4,22	53,49		$CH_3δ1$	0,9	20,09
	CO	-	172,26		$CH_3δ2$	0,97	22,93
	$CH_2β1$	1,67	40,07				
	$CH_2β2$	1,6	40,05	**Ser6**			
	CHγ	1,83	24,67		NH	8,22	-
	$CH_3δ1$	0,94	22,67		CHα	4,48	58,04
	$CH_3δ2$	0,92	21,11		CO	-	169,76
					$CH_2β1$	4,06	61,07
**Glu2**					$CH_2β2$	3,98	61,08
	NH	8,14	-		OH	n.d.	-
	CHα	4,22	53,89				
	CO	-	172,26	**Ile7**			
	$CH_2β1$	2,13	26,12		NH	7,48	-
	$CH_2β2$	1,92	26,12		CHα	4,35	58,78
	$CH_2γ$	2,41	30,35		CO	-	170,63
	COδ	-	174,26		CHβ	1,78	38,5
	OH	n.d.	-		$CH_2γ1$	1,56	24,69
					$CH_2γ2$	1,13	24,68
**Thr3**					$CH_3γ$	0,92	15,32
	NH	8,21	-		$CH_3δ$	0,85	10,82
	CHα	4,32	59,18				

Table S5. Continued

CO	-	170,93	**Ile8**			
CH_2β	5,16	71,01		NH	7,25	-
CHγ	1,27	18,09		CHα	4,48	57,49
				CO	-	172,8
				CHβ	2,07	36,41
				CH_2γ1	1,61	25,17
				CH_2γ2	1,26	25,16
				CH_3γ	0,99	15,43
				CH_3δ	0,9	10,68

Table S6. ^{1}H and ^{13}C chemical shift values of xantholysin, extracted from the medium extract of *P. xantholysinigenes* RW9S1A^{T} with retention time 17.9 minutes (500 MHz, DMF-d7, 328 K).

		^{1}H δ [ppm]	^{13}C δ [ppm]
**HDA**			
	CO	-	n.d.
	CH$_2$α	2,47	53,61
	CHβ	4,03	68,31
	CH$_2$γ	1,51	37,36
	CH2 δ1	1,46	25,32
	CH2 δ2	1,35	25,32
	CH2 ε	1,31	29,43
	CH2 ζ	1,29	29,08
	CH$_2$ η	1,29	31,64
	CH2 θ	1,3	22,34
	CH$_3$	0,89	13,45
	OH	n.d.	-
**Leu1**			
	NH	8,46	-
	CHα	4,33	52,21
	CO	-	n.d.
	CH$_2$β	1,72	39,77
	CHγ	1,77	24,72
	CH$_3$δ1	0,95	22,73
	CH$_3$δ2	0,92	21,47
**Glu2**			
	NH	8,68	-
	CHα	4,26	55,08
	CO	-	n.d.
	CH$_2$β1	2,16	26,31
	CH$_2$β2	2,07	26,31
	CH$_2$γ	2,5	30,39
	COδ	-	n.d.
	OH	n.d.	-
**Gln3**			
	NH	8,43	-
	CHα	4,27	55,42
	CO	-	n.d.
	CH$_2$β	2,15	26,82
	CH$_2$γ	2,43	32,02
	COδ	-	n.d.
	NH$_2$1	n.d.	-
	NH$_2$2	n.d.	-
**Gln6**			
	NH	8,29	-
	CHα	4,09	53,43
	CO	-	n.d.
	CH$_2$β1	2,26	26,8
	CH$_2$β2	2,05	26,8
	CH$_2$γ	2,3	31,66
	COδ	-	n.d.
	NH$_2$1	n.d.	-
	NH$_2$2	n.d.	-
**Ser7**			
	NH	7,9	-
	CHα	4,58	54,17
	CO	-	n.d.
	CH$_2$β1	4,53	63,42
	CH$_2$β2	4,38	63,42
**Val8**			
	NH	8,22	-
	CHα	4,11	61,61
	CO	-	n.d.
	CHβ	2,43	29,74
	CH$_3$γ1	1,09	18,71
	CH$_3$γ2	0,99	18,99
**Leu9**			
	NH	8,08	-
	CHα	4,2	54,27
	CO	-	n.d.
	CH$_2$β1	1,71	39,75
	CH$_2$β2	1,6	39,75
	CHγ	1,87	24,43
	CH$_3$δ1	0,93	22,66
	CH$_3$δ2	0,87	20,7
**Gln10**			
	NH	7,74	-
	CHα	4,34	53,86
	CO	-	n.d.
	CH$_2$β1	2,17	26,87
	CH$_2$β2	2,01	26,87
	CH$_2$γ	2,4	61,96

Table S6. Continued.

**Val4**			
	NH	7,68	-
	CHα	4,01	61,43
	CO	-	n.d.
	CHβ	2,27	29,51
	$CH_3γ1$	1,03	19,11
	$CH_3γ2$	0,96	18,81
**Leu5**			
	NH	7,9	-
	CHα	4,18	54,15
	CO	-	n.d.
	$CH_2β$	1,75	39,95
	CHγ	1,74	24,43
	$CH_3δ1$	0,94	22,12
	$CH_3δ2$	0,91	21,03
**Leu12**			
	NH	7,78	-
	CHα	4,35	52,7
	CO	-	n.d.
	$CH_2β1$	1,75	40,53
	$CH_2β2$	1,67	40,53
	CHγ	1,78	24,61
	$CH_3δ1$	0,97	22,68
	$CH_3δ2$	0,9	20,92
**Gln13**			
	NH	7,26	-
	CHα	4,26	53,76
	CO		
	$CH_2β1$	2,34	26,81
	$CH_2β2$	1,95	26,81
	$CH_2γ$	2,23	31,75
	COδ	-	n.d.
	NH_21	n.d.	-
	NH_22	n.d.	-
	COδ	-	n.d.
	NH_21	n.d.	-
	NH_22	n.d.	-
**Leu11**			
	NH	7,92	-
	CHα	4,33	53,11
	CO	-	n.d.
	$CH_2β1$	1,82	40,75
	$CH_2β2$	1,6	40,75
	CHγ	1,84	26,63
	$CH_3δ1$	0,94	22,7
	$CH_3δ2$	0,88	20,82
**Ile14**			
	NH	7,49	-
	CHα	4,37	57,02
	CO	-	n.d.
	CHβ	1,9	36,53
	$CH_2γ1$	1,4	24,82
	$CH_2γ2$	1,18	24,82
	$CH_3γ$	0,88	15,32
	$CH_3δ$	0,85	10,69

Table S7. ^{1}H and ^{13}C chemical shift values of the main compound (putisolvin III) of the medium extract of *P. fulva* LMG 11722^{T} with retention time 11.2 minutes (700 MHz, DMF-d7, 298 K).

		^{1}H δ [ppm]	^{13}C δ [ppm]			^{1}H δ [ppm]	^{13}C δ [ppm]
**FA**				**Ser6**			
	CO	-	174,74		NH	8,27	-
	$CH_2\alpha1$	2,34	35,37		CHα	4,54	58,35
	$CH_2\alpha2$	2,27	35,37		CO	-	n.d.
	CHβ	1,62	25,35		$CH_2\beta$	4,01	60,65
	$CH_2\gamma$	1,31	31,31		OH	5,3	-
	$CH_2\delta$	1,31	22,28				
	CH_3	0,88	13,59	**Val7**			
	OH	n.d.	-		NH	8,01	-
					CHα	3,89	62,73
**Leu1**					CO	-	174,32
	NH	8,41	-		CHβ	2,35	29,13
	CHα	4,28	52,91		$CH_3\gamma1$	1,14	19,63
	CO	-	174,33		$CH_3\gamma2$	1,08	18,86
	$CH_2\beta$	1,65	40,09				
	CHγ	1,74	24,62	**Leu8**			
	$CH_3\delta1$	0,96	22,52		NH	8,01	-
	$CH_3\delta2$	0,91	21,47		CHα	4,12	54,49
					CO	-	171,23
**Glu2**					$CH_2\beta1$	1,99	38,93
	NH	8,75	-		$CH_2\beta2$	1,56	38,93
	CHα	4,36	53,91		CHγ	1,87	24,77
	CO	-	n.d.		$CH_3\delta1$	0,9	21,17
	$CH_2\beta1$	2,19	26,48		$CH_3\delta2$	0,99	22,82
	$CH_2\beta2$	1,97	26,5				
	$CH_2\gamma$	2,49	30,36	**Ser9**			
	COδ	-	174,37		NH	7,39	-
	OH	n.d.	-		CHα	5,03	51,38
					CO	-	169,33
**Leu3**					$CH_2\beta1$	4,9	66,7
	NH	8,21	-		$CH_2\beta2$	4,02	66,71
	CHα	4,2	53,85				
	CO	-	175,22	**Leu10**			
	$CH_2\beta1$	1,86	39,48		NH	7,68	-
	$CH_2\beta2$	1,63	39,49		CHα	4,26	54,04
	CHγ	1,75	24,62		CO	-	172,33
	$CH_3\delta2$	0,9	20,29		$CH_2\beta1$	2,02	40
	$CH_3\delta1$	0,96	22,51		$CH_2\beta2$	1,61	40,03
					CHγ	1,84	24,72
**Leu4**					$CH_3\delta1$	0,88	20,6

Table S7. Continued.

	NH	8,13	-		$CH_3δ2$	0,98	22,65
	CHα	4,09	52,64				
	CO	-	171,23	**Val11**			
	$CH_2β1$	1,81	39,02		NH	7,22	-
	$CH_2β2$	1,74	39,01		CHα	4,1	61,74
	CHγ	1,73	24,61		CO	-	171,23
	$CH_3δ1$	0,95	22,6		CHβ	1,85	29,43
	$CH_3δ2$	0,91	21,47		$CH_3γ1$	0,91	18,64
					$CH_3γ2$	0,87	19,03
**Gln5**							
	NH	8,65	-	**Ser12**			
	CHα	4,09	55,84		NH	8,32	-
	CO	-	171,23		CHα	4,77	54,71
	$CH_2β$	2,15	26,38		CO	-	170,55
	$CH_2γ$	2,42	31,46		$CH_2β1$	4,03	62,99
	COδ	-	174,49		$CH_2β2$	3,69	62,99
	NH_21	7,49	-		OH	5,48	-
	NH_22	6,93	-				

Table S8. ^{1}H and ^{13}C chemical shift values of the first minor compound (putisolvin IV) of the medium extract of *P. fulva* LMG 11722^{T} with retention time 12.6 minutes (700 MHz, DMF-d7, 298 K).

		^{1}H δ [ppm]	^{13}C δ [ppm]			^{1}H δ [ppm]	^{13}C δ [ppm]
**FA**				**Ser6**			
	CO	-	174,3		NH	8,32	-
	$CH_2\alpha 1$	2,33	35,4		CHα	4,55	58,25
	$CH_2\alpha 2$	2,27	35,4		CO	-	n.d.
	CHβ	1,61	25,32		$CH_2\beta$	4	60,5
	$CH_2\gamma$	1,3	31,37		OH	n.d.	-
	$CH_2\delta$	1,31	22,3				
	CH_3	0,88	13,62	**Val7**			
	OH	n.d.	-		NH	8,09	-
					CHα	3,86	62,82
**Leu1**					CO	-	n.d.
	NH	8,37	-		CHβ	2,35	29,24
	CHα	4,32	52,79		$CH_3\gamma 1$	1,16	20
	CO	-	n.d.		$CH_3\gamma 2$	1,08	18,82
	$CH_2\beta$	1,63	40,23				
	CHγ	1,73	24,64	**Leu8**			
	$CH_3\delta 1$	0,96	22,58		NH	8,01	-
	$CH_3\delta 2$	0,91	21,47		CHα	4,12	54,52
					CO	-	n.d.
**Glu2**					$CH_2\beta 1$	2	38,95
	NH	8,68	-		$CH_2\beta 2$	1,56	38,95
	CHα	4,4	53,72		CHγ	1,89	24,76
	CO	-	n.d.		$CH_3\delta 1$	0,9	21,31
	$CH_2\beta 1$	2,18	26,67		$CH_3\delta 2$	0,99	22,87
	$CH_2\beta 2$	1,97	26,67				
	$CH_2\gamma$	2,48	30,39	**Ser9**			
	COδ	-	174,49		NH	7,4	-
	OH	n.d.	-		CHα	5,03	51,38
					CO	-	169,18
**Leu3**					$CH_2\beta 1$	4,91	66,7
	NH	8,26	-		$CH_2\beta 2$	4,01	66,7
	CHα	4,22	53,91				
	CO	-	n.d.	**Leu10**			
	$CH_2\beta 1$	1,82	39,51		NH	7,69	-
	$CH_2\beta 2$	1,67	39,54		CHα	4,25	54,11
	CHγ	1,75	24,67		CO	-	n.d.
	$CH_3\delta 2$	0,9	20,3		$CH_2\beta 1$	2,01	40,04
	$CH_3\delta 1$	0,96	22,35		$CH_2\beta 2$	1,58	40,04
					CHγ	1,85	24,73
**Leu4**					$CH_3\delta 1$	0,88	20,61

Table S8. Continued.

	NH	8,3	-		$CH_3\delta2$	0,98	22,67
	CHα	4,13	52,3				
	CO	-	n.d.	**Ile11**			
	$CH_2\beta1$	1,83	39,08		NH	7,26	-
	$CH_2\beta2$	1,78	39,06		CHα	4,19	60,04
	CHγ	1,73	24,64		CO	-	171,2
	$CH_3\delta1$	0,93	22,75		CHβ	1,69	35,2
	$CH_3\delta2$	0,91	21,47		$CH_2\gamma1$	1,51	25,21
					$CH_2\gamma2$	1,03	25,22
**Gln5**					$CH_3\gamma$	0,87	14,79
	NH	8,58	-		$CH_3\delta$	0,85	10,2
	CHα	4,09	55,97				
	CO	-	n.d.	**Ser12**			
	$CH_2\beta$	2,15	26,35		NH	8,3	-
	$CH_2\gamma$	2,43	31,47		CHα	4,77	54,67
	COδ	-	174,49		CO	-	170,43
	NH_21	7,5	-		$CH_2\beta1$	4,02	63,03
	NH_22	6,95	-		$CH_2\beta2$	3,69	63,03
					OH	5,47	-

Table S9. ^{1}H and ^{13}C chemical shift values of the second minor compound (putisolvin V) of the medium extract of *P. fulva* LMG 11722^{T} with retention time 13.2 minutes (700 MHz, DMF-d7, 298 K).

		^{1}H δ [ppm]	^{13}C δ [ppm]
**HDA**			
	CO	-	n.d.
	CH_2α1	2,32	35,37
	CH_2α2	2,27	35,37
	CH_2β	1,62	25,32
	CH_2γ	1,3	31,37
	CH_2δ	1,31	22,32
	CH_2ε	0,88	13,6
**Leu1**			
	NH	8,37	-
	CHα	4,33	52,71
	CO	-	n.d.
	CH_2β1	1,63	40,27
	CH_2β2	1,63	40,27
	CHγ	1,72	24,59
	CH_3δ1	0,96	22,27
	CH_3δ2	0,91	21,43
**Glu2**			
	NH	8,66	-
	CHα	4,39	53,73
	CO	-	n.d.
	CH_2β1	2,17	26,63
	CH_2β2	1,97	26,63
	CH_2γ	2,47	30,35
	COδ	-	n.d.
	OH	n.d.	-
**Leu3**			
	NH	8,26	-
	CHα	4,21	53,95
	CO	-	n.d.
	CH_2β1	1,8	39,59
	CH_2β2	1,69	39,59
	CHγ	1,76	24,69
	CH_3δ1	0,95	22,6
	CH_3δ2	0,9	21,22
**Leu4**			
	NH	8,32	
	CHα	4,12	52,29
	CO	-	n.d.

		^{1}H δ [ppm]	^{13}C δ [ppm]
**Ser6**			
	NH	8,3	-
	CHα	4,59	57,88
	CO	-	n.d.
	CH_2β1	4	60,78
	CH_2β2	4	60,78
	OHγ	5,37	-
**Val7**			
	NH	8,27	-
	CHα	3,88	62,99
	CO	-	n.d.
	CHβ	2,36	29,22
	CH_3δ1	1,15	19,92
	CH_3δ2	1,07	18,8
**Leu8**			
	NH	8,03	-
	CHα	4,12	54,51
	CO	-	n.d.
	CH_2β1	2	38,98
	CH_2β2	1,55	38,98
	CHγ	1,89	24,75
	CH_3δ1	0,97	22,83
	CH_3δ2	0,9	20,35
**Ser9**			
	NH	7,46	-
	CHα	5,01	51,55
	CO	-	n.d.
	CH_2β1	4,95	66,59
	CH_2β2	4,01	66,59
	OHγ	n.d.	-
**Leu10**			
	NH	7,66	-
	CHα	4,25	54,25
	CO	-	n.d.
	CH_2β1	1,99	40,03
	CH_2β2	1,6	40,03
	CHγ	1,84	24,72
	CH_3δ1	0,98	22,57
	CH_3δ2	0,88	20,72

Table S9. Continued.

	$CH_2β1$	1,79	39	**Leu11**			
	$CH_2β2$	1,79	39		NH	7,33	-
	CHγ	1,73	24,74		CHα	4,63	53,17
	$CH_3δ1$	0,95	22,64		CO	-	n.d.
	$CH_3δ2$	0,9	21,17		$CH_2β1$	1,6	39,9
					$CH_2β2$	1,4	39,9
**Gln5**					CHγ	1,81	24,67
	NH	8,5	-		$CH_3δ1$	0,92	22,78
	CHα	4,09	55,9		$CH_3δ2$	0,89	20,89
	CO	-	n.d.				
	$CH_2β$	2,15	26,44	**Ser12**			
	$CH_2γ$	2,41	31,43		NH	8,21	-
	COδ	-	n.d.		CHα	4,67	55,2
	NH_2	7.47/6.93	-		CO	-	n.d.
					$CH_2β1$	4,01	62,92
					$CH_2β2$	3,71	62,91
					OHγ	5,56	-

Table S10. ^{1}H and ^{13}C chemical shift values of tensin of the medium extract of *P. zeae* OE 48.2^{T} (700 MHz, DMF-d7, 298 K).

		^{1}H δ [ppm]	^{13}C δ [ppm]			^{1}H δ [ppm]	^{13}C δ [ppm]
**HDA**				**Ser6**			
	CO	-	172,9		NH	7,71	-
	$CH_2\alpha$	2,46	43,54		CHα	4,51	55,99
	CHβ	4,04	68,56		CO	-	171,3
	$CH_2\gamma$	1,51	37,38		$CH_2\beta1$	4,01	63,11
	$CH_2\delta1$	1,49	25,29		$CH_2\beta2$	3,76	63,11
	$CH_2\delta2$	1,37	25,29		OH	7,72	-
	$CH_2\,\varepsilon$	1,31	29,31				
	$CH_2\,\zeta$	1,31	29,31	**Leu7**			
	$CH_2\,\eta$	1,29	31,65		NH	7,77	-
	$CH_2\,\theta$	1,3	22,31		CHα	4,34	52,34
	$CH_3\,\iota$	0,89	13,4		CO	-	172,5
	OH	n.d.	-		$CH_2\beta$	1,76	29,68
					CHγ	1,79	24,61
**Leu1**					$CH_3\delta1$	0,94	22,69
	NH	8,29	-		$CH_3\delta2$	0,88	21,28
	CHα	4,32	53,14				
	CO	-	173,7	**Gln8**			
	$CH_2\beta$	1,63	40,35		NH	8,05	-
	CHγ	1,8	24,65		CHα	4,38	53,82
	$CH_3\delta1$	0,96	22,49		CO	-	n.d.
	$CH_3\delta2$	0,91	21,27		$CH_2\beta1$	2,07	27,85
					$CH_2\beta2$	1,93	27,82
**Asp2**					$CH_2\gamma$	2,31	31,8
	NH	8,33	-		COδ	-	174,3
	CHα	4,63	51,4		NH_2	7.27/6.64	-
	CO	-	171,9				
	$CH_2\beta1$	2,86	35,33	**Leu9**			
	$CH_2\beta2$	2,76	35,23		NH	8,18	-
	COδ	-	171,3		CHα	4,42	52,55
	OH	n.d.	-		CO	-	172,4
					$CH_2\beta$	1,69	40,78
**Thr3**					CHγ	1,75	24,5
	NH	7,74	-		$CH_3\delta1$	0,94	22,53
	CHα	4,73	57,65		$CH_3\delta2$	0,89	21,16
	CO	-	170,1				
	$CH_2\beta$	5,25	70,53	**Ile10**			
	CHγ	1,33	16,87		NH	7,87	-
					CHα	4,26	58,62
**Leu4**					CO	-	n.d.
	NH	8,03	-		CHβ	2,03	35,88
	CHα	4,4	52,93		$CH_2\gamma1$	1,61	25,33
	CO	-	n.d.		$CH_2\gamma2$	1,24	25,33
	$CH_2\beta1$	1,74	41,16		$CH_3\gamma$	0,98	15,26

Table S10. Continued.

	$CH_2\beta 2$	1,65	41,16		$CH_3\delta$	0,89	10,37
	CHγ	1,79	2461				
	$CH_3\delta 1$	0,91	22,69	**Glu11**			
	$CH_3\delta 2$	0,89	21,29		NH	7,32	-
					CHα	4,72	51,71
**Leu5**					CO	-	171,4
	NH	8,12	-		$CH_2\beta 1$	2,04	27,87
	CHα	4,4	52,93		$CH_2\beta 2$	1,83	27,87
	CO	-	n.d.		$CH_2\gamma$	2,31	29,93
	$CH_2\beta$	1,66	40,39		COδ	-	n.d.
	CHγ	1,77	25,54		OH	n.d.	-
	$CH_3\delta 1$	0,94	22,96				
	$CH_3\delta 2$	0,88	21,04				

Table S11. ^{1}H and ^{13}C chemical shift values of the main compound (tensin) of the medium extract of *P. zeae* OE 48.2^{T} with retention time 8.7 minutes for comparison with published tensin and milkisin chemical shifts (500 MHz, acetone-d6, 298 K).

		^{1}H δ [ppm]	^{13}C δ [ppm]			^{1}H δ [ppm]	^{13}C δ [ppm]
				**Ser6**			
**HDA**	CO	-	n.d.		NH	7,34	-
	$CH_2\alpha 1$	2,6	44,03		CHα	4,49	57,23
	$CH_2\alpha 2$	2,54	44,03		CO	-	n.d.
	CHβ	4,2	70,12		$CH_2\beta 1$	4,05	64,63
	$CH_2\gamma$	1,59	38,47		$CH_2\beta 2$	3,81	64,58
	$CH_2\delta 1$	1,51	26,21		OH	6,13	-
	$CH_2\delta 2$	1,38	26,21				
	$CH_2\,\varepsilon$	1,31	29,95	**Leu7**			
	$CH_2\,\zeta$	1,31	29,95		NH	7,65	-
	$CH_2\,\eta$	1,28	32,46		CHα	4,34	53,35
	$CH_2\,\theta$	1,29	23,16		CO	-	n.d.
	$CH_3\,\iota$	0,88	14,17		$CH_2\beta 1$	1,93	39,69
	OH	n.d.	-		$CH_2\beta 2$	1,67	39,69
					CHγ	1,86	25,19
**Leu1**					$CH_3\delta 1$	0,96	23,94
	NH	8,44	-		$CH_3\delta 2$	0,86	21,34
	CHα	4,19	55,71				
	CO	-	n.d.	**Gln8**			
	$CH_2\beta 1$	1,75	40,45		NH	8,03	-
	$CH_2\beta 2$	1,66	40,45		CHα	4,32	54,23
	CHγ	1,84	25,25		CO		
	$CH_3\delta 1$	0,99	22,73		$CH_2\beta 1$	2,03	28,81
	$CH_3\delta 2$	0,94	22,02		$CH_2\beta 2$	1,97	28,81
					$CH_2\gamma$	2,29	32,35
					COδ	-	n.d.
	NH	8,33	-		NH_2	7.07/6.35	-
	CHα	4,48	53,98				
	CO	-	n.d.	**Leu9**			
	$CH_2\beta 1$	2,86	35,3		NH	7,94	-
	$CH_2\beta 2$	2,8	35,3		CHα	4,22	53,81
	COδ	-	n.d.		CO	-	n.d.
	OH	n.d.	-		$CH_2\beta$	1,68	40,91
					CHγ	1,8	25,34
**Thr3**					$CH_3\delta 1$	0,93	23,43
	NH	7,79	-		$CH_3\delta 2$	0,88	21,29
	CHα	4,61	60,3				
	CO	-	n.d.	**Ile10**			
	$CH_2\beta$	5,53	70,12		NH	7,38	-
	CHγ	1,46	17,77		CHα	4,05	59,71
					CO	-	n.d.
					CHβ	2,04	36,5

Table S11. Continued.

	NH	7,87	-		$CH_2\gamma1$	1,57	26,06
	CHα	4,04	55,94		$CH_2\gamma2$	1,23	26,06
	CO	-	n.d.		$CH_3\gamma$	0,95	15,93
	$CH_2\beta1$	1,76	41,21		$CH_3\delta$	0,89	11,23
	$CH_2\beta2$	1,69	41,21				
	CHγ	1,77	25,19	**Glu11**			
	$CH_3\delta1$	0,93	22,94		NH	6,87	-
	$CH_3\delta2$	0,9	22,48		CHα	4,61	52,98
					CO	-	n.d.
**Leu5**					$CH_2\beta1$	1,98	29,03
	NH	7,79	-		$CH_2\beta2$	1,83	29,03
	CHα	4,26	53,87		$CH_2\gamma1$	2,36	30,34
	CO	-	n.d.		$CH_2\gamma2$	2,3	30,34
	$CH_2\beta1$	1,79	40,65		COδ	-	n.d.
	$CH_2\beta2$	1,55	40,65		OH	n.d.	-
	CHγ	1,92	25,27				
	$CH_3\delta1$	0,91	23,65				
	$CH_3\delta2$	0,87	20,87				

Table S12. Amino acid and nucleotide identity matrices based on, respectively, the concatenated NRPS enzymes (aa) and partial *pleB* gene (nt), including chemically characterized LP producers with complete NRPS clusters from the Bananamide family.

**Concatenated NRPS enzymes (aa)**		1	2	3	4
1	*P. prosekii* LMG 26867^T	100			
2	*P. granadensis* LMG 27940^T	65.85	100		
3	*P. botevensis* COW3^T	67.09	77.27	100	
4	*P. bananamidigenes* BW11P2^T	66.31	74.59	81.89	100
**Partial *pleB* gene (nt)**		1	2	3	4
1	*P. prosekii* LMG 26867^T	100			
2	*P. granadensis* LMG 27940^T	81.64	100		
3	*P. botevensis* COW3^T	83.18	85.86	100	
4	*P. bananamidigenes* BW11P2^T	81.38	85.28	85.94	100

Table S13. Amino acid and nucleotide identity matrices based on, respectively, the concatenated NRPS enzymes (aa) and partial *pleB* gene (nt), including chemically characterized LP producers with complete NRPS clusters from the Xantholysin and Entolysin families.

**Concatenated NRPS enzymes (aa)**		1	2	3
1	*P. entomophila* L48^T	100		
2	*P. xantholysinigenes* RW9S1A^T	70.19	100	
3	*P. mosselii* BW11M1	70.52	81.86	100
**Partial *pleB* gene (nt)**		1	2	3
1	*P. entomophila* L48^T	100		
2	*P. xantholysinigenes* RW9S1A^T	89.65	100	
3	*P. mosselii* BW11M1	89.07	89.65	100

Table S14. Amino acid and nucleotide identity matrices based on, respectively, the concatenated NRPS enzymes (aa) and partial *pleB* gene (nt), including chemically characterized LP producers with complete NRPS clusters from the Putisolvin family.

Concatenated NRPS enzymes (aa)		1	2	3	4	5	6
1	*P. fulva* LMG 11722^T	100					
2	*P. capeferrum* WCS358	73.72	100				
3	*P. putida* PCL1445	73.57	99.27	100			
4	*P. putida* COR19	72.43	81.84	81.64	100		
5	*P. vlassakiae* WCU_64	72.12	80.79	80.59	88.89	100	
6	*P. vlassakiae* WCU_60	72.11	80.77	80.57	88.88	99.98	100
**Partial *pleB* gene (nt)**		1	2	3	4	5	6
1	*P. fulva* LMG 11722^T	100					
2	*P. capeferrum* WCS358	81.72	100				
3	*P. putida* PCL1445	81.49	99.54	100			
4	*P. putida* COR19	82.51	90.21	90.21	100		
5	*P. vlassakiae* WCU_64	81.38	89.18	89.18	92.94	100	
6	*P. vlassakiae* WCU_60	81.38	89.18	89.18	92.94	100	100

Table S15. Amino acid and nucleotide identity matrices based on, respectively, the concatenated NRPS enzymes (aa) and partial *pleB* gene (nt) including, chemically characterized LP producers with complete NRPS clusters from the Amphisin family.

Concatenated NRPS enzymes (aa)		1	2	3	4	5	6	7
1	*P. zeae* OE 48.2^T	100						
2	*Pseudomonas* sp. FhG100052	92.6	100					
3	*P. crudilactis* UCMA 17988^T	92.5	90.9	100				
4	*P. fluorescens* DSS73	90.5	89.5	91.2	100			
5	*P. fluorescens* HKI0770	85.8	87	86.2	89.3	100		
6	*Pseudomonas* sp. MIS38	77.8	78.4	77.8	79.6	81.5	100	
7	*P. koreensis* COR10	79.7	80.3	79.8	81.4	84	93.3	100
**Partial *pleB* gene (nt)**		1	2	3	4	5	6	7
1	*P. zeae* OE 48.2^T	100						
2	*Pseudomonas* sp. FhG100052	92	100					
3	*P. crudilactis* UCMA 17988^T	91	91.3	100				
4	*P. fluorescens* DSS73	90.9	90.9	92	100			
5	*P. fluorescens* HKI0770	89.9	90.4	90.5	90.2	100		
6	*Pseudomonas* sp. MIS38	83.1	83	83.4	82.3	84.4	100	
7	*P. koreensis* COR10	83.3	83.4	83.6	82.7	84.4	96.9	100

Table S16. Optimized culture conditions for chemical characterization.

Strain	Medium	Temperature	Shaking (rpm)	Time
P. prosekii LMG 26867^T	M9	23°C	180	48h
Pseudomonas sp. OE 48.2	TSB	28°C	180	24h
P. fulva LMG 11722^T	TSB	28°C	180	18h
Pseudomonas sp. RW9S1A	M9	23°C	180	48h

Figure S1. Expanded phylogenetic tree based on full-length MacB proteins of known LP producers (PvdT and PleB; ~ 660 aa). Maximum likelihood tree constructed using the JTT+F+I+G model (MEGA-X). Bootstrap values were calculated based on 1000 replications and only bootstrap values higher than 50% are indicated.

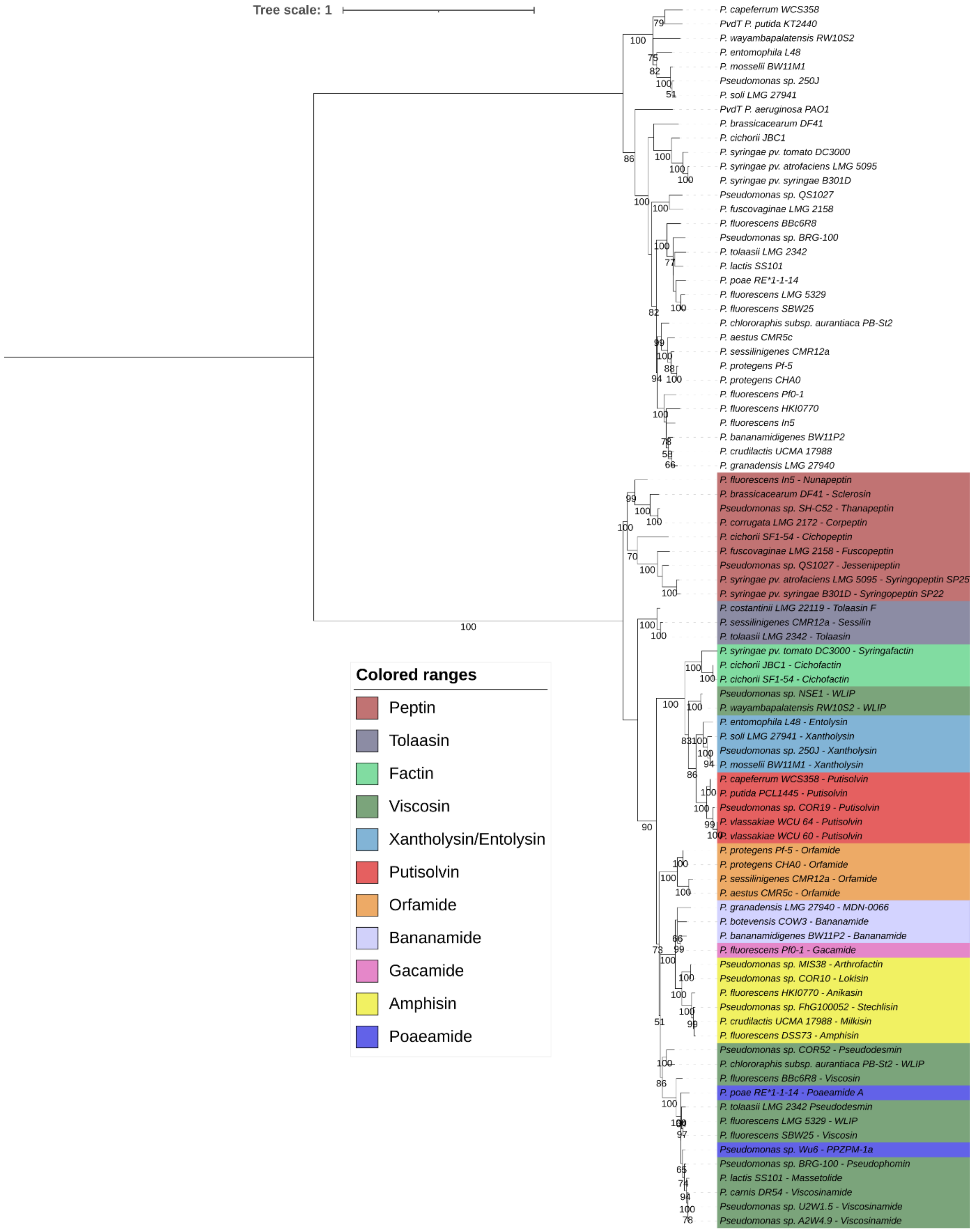

Figure S2. Phylogenetic tree based on concatenated NRPS proteins from the Amphisin family. Maximum likelihood tree constructed using the JTT+F+I+G model (MEGA-X). Genetically and/or chemically characterized producers are highlighted in bold. Pearson correlation between the *pleB* nucleotide identity and similarities between concatenated NRPSs of 0.8031 (p-value = 3.409e-106).

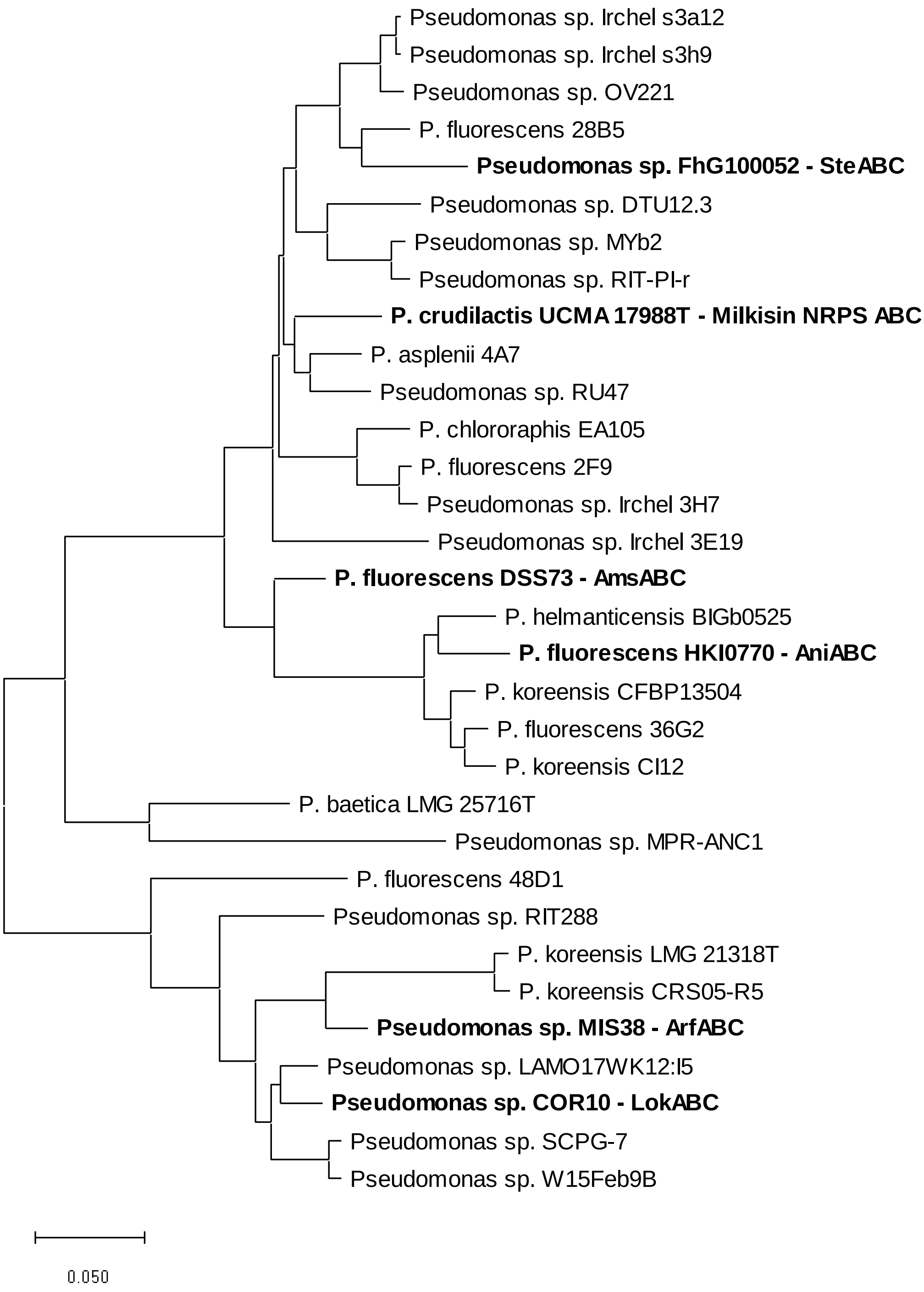

Figure S3. Phylogenetic tree based on concatenated NRPS proteins from the Gacamide family. Maximum likelihood tree constructed using the JTT+F+I+G model (MEGA-X). Genetically and/or chemically characterized producers are highlighted in bold. Pearson correlation between the *pleB* nucleotide identity and similarities between concatenated NRPSs of 0.7910 (p-value = 5.443e-7).

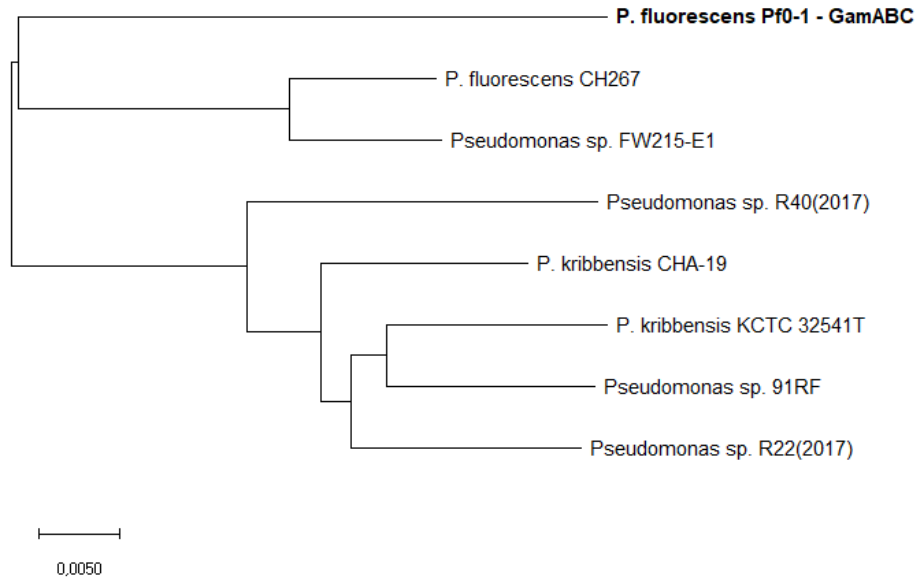

Figure S4. Phylogenetic tree based on concatenated NRPS proteins from the Bananamide family. Maximum likelihood tree constructed using the JTT+F+I+G model (MEGA-X). Genetically and/or chemically characterized producers are highlighted in bold. Pearson correlation between the *pleB* nucleotide identity and similarities between concatenated NRPSs of 0.9299 (p-value = 2.478e-92).

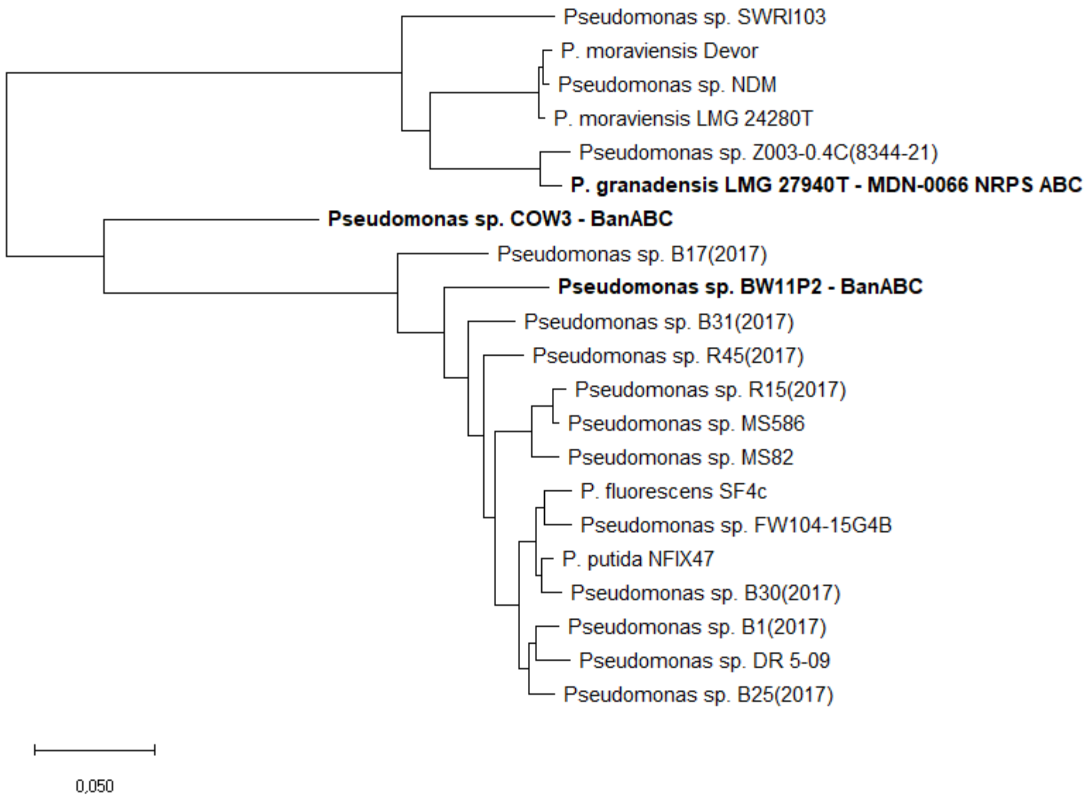

Figure S5. Phylogenetic tree based on concatenated NRPS proteins from the Putisolvin family. Maximum likelihood tree constructed using the JTT+F+I+G model (MEGA-X). Genetically and/or chemically characterized producers are highlighted in bold. Pearson correlation between the *pleB* nucleotide identity and similarities between concatenated NRPSs of 0.9607 (p-value = 1.51e-67).

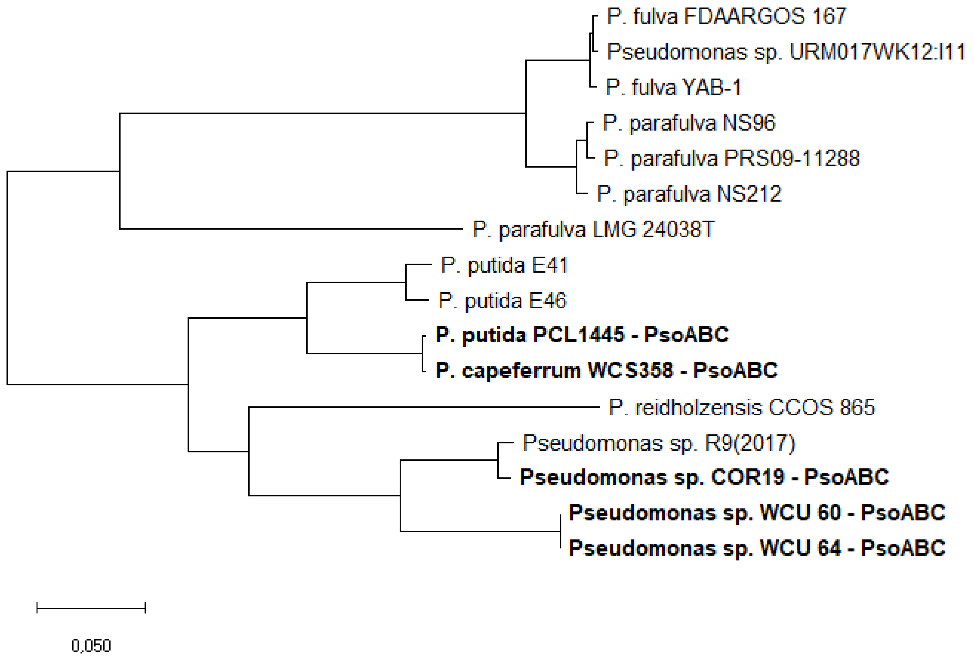

Figure S6. Phylogenetic tree based on concatenated NRPS proteins from the Xantholysin and Entolysin families. Maximum likelihood tree constructed using the JTT+F+I+G model (MEGA-X). Genetically and/or chemically characterized producers are highlighted in bold. Pearson correlation between the *pleB* nucleotide identity and similarities between concatenated NRPSs of 0.9171 (p-value = 6.882e-12). *P. soli* LMG27941^{T} and *Pseudomonas* sp. 250J are not included because their NRPS clusters are fragmented.

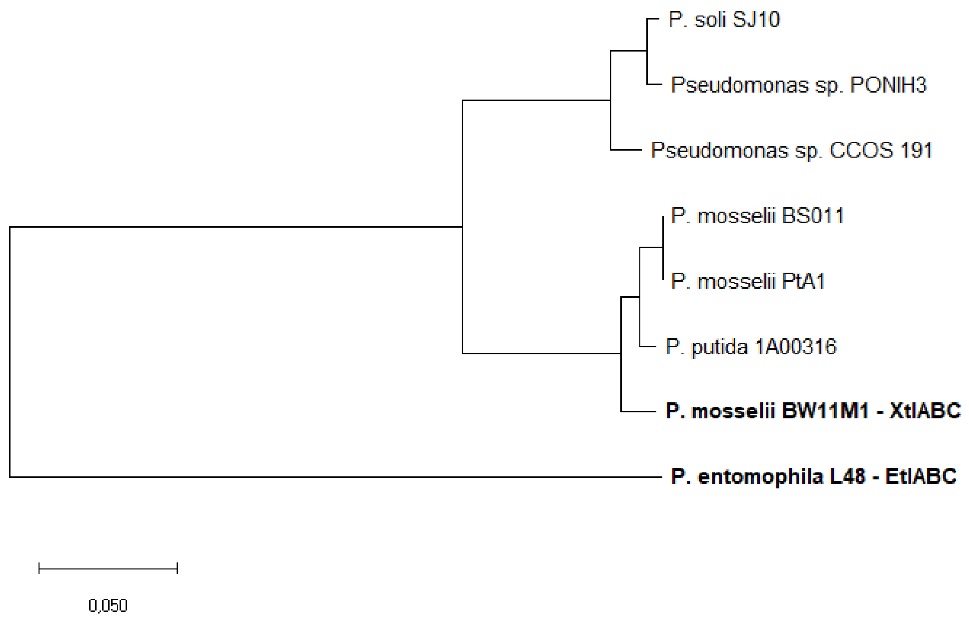

Figure S7. Phylogenetic tree based on concatenated NRPS proteins from the Tolaasin family. Maximum likelihood tree constructed using the JTT+F+I+G model (MEGA-X). Genetically and/or chemically characterized producers are highlighted in bold. Pearson correlation between the *pleB* nucleotide identity and similarities between concatenated NRPSs of 0.9445 (*p*-value = 5.227e-18).

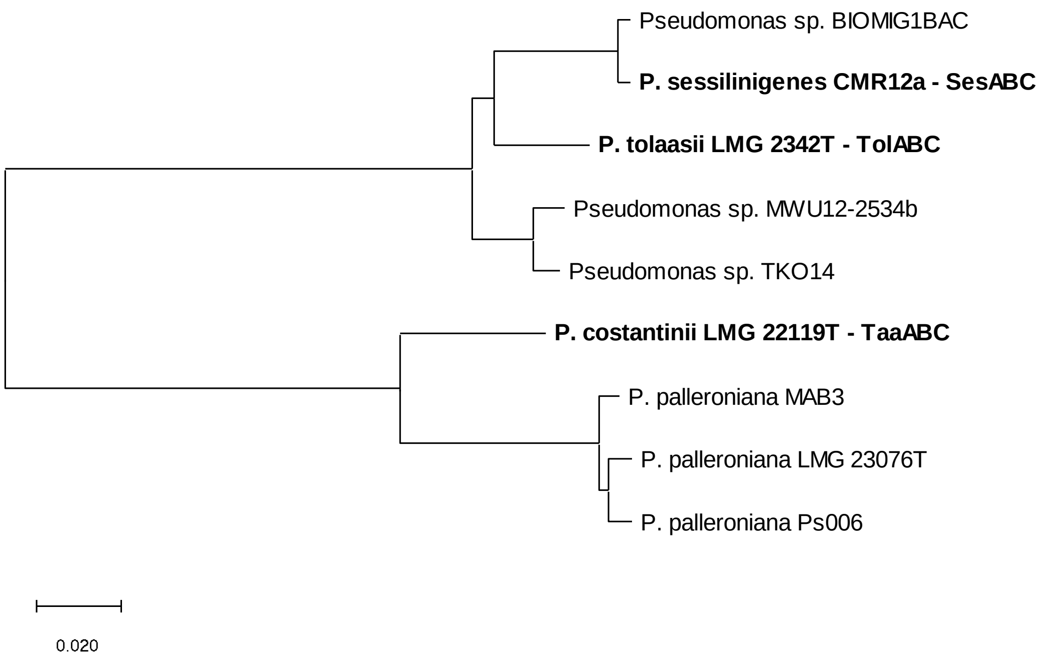

Figure S8. Gel electrophoresis of *pleB* amplicons obtained with the PutXanTol (A) and the BanAmpGac (B) primer sets. NC: Negative control. ML: Marker lane, SmartLadder 200 to 10,000 bp.

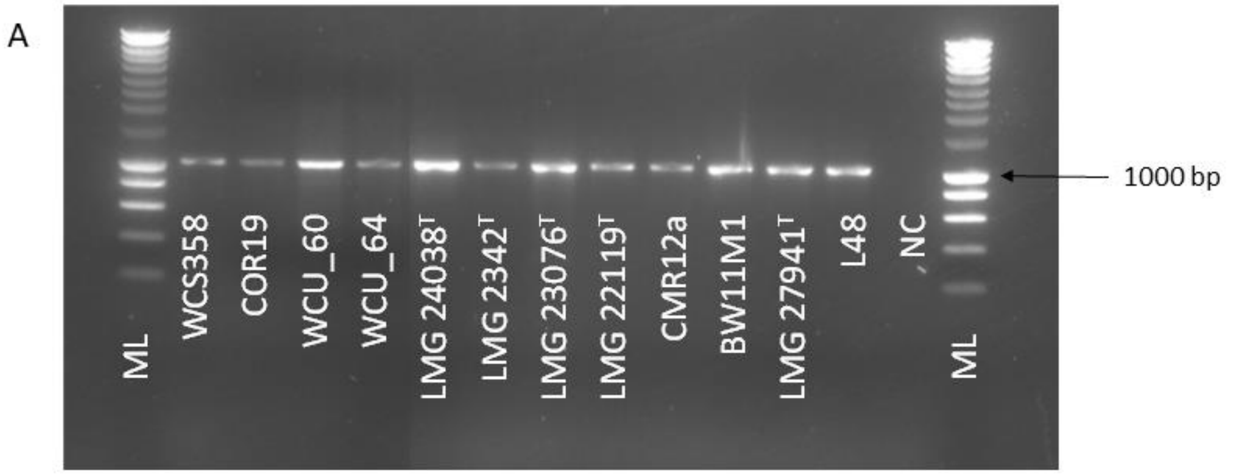

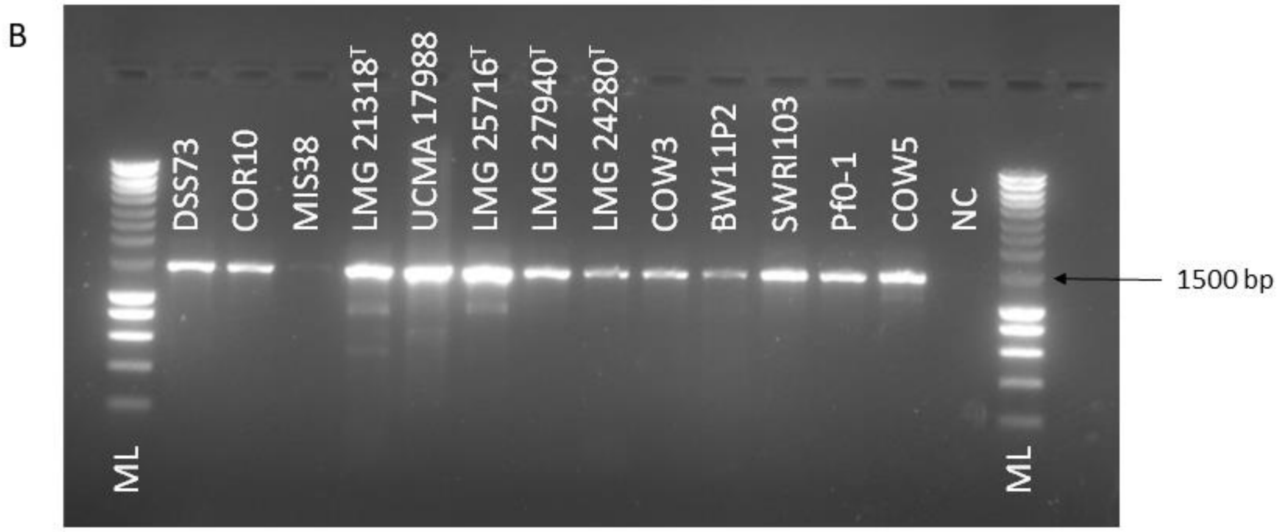

Figure S9. Position of primers PutXanTol F and R on *pleB* nucleotide sequence alignment of known LP producers from the Putisolvin, Xantholysin, Entolysin and Tolaasin families (Table 1).

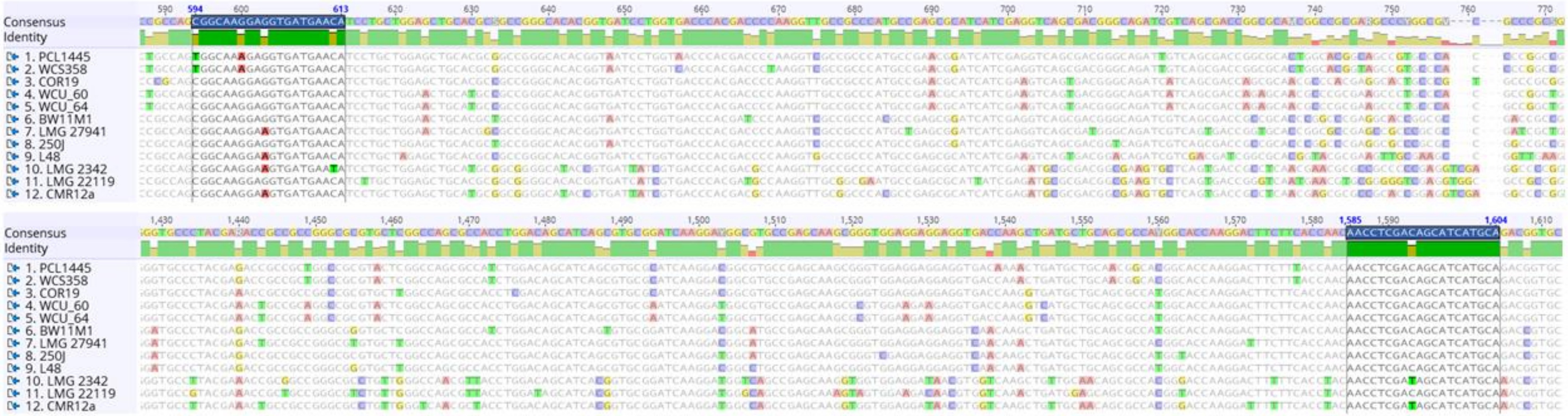

Figure S10. Position of primers BanAmpGac F and R on *pleB* amino nucleotide sequence alignment of known LP producers from the Bananamide, Amphisin and Gacamide families (Table 1).

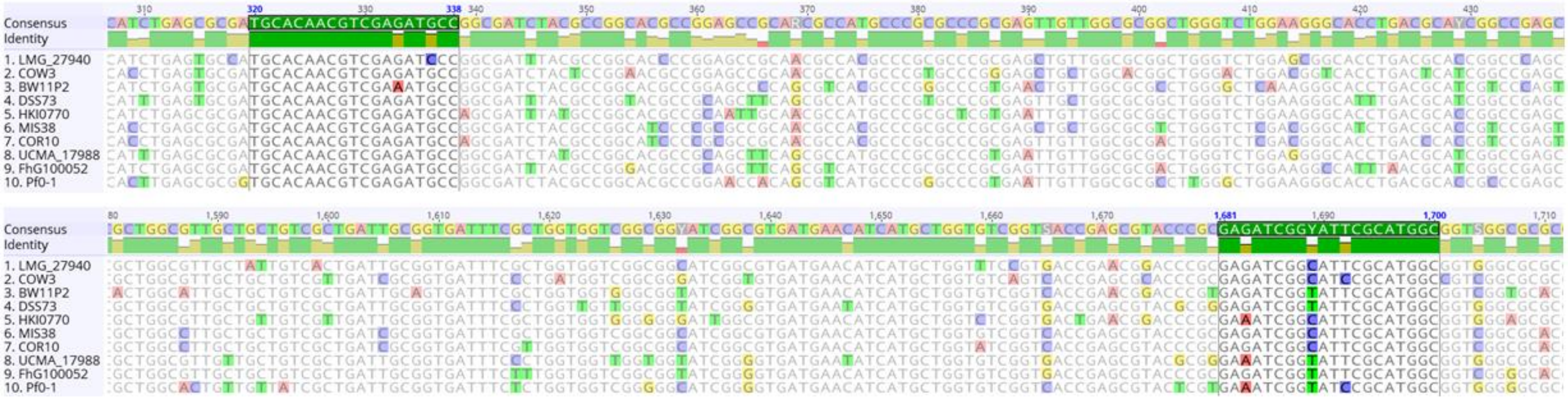

Figure S11. Sequence alignment of MacB proteins of *Escherichia coli* (Ec) and *Aggregatibacter actinomycetemcomitans* (Aa), PvdT of *Pseudomonas aeruginosa* (Pa) and PleB homologue ArfE of *Pseudomonas* sp. MIS38 (Psp.). The intracellular regions, four transmembrane segments, and periplasmic regions are indicated by, respectively, orange, yellow, and blue shading. The nucleotide binding domain is located in the N-terminal intracellular region. The Periplasmic Core Domain (MacB_PCD; Pfam PF12704) in PleB spans from position 291 to 512 of the alignment. The red box corresponds to the portion of the PleB protein corresponding to the final 880 bp fragment of the *pleB* gene used for LP BGC prospection.

```
             5          15         25         35         45         55         65         75         85         95         105        115        125        135        145
MacB_Ec    ------MTPL LELKDIRRSY -PAGDEQVEV LKGISLDIYA GEMVAIVGAS GSGKSTLMNI LGCLDKATSG TYRVAGQDVA TLDADALAQL RREHFGFIFQ RYHLLSHLTA EQNVEVPAVY AGLERKQRLL RAQELLQRLG LEDRTEYYPA
MacB_Aa    -------MNI IEIKQLNRYF -GEGENRVHV LKDISLSIER GDFVAIMGQS GSGKSTLMNI IGCLDTATGG SSKIDGKETI ELTNDQLSDL RSQKFGFIFQ RYNLLSSLTA AENVALPAIY AGMPQSQRLE RAKQLLEKLG LGDKWQNKPN
PvdT_Pa    MENATQPVPL IELRDIRKRY GGNGTPEVEV LKGVSLSIHA GEFVAIVGAS GSGKSTLMNI LGCLDRPSSG SYHFAGHDVA ELDSDEQAWL RREAFGFVFQ GYHLIPSASA QENVEMPAIY AGIPASERHT RARALLERLG LAERTANRPH
ArfE_Psp.  -----MTQAL LQLTGITRSF -TAGDREFLA LKDINLTINA GEMVAIIGAS GSGKSTLMNI LGCLDYATAG SYRINGQETR DLDDQALAEL RRDYFGFIFQ RYHLLPHLSA MHNVEMPAIY AGIPAPQRHA RARELLARLG LDGHLTHRPS

             155        165        175        185        195        205        215        225        235        245        255        265        275        285        295
MacB_Ec    QLSGGQQQRV SIARALMNGG QVILADEPTG ALDSHSGEEV MAILHQLRDR GHTVIIVTHD PQVAAQAERV IEIRDGEIV- --RN-----P PAIEKVNVTG GTEPVVNTVS GWR-QFVSG- --FNEALTMA WRALAANKMR TLLTMLGIII
MacB_Aa    QLSGGQQQRV SIARALMNGG EIILADEPTG ALDSHSGENV MEILRQLHEE GHTIIMVTHD KHIAASANRI IEIKDGEIIS DTQK-----R Q--------- -VKSAVKNPS VFKGRFGFSK DQLMEAFRMS VSAIVAHKMR SLLTMLGIII
PvdT_Pa    QLSGGQQQRV SIARALMNGG HIILADEPTG ALDSHSGAEV MALLDELASQ GHVVILITHD RDVAARAKRI IEVRDGEIVS DSANDERPAH PSAGVERHLQ ADDLSQRLAE GSSEPSGAWR AELLEAVRAA WRVMWINRFR TALTLLGIII
ArfE_Psp.  QLSGGQQQRV SIARALMNGG EVILADEPTG ALDTASGKEV MRILLELHAA GHTVILVTHD PKVAANAERI IEVSDGEILS DRRN-----Q RDTGQLS--- -DEDVPPKPP ATR-RLVASL GLFKEAFNMA WVALISHRMR TLLTMLGIVI

             305        315        325        335        345        355        365        375        385        395        405        415        425        435        445
MacB_Ec    GIASVVSIVV VGDAAKQMVL ADIRSIGTNT IDVYPGKDFG DDDPQYQQAL KYDDLIAIQK QPWVASATPA VSQNLRLRYN NVDV-AASAN GVSGDYFNVY GMTFSEGNTF NQEQLNGRAQ VVVLDSNTRR QLFPHKADVV GEVILVGNMP
MacB_Aa    GITSVVSVVA LGNGSQQKIL ENIRGIGTNT MTIFNGNGFG DRRSRHIQNL KISDANTLSK QSYIQSVTPN TSSSGILVVG NKSFTSANLY GIGEQYFDVE GLKLKQGRLL TEDDVDQSNQ VVVLDESAKK AIFAN-ENPL GKTVIFNKRP
PvdT_Pa    GVASVVVMLA VGEGSKRQVM AQMGAFGSNI IYL---SGYS PNPRAPMGIV SSDDVAAIAT LPQVKKVMPV NGGELVVRYG NIDY-HAYVG GNNTDFPEIL NWPVAEGSYF TERDEDAATT VAVIGYKVRK KLFGS-ANPI GRYILIENVP
ArfE_Psp.  GITSVVSISA IGEGAKNYVL KDISAIGSNT IDIYSGSSFG DKRAASIETL VPADVTALNQ LYYVDSATPV VGRSMLLRYR NIDL-DAQIN GVSDQYFQVR GIKLEAGITF SESDARRQAQ VVVIDYNTRH RLFGEGVDPL GQVILIGNLP

             455        465        475        485        495        505        515        525        535        545        555        565        575        585        595
MacB_Ec    ARVIGVAEEK QSMFGSSKV- LRVWLPYSTM SGRVMGQSWL NSITVRVKEG FDSAEAEQQL TRLLSLRH-G KKDFFTWNMD GVLKTVEKTT RTLQLFLTLV AVISLVVGGI GVMNIMLVSV TERTREIGIR MAVGARASDV LQQFLIEAVL
MacB_Aa    FRVIGVVSDQ QLGGFPGNS- LNLYSPYSTV LNKITGGSRI GSITVKISDD VNSTVAEKSL TELLKSLH-G KKDFFIMNSD TIKQTIENTT GTMKLLISSI AFISLIVGGI GVMNIMLVSV TERTKEIGVR MAIGARQINI LQQFLIEAVL
PvdT_Pa    FQVIGVLAEK GSSSGDKDAD NRIAIPYSAA SIRLFGTRNP EYVIIAAADA QRVHQAERAI DQLMLRLHRG QRDYELTNNA AMIQAEAKTQ NTLSLMLGSI AAISLLVGGI GVMNIMLMTV RERTREIGIR MATGARQGDI LRQFLTEAAM
ArfE_Psp.  CTVIGVAAEN KNLFASGKT- LNVWVPYETA AGRILGQRFL DSISVRIKDG QSSKVVENHV TQLMLQRH-G IKDFYTNNLD SVMQTVQKTS RSLALLLSLI AVISLVVGGI GVMNIMLVSV TERTREIGIR MAVGARQSDI RQQFLVEAVM

             605        615        625        635        645        655        665
MacB_Ec    VCLVGGALGI TLSLLIAFTL QLFLPGWEIG FSPLALLLAF LCSTVTGILF GWLPARNAAR LDPVDALARE
MacB_Aa    ICLIGGVAGI LLSVLIGVLF NSFITDFSMD FSTASIVTAV LFSTLIGVLF GYMPAKKAAE LNPITALAQE
PvdT_Pa    LSVVGGLAGI ALALCIGGVL --LLGQVAVA FSLSAIVGAF SCALVTGLVF GFMPARKAAQ LDPVAALASQ
ArfE_Psp.  VCLLGGAIGI SLSYAIGHLF SLFIKEWEMV FSMASVLTAV ICSTLIGIVF GFVPARNASR LDPIEALARD
```

Figure S12. Characterization of prosekin produced by *P. prosekii* LMG 26867^{T}. **A:** Representative preparative HPLC chromatogram of the medium extract of LMG 26867^{T}. The LP that was analysed is indicated by an arrow. Gradient: 25:75 to 0:100 H_2O:ACN in 20 minutes. **B:** 1D ^{1}H NMR spectrum of prosekin, eluting at 11.4 minutes. **C:** The alpha region of a ^{1}H-{^{13}C} gHSQC spectrum of the isolated LP shows the presence of 8 amino acids. The high chemical shift of the Thr3 CH^{β} indicates that the C-terminal ester bond is formed with this residue. **D:** Amide region of a ^{1}H-^{1}H TOCSY spectrum, showing all amino acids in the isolated LP.

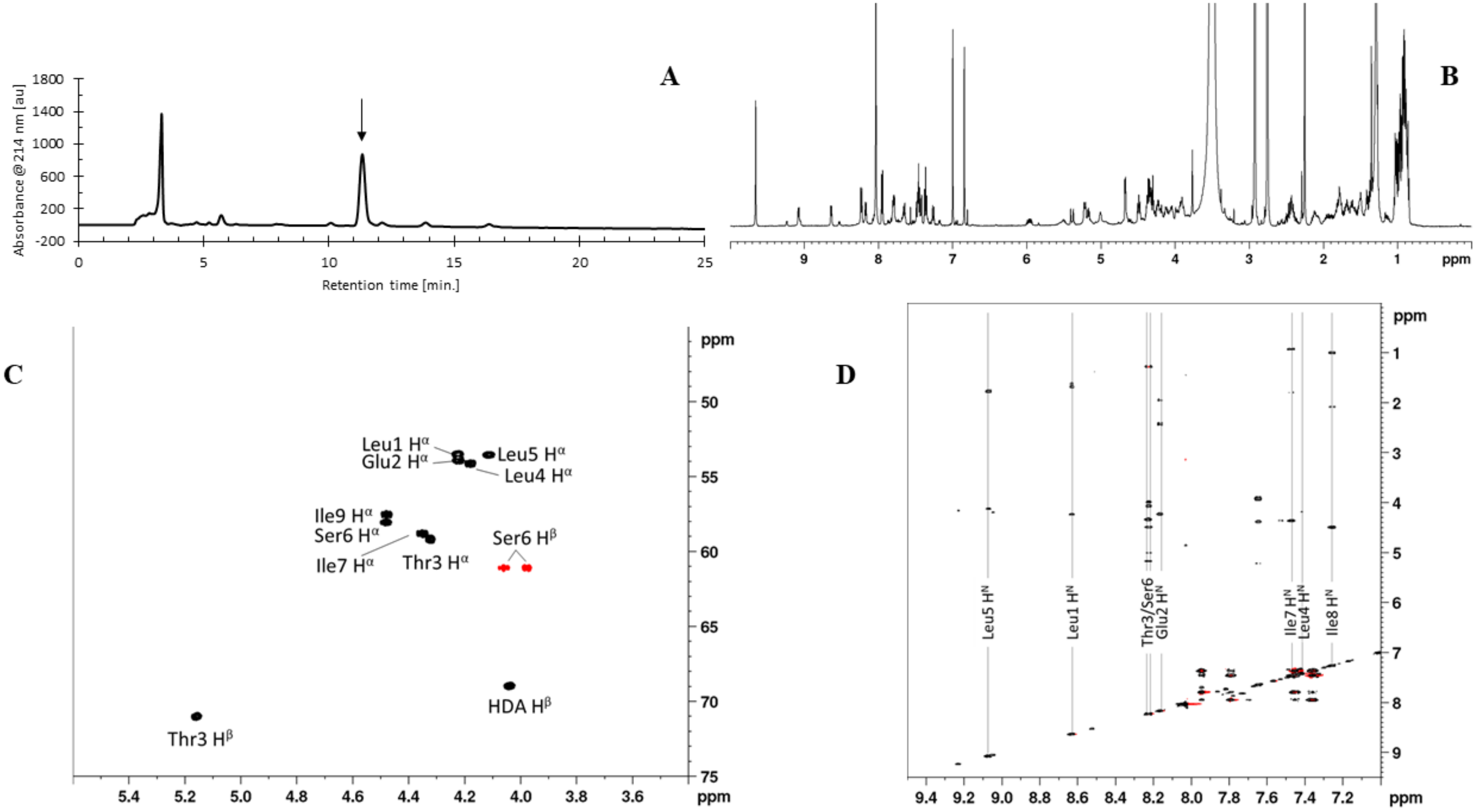

Figure S13. Characterization of xantholysin A produced by *P. xantholysinigenes* RW9S1A^T. **A:** Representative preparative HPLC chromatogram of the medium extract of RW9S1A^T. The LP that was analyzed is indicated by an arrow. Gradient: 25:75 to 0:100 H_2O:ACN in 20 minutes. **B:** 1D ^{1}H NMR spectrum of the isolated LP, xantholysin A, eluting at 17.9 minutes. **C:** The alpha region of a ^{1}H-{^{13}C} gHSQC spectrum of the isolated LP shows the presence of 14 amino acids. The high chemical shift of the Ser7 CH$^\beta$ indicates that the C-terminal ester bond is formed with this residue. **D:** Amide region of a ^{1}H-^{1}H TOCSY spectrum, showing all amino acids in the isolated LP.

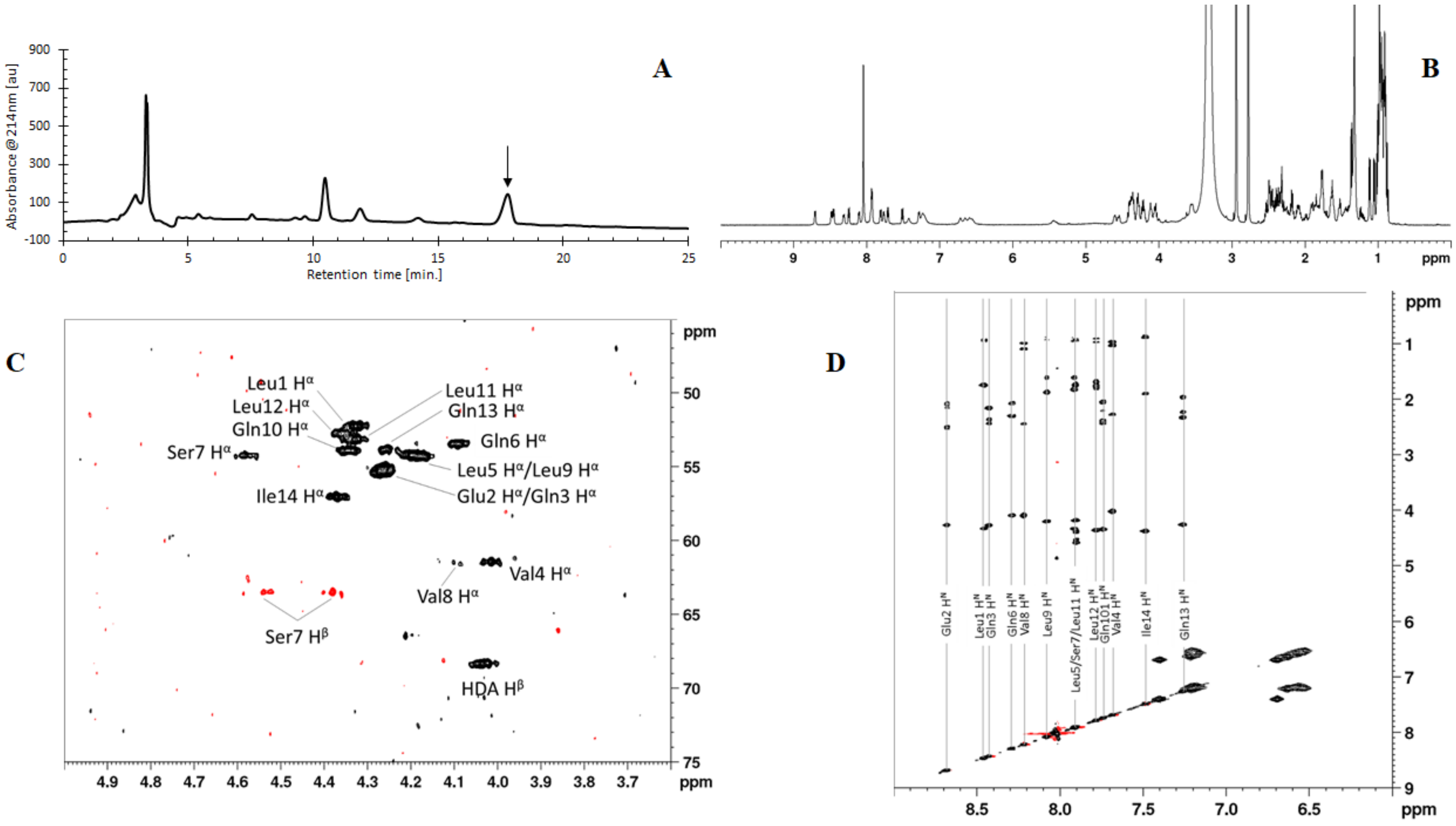

Figure S14. Characterization of putisolvins produced by *P. fulva* LMG 11722^T. **A:** Representative preparative HPLC chromatogram of the medium extract of LMG 11722^T. The LPs that were analyzed are indicated by arrows, respectively, putisolvin III, IV and V. Gradient: 25:75 to 0:100 H_2O:ACN in 20 minutes. **B:** 1D ^{1}H NMR spectrum of putisolvin III, eluting at 12.2 minutes. **C:** The alpha region of a ^{1}H-{^{13}C} gHSQC spectrum of the isolated LP shows the presence of 12 amino acids. A ^{1}H-^{13}C HMBC cross peak between Ser9 H$^\beta$ and Ser12C' confirms the formation of a four-residue macrocycle. Additionally, the high chemical shift of the Ser9 CH$^\beta$ independently confirms that the C-terminal ester bond is formed with this residue. **D:** Amide region of a ^{1}H-^{1}H TOCSY spectrum, showing all amino acids in the isolated LP.

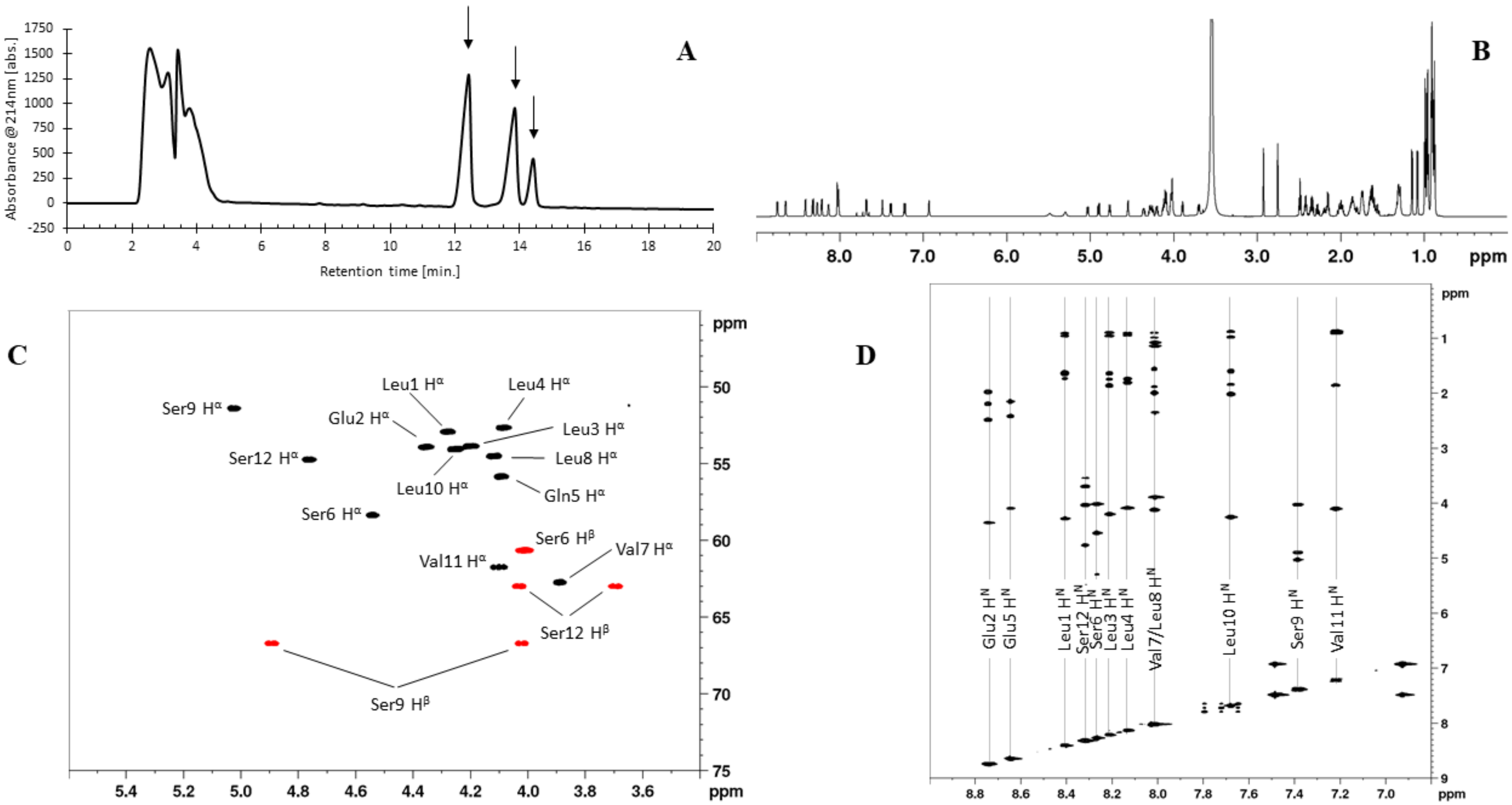

Figure S15. Characterization of tensin produced by *P. zeae* OE 48.2T. **A:** Representative preparative HPLC chromatogram of the medium extract of OE 48.2T. The LP that was analyzed is indicated by an arrow. Gradient: 25:75 to 0:100 H_2O:ACN in 20 minutes. **B:** 1D ^{1}H NMR spectrum of tensin eluting at 8.7 minutes. **C:** The alpha region of a ^{1}H-{^{13}C} gHSQC spectrum of the isolated LP shows the presence of 11 amino acids. The high chemical shift of the Thr3 CH^{β} indicates that the C-terminal ester bond is formed with this residue. **D:** Amide region of a ^{1}H-^{1}H TOCSY spectrum, showing all amino acids in the isolated LP.

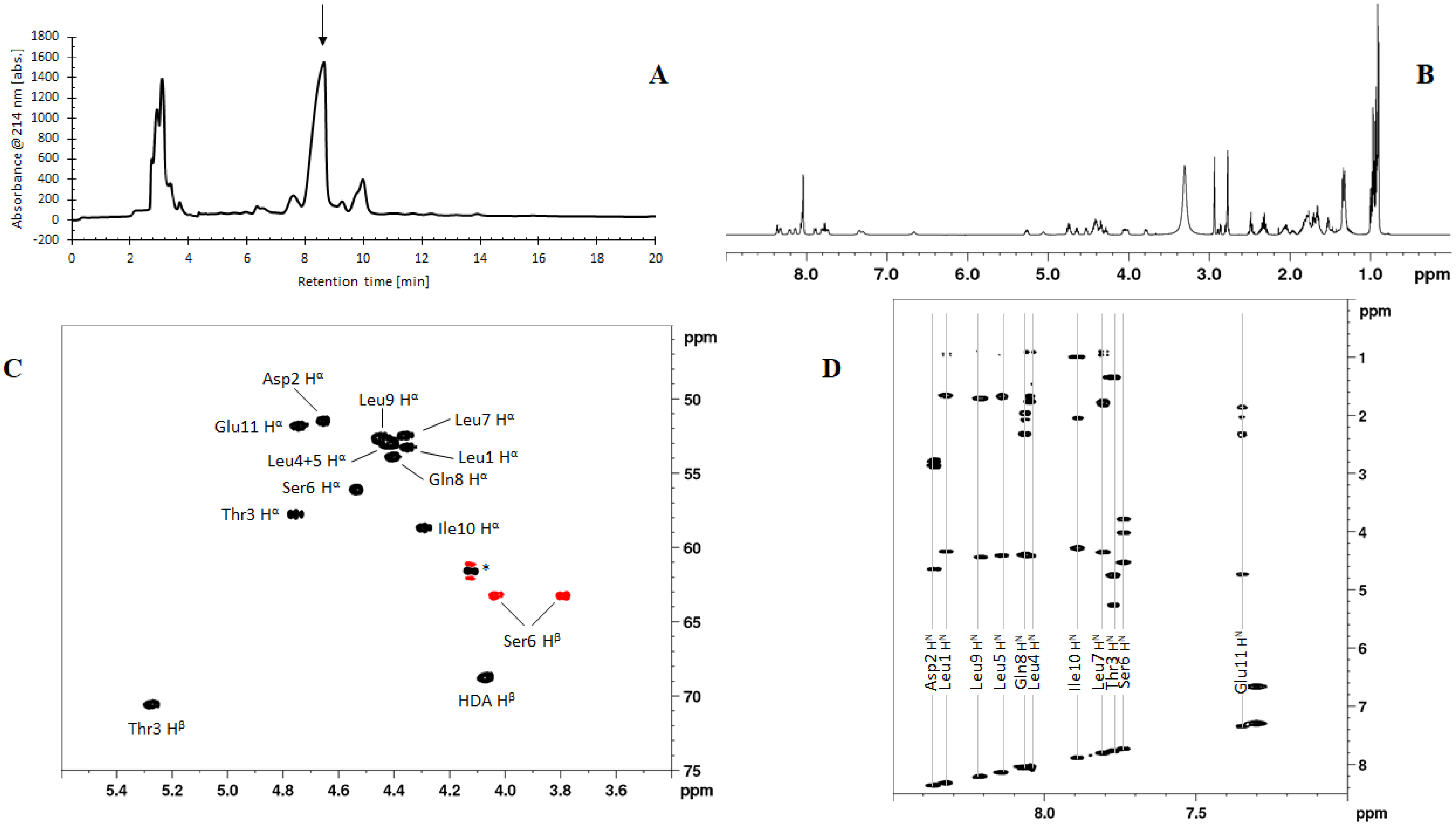

Figure S16. NMR spectral matching approach to verify structural similarity to a reference compound. A) The extracted compound of *P. zeae* OE 48.2^{T} (^{1}H-^{13}C HSQC spectrum, 700MHz) is identical to tensin (plotted chemical shifts according to (48)). B) Significant differences occur compared to the NMR spectral fingerprint of milkisin (plotted chemical shifts according to (49)). All spectra are recorded under identical conditions (acetone-d6, 298K).

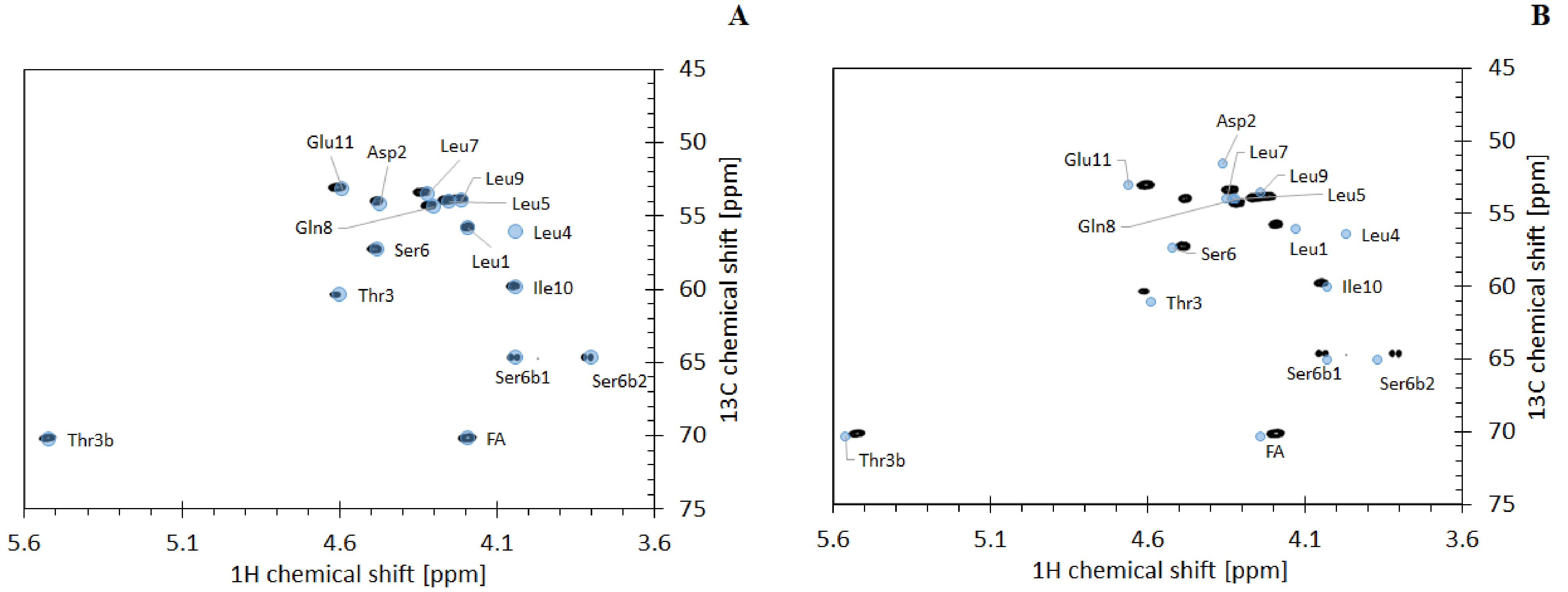

Supplemental references

1 de Bruijn I, Raaijmakers JM. 2009. Regulation of cyclic lipopeptide biosynthesis in *Pseudomonas fluorescens* by the ClpP protease. J Bacteriol 191:1910–1923.

2 Miller NT, Fuller D, Couger MB, Bagazinski M, Boyne P, Devor RC, Hanafy RA, Budd C, French DP, Hoff WD, Youssef N. 2016. Draft genome sequence of *Pseudomonas moraviensis* strain Devor implicates metabolic versatility and bioremediation potential. Genomics Data 9:154–159.

3 Hesse C, Schulz F, Bull CT, Shaffer BT, Yan Q, Shapiro N, Hassan KA, Varghese N, Elbourne LDH, Paulsen IT, Kyrpides N, Woyke T, Loper JE. 2018. Genome-based evolutionary history of *Pseudomonas* spp. Environ Microbiol 20:2142–2159.

4 Omoboye OO, Geudens N, Duban M, Chevalier M, Flahaut C, Martins JC, Leclère V, Oni FE, Höfte M. 2019. *Pseudomonas* sp. COW3 produces new bananamide-type cyclic lipopeptides with antimicrobial activity against *Pythium myriotylum* and *Pyricularia oryzae*. Molecules 24:4170.

5 Lopes LD, Davis EW, Pereira E Silva M de C, Weisberg AJ, Bresciani L, Chang JH, Loper JE, Andreote FD. 2018. Tropical soils are a reservoir for fluorescent *Pseudomonas* spp. biodiversity. Environ Microbiol 20:62–74.

6 Ma L, Qu S, Lin J, Jia J, Baird SM, Jiang N, Li H, Hou L, Lu S-E. 2019. The complete genome of the antifungal bacterium *Pseudomonas* sp. strain MS82. J Plant Dis Prot 126:153–160.

7 Zarvandi S, Bahrami T, Pauwels B, Asgharzadeh A, Hosseini-Mazinani M, Salari F, Girard L, De Mot R, Rokni-Zadeh H. 2020. Draft genome sequence of cyclic lipopeptide producer *Pseudomonas* sp. strain SWRI103, isolated from wheat rhizosphere. Microbiol Resour Announc 9:e00538-20.

8 Lopez JR, Dieguez AL, Doce A, De la Roca E, De la Herran R, Navas JI, Toranzo AE, Romalde JL. 2012. *Pseudomonas baetica* sp. nov., a fish pathogen isolated from wedge sole, *Dicologlossa cuneata* (Moreau). Int J Syst Evol Microbiol 62:874–882.

9 Beaton A, Lood C, Cunningham-Oakes E, MacFadyen A, Mullins AJ, Bestawy WE, Botelho J, Chevalier S, Coleman S, Dalzell C, Dolan SK, Faccenda A, Ghequire MGK, Higgins S, Kutschera A, Murray J, Redway M, Salih T, da Silva AC, Smith BA, Smits N, Thomson R, Woodcock S, Welch M, Cornelis P, Lavigne R, van Noort V, Tucker NP. 2018. Community-led comparative genomic and phenotypic analysis of the aquaculture pathogen *Pseudomonas baetica* a390T sequenced by Ion semiconductor and Nanopore technologies. FEMS Microbiol Lett 365:fny069.

10 McCully LM, Bitzer AS, Spence CA, Bais HP, Silby MW. 2014. Draft genome sequence of rice isolate *Pseudomonas chlororaphis* EA105. Genome Announc 2:e01342-14.

11 Spence CA, Lakshmanan V, Donofrio N, Bais HP. 2015. Crucial roles of abscisic acid biogenesis in virulence of rice blast fungus *Magnaporthe oryzae*. Front Plant Sci 6:1082.

12 Mavrodi OV, Walter N, Elateek S, Taylor CG, Okubara PA. 2012. Suppression of *Rhizoctonia* and *Pythium* root rot of wheat by new strains of *Pseudomonas*. Biol Control 62:93–102.

13 Gutiérrez-García K, Bustos-Díaz ED, Corona-Gómez JA, Ramos-Aboites HE, Sélem-Mojica N, Cruz-Morales P, Pérez-Farrera MA, Barona-Gómez F, Cibrián-Jaramillo A. 2019. Cycad coralloid roots contain bacterial communities including *Cyanobacteria* and *Caulobacter* spp. that encode niche-specific biosynthetic gene clusters. Genome Biol Evol 11:319–334.

14 Kwon SW, Kim JS, Park IC, Yoon SH, Park DH, Lim CK, Go SJ. 2003. *Pseudomonas koreensis* sp. nov., *Pseudomonas umsongensis* sp. nov. and *Pseudomonas jinjuensis* sp. nov., novel species from farm soils in Korea. Int J Syst Evol Microbiol 53:21–27.

15 Torres-Cortés G, Garcia BJ, Compant S, Rezki S, Jones P, Préveaux A, Briand M, Roulet A, Bouchez O, Jacobson D, Barret M. 2019. Differences in resource use lead to coexistence of seed-transmitted microbial populations. Sci Rep 9:6648.

16 Lozano GL, Bravo JI, Handelsman J. 2017. Draft genome sequence of *Pseudomonas koreensis* CI12, a *Bacillus cereus* "Hitchhiker" from the soybean rhizosphere. Genome Announc 5:e00570-17.

17 Lin H, Hu S, Liu R, Chen P, Ge C, Zhu B, Guo L. 2016. Genome sequence of *Pseudomonas koreensis* CRS05-R5, an antagonistic bacterium isolated from rice paddy field. Front Microbiol 7:1756.

18 Sazinas P, Aune MI, Fischer MH, Lauritsen JG, Gram L, Jelsbak L. 2019. Complete genome sequence of a bioactive *Pseudomonas* sp. strain, DTU12.3, isolated from soil in Denmark. Microbiol Resour Announc 8:e00121-19.

19 Dirksen P, Marsh SA, Braker I, Heitland N, Wagner S, Nakad R, Mader S, Petersen C, Kowallik V, Rosenstiel P, Félix M-A, Schulenburg H. 2016. The native microbiome of the nematode *Caenorhabditis elegans*: gateway to a new host-microbiome model. BMC Biol 14:38.

20 Gan HY, Gan HM, Savka MA, Triassi AJ, Wheatley MS, Smart LB, Fabio ES, Hudson AO. 2014. Whole-genome sequences of 13 endophytic bacteria isolated from shrub willow (salix) grown in geneva, new york. Genome Announc 2:e00288-14.

21 Tran PN, Tan NEH, Lee YP, Gan HM, Polter SJ, Dailey LK, Hudson AO, Savka MA. 2015. Whole-genome sequence and classification of 11 endophytic bacteria from poison ivy (*Toxicodendron radicans*). Genome Announc 3:e01319-15.

22 Kuzmanović N, Eltlbany N, Ding G, Baklawa M, Min L, Wei L, Smalla K. 2018. Analysis of the genome sequence of plant beneficial strain *Pseudomonas* sp. RU47. J Biotechnol 281:183–192.

23 Han Y, Dai B, Zhou Y, Wu Z, Ye B-C. 2017. Draft genome sequence of the plant growth-promoting bacterium *Pseudomonas* sp. SCPG-7, isolated from saline soil. Genome Announc 5:e00702-17.

24 Chauhan A, Pathak A, Ewida AYI, Griffiths Z, Stothard P. 2016. Whole genome sequence analysis of an Alachlor and Endosulfan degrading *Pseudomonas* strain W15Feb9B isolated from Ochlockonee River, Florida. Genomics Data 8:134–138.

25 Haney CH, Wiesmann CL, Shapiro LR, Melnyk RA, O'Sullivan LR, Khorasani S, Xiao L, Han J, Bush J, Carrillo J, Pierce NE, Ausubel FM. 2018. Rhizosphere-associated *Pseudomonas* induce systemic resistance to herbivores at the cost of susceptibility to bacterial pathogens. Mol Ecol 27:1833–1847.

26 Kim D, Lee H. 2019. Draft genome sequence of humic substances-degrading *Pseudomonas kribbensis* CHA-19 from temperate forest soil. Korean J Microbiol 55:177–179.

27 Chang D-H, Rhee M-S, Kim J-S, Lee Y, Park MY, Kim H, Lee S-G, Kim B-C. 2016. *Pseudomonas kribbensis* sp. nov., isolated from garden soils in Daejeon, Korea. Antonie Van Leeuwenhoek 109:1433–1446.

28 Yi L, Tang C, Peng Q, Peng Q, Chai L. 2015. Draft genome sequence of perfluorooctane acid-degrading bacterium *Pseudomonas parafulva* YAB-1. Genome Announc 3:e00935-15.

29 Peña A, Busquets A, Gomila M, Mulet M, Gomila RM, Reddy TBK, Huntemann M, Pati A, Ivanova N, Markowitz V, García-Valdés E, Göker M, Woyke T, Klenk H-P, Kyrpides N, Lalucat J. 2016. High quality draft genome sequences of *Pseudomonas fulva* DSM 17717(T), *Pseudomonas parafulva* DSM 17004(T) and *Pseudomonas cremoricolorata* DSM 17059(T) type strains. Stand Genomic Sci 11:55.

30 Midha S, Bansal K, Sharma S, Kumar N, Patil PP, Chaudhry V, Patil PB. 2016. Genomic resource of rice seed associated bacteria. Front Microbiol 6:1551.

31 Nesme J, Cania B, Zadel U, Schöler A, Płaza GA, Schloter M. 2017. Complete genome sequences of two plant-associated *Pseudomonas putida* isolates with increased heavy-metal tolerance. Genome Announc 5:e01330-17.

32 Frasson D, Opoku M, Picozzi T, Torossi T, Balada S, Smits THM, Hilber U. 2017. *Pseudomonas wadenswilerensis* sp. nov. and *Pseudomonas reidholzensis* sp. nov., two novel species within the *Pseudomonas putida* group isolated from forest soil. Int J Syst Evol Microbiol 67:2853–2861.

33 Rutz D, Frasson D, Sievers M, Blom J, Rezzonico F, Pothier JF, Smits THM. 2019. High-quality draft genome sequence of *Pseudomonas reidholzensis* strain CCOS 865T. Microbiol Resour Announc 8:e01502-18.

34 Wu L, Xiao W, Chen G, Song D, Khaskheli MA, Li P, Zhang S, Feng G. 2018. Identification of *Pseudomonas mosselii* BS011 gene clusters required for suppression of rice blast fungus *Magnaporthe oryzae*. J Biotechnol 282:1–9.

35 Zhang D, Xu D-H, Qiu J, Rasmussen-Ivey CR, Liles MR, Beck BH. 2016. Draft genome sequence of *Pseudomonas mosselii* Gil3, isolated from catfish and antagonistic against hypervirulent *Aeromonas hydrophila*. Genome Announc 4:e01305-16.

36 Sun D, Zhuo T, Hu X, Fan X, Zou H. 2017. Identification of a *Pseudomonas putida* as biocontrol agent for tomato bacterial wilt disease. Biol Control 114:45–50.

37 Guo J, Jing X, Peng W-L, Nie Q, Zhai Y, Shao Z, Zheng L, Cai M, Li G, Zuo H, Zhang Z, Wang R-R, Huang D, Cheng W, Yu Z, Chen L-L, Zhang J. 2016. Comparative genomic and functional analyses: unearthing the diversity and specificity of nematicidal factors in *Pseudomonas putida* strain 1A00316. Sci Rep 6:29211.

38 Park G-S, Chu J-H, Hong S-J, Kwak Y, Khan AR, Jung BK, Ullah I, Shin J-H. 2014. Complete genome sequence of the caprolactam-degrading bacterium *Pseudomonas mosselii* SJ10 isolated from wastewater of a nylon 6 production plant. J Biotechnol 192 Pt A:263–264.

39 Smits THM, Pothier JF, Ruinelli M, Blom J, Frasson D, Koechli C, Fabbri C, Brandl H, Duffy B, Sievers M. 2015. Complete genome sequence of the cyanogenic phosphate-solubilizing *Pseudomonas* sp. strain CCOS 191, a close relative of *Pseudomonas mosselii*. Genome Announc 3:e00616-15.

40 Weingarten RA, Johnson RC, Conlan S, Ramsburg AM, Dekker JP, Lau AF, Khil P, Odom RT, Deming C, Park M, Thomas PJ, NISC Comparative sequencing program, Henderson DK, Palmore TN, Segre JA, Frank KM. 2018. Genomic analysis of hospital plumbing reveals diverse reservoir of bacterial plasmids conferring carbapenem resistance. mBio 9:e02011-17.

41 Urón P, Giachini AJ, Glick BR, Rossi MJ, Nascimento FX. 2018. Near-Complete Genome sequence of *Pseudomonas palleroniana* MAB3, a beneficial 1-aminocyclopropane-1-carboxylate deaminase-producing bacterium able to promote the growth of mushrooms and plants. Genome Announc 6:e00242-18.

42 Gamez RM, Rodríguez F, Ramírez S, Gómez Y, Agarwala R, Landsman D, Mariño-Ramírez L. 2016. Genome sequence of the banana plant growth-promoting rhizobacterium *Pseudomonas fluorescens* PS006. Genome Announc 4:e00329-16.

43 Ertekin E, Hatt JK, Konstantinidis KT, Tezel U. 2016. Similar microbial consortia and genes are involved in the biodegradation of benzalkonium chlorides in different environments. Environ Sci Technol 50:4304–4313.

44 Ebadzadsahrai G, Thomson J, Soby S. 2018. Draft genome sequence of *Pseudomonas* sp. strain MWU12-2534b, isolated from a wild cranberry bog in Truro, Massachusetts. Microbiol Resour Announc 7:e01005-18.

45 Berti AD, Greve NJ, Christensen QH, Thomas MG. 2007. Identification of a biosynthetic gene cluster and the six associated lipopeptides involved in swarming motility of *Pseudomonas syringae* pv. *tomato* DC3000. J Bacteriol 189:6312–6323.

46 de Bruijn I, de Kock MJD, Yang M, de Waard P, van Beek TA, Raaijmakers JM. 2007. Genome-based discovery, structure prediction and functional analysis of cyclic lipopeptide antibiotics in *Pseudomonas* species. Mol Microbiol 63:417–428.

47 Thrane C, Harder Nielsen T, Neiendam Nielsen M, Sørensen J, Olsson S. 2000. Viscosinamide-producing *Pseudomonas fluorescens* DR54 exerts a biocontrol effect on *Pythium ultimum* in sugar beet rhizosphere. FEMS Microbiol Ecol 33:139–146.

48 Rokni-Zadeh H, Li W, Sanchez-Rodriguez A, Sinnaeve D, Rozenski J, Martins JC, De Mot R. 2012. Genetic and functional characterization of cyclic lipopeptide white-line-inducing principle (WLIP) production by rice rhizosphere isolate *Pseudomonas putida* RW10S2. Appl Environ Microbiol 78:4826–4834.

49 Rokni-Zadeh H, Li W, Yilma E, Sanchez-Rodriguez A, De Mot R. 2013. Distinct lipopeptide production systems for WLIP (white line-inducing principle) in *Pseudomonas fluorescens* and *Pseudomonas putida*. Environ Microbiol Rep 5:160–169.

50 Ma Z, Ongena M, Höfte M. 2017. The cyclic lipopeptide orfamide induces systemic resistance in rice to *Cochliobolus miyabeanus* but not to *Magnaporthe oryzae*. Plant Cell Rep 36:1731–1746.

51 Ma Z, Geudens N, Kieu NP, Sinnaeve D, Ongena M, Martins JC, Höfte M. 2016. Biosynthesis, chemical structure, and structure-activity relationship of orfamide lipopeptides produced by *Pseudomonas protegens* and related species. Front Microbiol 7:382.

52 Gross H, Stockwell VO, Henkels MD, Nowak-Thompson B, Loper JE, Gerwick WH. 2007. The genomisotopic approach: a systematic method to isolate products of orphan biosynthetic gene clusters. Chem Biol 14:53–63.

53 D'aes J, Kieu NP, Léclère V, Tokarski C, Olorunleke FE, De Maeyer K, Jacques P, Höfte M, Ongena M. 2014. To settle or to move? The interplay between two classes of cyclic lipopeptides in the biocontrol strain *Pseudomonas* CMR12a. Environ Microbiol 16:2282–2300.

54 Weisshoff H, Hentschel S, Zaspel I, Jarling R, Krause E, Pham TLH. 2014. PPZPMs - a novel group of cyclic lipodepsipeptides produced by the *Phytophthora alni* associated strain *Pseudomonas* sp. JX090307- the missing link between the viscosin and amphisin group. Nat Prod Commun 9:989–996.

55 Omoboye OO. 2019. Cyclic lipopeptide diversity and biocontrol versatility of *Pseudomonas* species associated with the cocoyam rhizosphere. PhD thesis, Ghent University. Faculty of Bioscience Engineering, Ghent, Belgium.

56 Oni FE, Geudens N, Onyeka JT, Olorunleke OF, Salami AE, Omoboye OO, Arias AA, Adiobo A, De Neve S, Ongena M, Martins JC, Höfte M. 2020. Cyclic lipopeptide-producing *Pseudomonas koreensis* group strains dominate the cocoyam rhizosphere of a *Pythium* root rot suppressive soil contrasting with *P . putida* prominence in conducive soils. Environ Microbiol 22:5137-5155.

57 Oni FE, Geudens N, Omoboye OO, Bertier L, Hua HGK, Adiobo A, Sinnaeve D, Martins JC, Höfte M. 2019. Fluorescent *Pseudomonas* and cyclic lipopeptide diversity in the rhizosphere of cocoyam (*Xanthosoma sagittifolium*). Environ Microbiol 21:1019–1034.
